# Impact of the WellCheck smartphone app linked to electronic health records on clinical outcomes in patients with type 2 diabetes: Study protocol for primary care-based, prospective, multicenter, cluster-randomized, pragmatic clinical trials

**DOI:** 10.1371/journal.pone.0329003

**Published:** 2025-08-07

**Authors:** Hyunji Sang, Sunyoung Kim, Jiyoung Hwang, Selin Woo, Yerin Hwang, Jaewon Kim, Seohei Lee, Dong Keon Yon, Sang Youl Rhee

**Affiliations:** 1 Department of Endocrinology and Metabolism, Kyung Hee University Medical Center, Kyung Hee University College of Medicine, Seoul, South Korea,; 2 Center for Digital Health, Medical Science Research Institute, Kyung Hee University Medical Center, Kyung Hee University College of Medicine, Seoul, South Korea; 3 Department of Family Medicine, Kyung Hee University Medical Center, Kyung Hee University College of Medicine, Seoul, South Korea; 4 Yonsei Best Internal Medicine, Goyang-si, Gyeonggi-do, South Korea; 5 Department of Regulatory Science, Kyung Hee University, Seoul, South Korea; 6 Department of Precision Medicine, Kyung Hee University College of Medicine, Seoul, South Korea; 7 Department of Pediatrics, Kyung Hee University Medical Center, Kyung Hee University College of Medicine, Seoul, South Korea; Japanese Academy of Health and Practice, JAPAN

## Abstract

Diabetes, obesity, and dyslipidemia are significant contributors to cardiovascular diseases, necessitating effective strategies to manage blood glucose, blood pressure, and lipid levels through lifestyle interventions and pharmacotherapy. This study aims to assess the glycemic control and metabolic health outcomes achieved over 24 weeks with the use of the “WellCheck” digital healthcare application in patients diagnosed with type 2 diabetes mellitus (T2D) within real-world primary care settings. This is a primary care-based, prospective, multicenter, cluster-randomized pragmatic trial. Eligible participants include adults aged 19–80 with T2D who are treated with or planning to be treated with Envlo (enavogliflozin) or Envlomet (enavogliflozin/metformin). WellCheck links a mobile application for patients with a web platform for physicians and integrates with electronic health records to support the management of diabetes and hypertension. The primary outcome is the change in glycated hemoglobin levels at 24 weeks from baseline. We will conduct cluster randomization by grouping 24 participating primary care centers according to basic characteristics, such as the treating physician’s age (above or below 45 years) and the clinic’s location (metropolitan or non-metropolitan areas). The “App+Physician Support” group will use the WellCheck app for comprehensive digital monitoring and receive feedback supported by an investigator (primary care physician), and the “App-Only” group will rely on the WellCheck app for independent self-management without direct physician assistance. This study aims to explore the effectiveness of WellCheck app in improving glycemic control and metabolic outcomes, providing valuable insights into its potential as a digital tool for managing T2D. The wide age range of participants and cluster randomization of primary care centers into the intervention and control groups enhance the generalizability and robustness of the study, reducing bias and enabling a meaningful comparison of outcomes in diverse healthcare settings.

**Trial registration:**

ClinicalTrials.gov NCT06419816. Registered on 14 May 2024.

## Introduction

Diabetes, obesity, and dyslipidemia are significant metabolic disorders and key risk factors associated with cardiovascular diseases such as heart attack, stroke, and hypertension. According to the International Diabetes Federation (IDF), the global diabetic population has increased from 425 million in 2017 to 463 million in 2019 [1,2], with projections suggesting an explosive increase of over 783 million by 2045 [3]. In Korea, factors such as westernized dietary habits due to economic growth and elevated income, lack of exercise, and extended life expectancy have led to a rapid escalation in diabetes cases [4] from 3 million in 2007 to 6.05 million in 2020 [5], expected to reach 5.4 million (10.85% of the total population) by 2030 [6].

Treatment generally follows the American Diabetes Association (ADA) guidelines, which recommend achieving glycated hemoglobin (HbA1c) levels below 7.0% [7]. The Korean Diabetes Treatment Guidelines suggest a general glycemic control target of HbA1c < 6.5% for patients with type 2 diabetes (T2D) [8]. The United Kingdom Prospective Diabetes Study (UKPDS) found that a 1% reduction in HbA1c levels decreased the incidence of microvascular complications by 37% and myocardial infarction by 14% [9]. Strict and aggressive glucose control is reportedly the most effective method for preventing the onset of diabetic complications and slowing the progression of existing complications.

All T2D patients should be educated on lifestyle modifications upon diagnosis [10]. Diabetes Self-management Education and Support (DSMES) aims to improve outcomes by empowering patients to manage their conditions effectively, enhancing their quality of life [10]. Information and communication technology usage in healthcare is expanding rapidly worldwide [11], and various digital health coaching methods, including text messaging, mobile applications, and web-based algorithms, have been reported to improve glycemic control and increase self-management among individuals with diabetes [12].

However, the efficacy of digital solutions in real-world clinical settings remains unclear. While some reviews have shown the applicability of remote healthcare in effectively reducing blood pressure, others have not demonstrated these outcomes consistently [13,14]. Digital healthcare applications for managing chronic conditions, such as diabetes and hypertension, have shown promise in improving service delivery, patient engagement, and self-management. However, decisive evidence on the impact of integrating these applications into hospital systems for disease management remains scarce [15]. This study aims to determine the real-world effects of a hospital-linked digital healthcare smartphone application (“WellCheck”) on blood glucose-lowering and diabetes-related metabolic markers in adult T2D patients under routine care.

## Materials and methods

### Study design

We designed this clinical trial as a primary care-based, prospective, multicenter, cluster-randomized, pragmatic trial to evaluate the effectiveness of the “WellCheck” application in improving glycemic control, blood pressure, and weight over 24 weeks among patients with T2D in the real-world setting of primary care practices.

### Study setting

**Fig 1:**
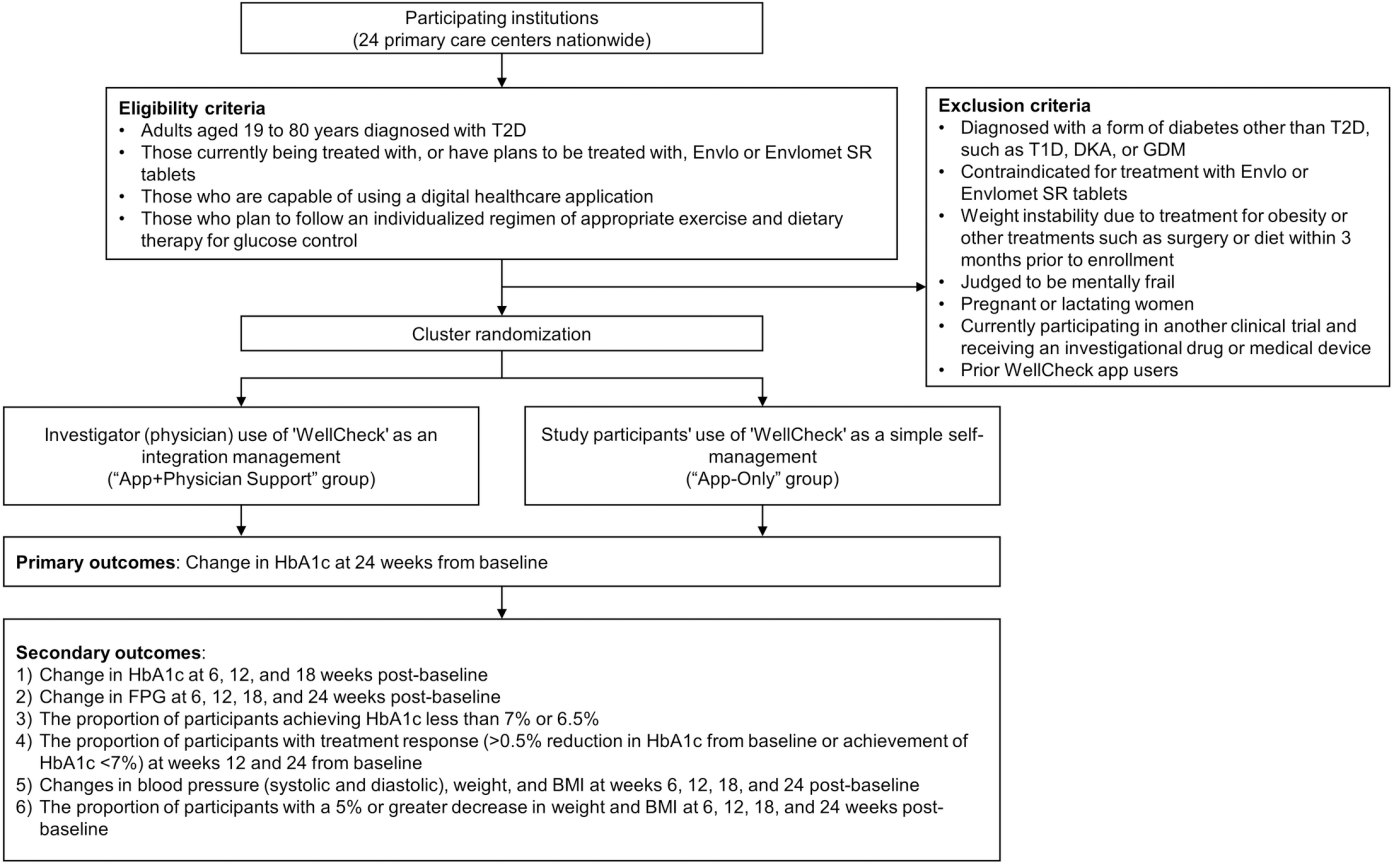
Study design and procedural flowchart. The flowchart illustrates participant recruitment based on defined inclusion and exclusion criteria, cluster randomization to either the “App+Physician Support” or “App-Only” groups, scheduled follow-up assessments (with optional visits indicated), and comprehensive outcome measurements throughout the 24-week study period. T2D, type 2 diabetes mellitus; SR, sustained release; HbA1c, glycated hemoglobin; T1D, type 1 diabetes mellitus; DKA, diabetic ketoacidosis; GDM, gestational diabetes; eGFR, estimated glomerular filtration rate; FPG, fasting plasma glucose; BMI, body mass index.

**Fig 1** presents the detailed flowchart outlining the overall design and procedures of the clinical study. The study will involve 24 primary care centers nationwide. Patients undergoing treatment or deemed eligible for treatment with Envlo (enavogliflozin) tablets or Envlomet (enavogliflozin/metformin) sustained-release (SR) tablets for T2D, based on the medical judgment of the researcher (attending physician) during routine clinical visits, and those who can use a digital healthcare smartphone application without difficulty, are eligible for enrollment in the study.

We plan to enroll participants from 11/06/2024 to 30/06/2026. Reflecting the practical realities of primary care practices, where it may be challenging to manage intervention and control groups separately within the same institution, the trial employs a cluster randomization method. This approach involves distinguishing institutions into “App+Physician Support” groups (institutions using the WellCheck application managed in conjunction with the researcher [attending physician]) and “App-Only” groups (institutions where participants use the WellCheck application for simple self-management) before proceeding with the trial (**Fig 1**).

This clinical trial will collect data from the medical records documented in real-world clinical settings. Information gathered will include demographic details, physical measurements, vital signs, and laboratory test results. The study protocol do not mandate obligatory visits, tests, or treatments. However, we have planned a prospective follow-up to collect electronic health record (EHR) data from the participants for up to 24 weeks after the enrollment date (baseline).

**Fig 2:**
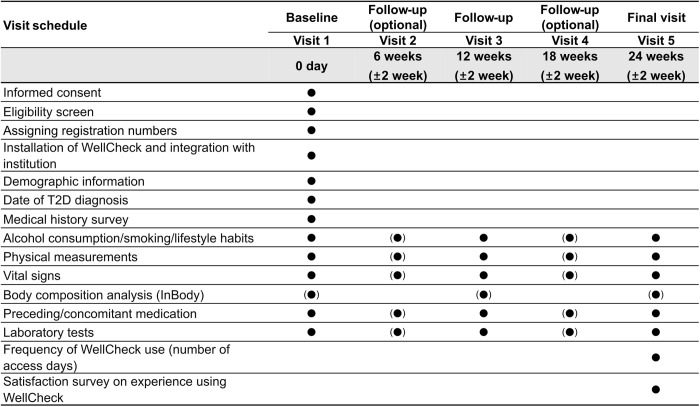
Timeline for clinical study visits and data collection. Visits are scheduled at baseline (Day 0), Week 6 (optional), Week 12, Week 18 (optional), and Week 24. Optional visits (Weeks 6 and 18) may involve data collection via telephone follow-ups, as needed. T2D, type 2 diabetes mellitus.

**Fig 2** clearly illustrates the scheduled prospective collection points for each participant’s EHR data. Data collection will occur at Visit 1 (Baseline, Day 0), Visit 2 (Follow-up, Week 6), Visit 3 (Follow-up, Week 12), Visit 4 (Follow-up, Week 18), and Visit 5 (Final, Week 24). Among these, Visits 2 and 4 are optional and subject to the researcher’s (attending physician’s) discretion, allowing for data collection via telephone follow-ups. In the case of study discontinuation/dropout, we will record the data collected up to that point as comprehensively as possible in the case report form.

The developed protocol follows the Standard Protocol Items: Recommendations for Interventional Trials (SPIRIT) 2013 Statement (S1 File) [16].

### Eligibility criteria

Participants eligible for this study are adults aged between 19 and 80 years diagnosed with T2D and are currently undergoing treatment or planning to receive treatment with an Envlo tablet or an Envlomet SR tablet according to approved indications. Additionally, these individuals should be capable of using a smartphone and have no difficulty using a digital healthcare application designed for smartphones. They should follow an individualized regimen of appropriate exercise and dietary therapy for glucose control during the trial period. Women of childbearing potential and men must agree to the use of effective contraception methods throughout the trial period or have no plans for pregnancy, such as hormonal contraceptives, intrauterine devices or systems, vasectomies, tubal ligation, or a double-barrier method, including the use of a cervical cap or contraceptive diaphragm and a male condom. Finally, eligible participants should receive a comprehensive explanation of the clinical trial, voluntarily consent to participate, and agree to comply with the trial instructions throughout the trial period (**Fig 1**).

We will exclude individuals from participation in this clinical study if they meet any of the following criteria: those diagnosed with forms of diabetes other than T2D, including type 1 diabetes mellitus, diabetic ketoacidosis, gestational diabetes, and others; patients for whom treatment with an Envlo tablet or Envlomet SR tablet is contraindicated according to approved indications; patients who have undergone treatments for obesity or other treatments such as surgery or diet that have resulted in unstable weight within the three months before enrollment; individuals considered psychosomatically weak; pregnant or breastfeeding women; individuals currently participating in another clinical trial and receiving investigational medicinal products or medical devices; individuals who have prior experience using the WellCheck application before study enrollment; and any other individuals deemed by the researcher (attending physician) to be inappropriate for participation in the trial based on their professional judgment (**Fig 1**).

### Overview of the WellCheck application

WellCheck, a hospital-connected digital health application, is different from other simple digital health applications because it connects the patient’s application to the doctor’s web and EHR to manage diabetes and hypertension. Doctors can use WellCheck to conveniently check their patient’s health status and manage it on the web for doctors [17]. WellCheck allows users to record blood sugar and other health issues through questionnaires and sends an alarm to the user at least once a week if blood sugar is unrecorded. WellCheck can then sent the data to physicians for analysis and monitoring. Users can also see how well they manage their blood sugar levels against their goals through the app. Previous studies have well-documented the process of using the WellCheck app and web [17].

Participants in the “App+Physician Support” group use the WellCheck application with active involvement from their healthcare providers (physicians) (**Fig 1**). In this group, the doctor can send feedback messages for management according to their judgment for too high or too low blood sugar levels of users compared with that of the target, allowing doctors to manage and monitor at-risk patients separately. In contrast, participants in the “App-Only” group utilize the same WellCheck application but without active physician engagement or direct feedback (**Fig 1**).

### Outcomes

The primary outcome is the change in HbA1c levels from baseline to 24 weeks (**Fig 1**). The secondary outcomes are 1) the change in HbA1c at 6, 12, and 18 weeks post-baseline; 2) the change in fasting plasma glucose (FPG) at 6, 12, 18, and 24 weeks post-baseline; and 3) the proportion of participants achieving HbA1c less than 7% or 6.5%; 4) the proportion of participants with treatment response (>0.5% reduction in HbA1c from baseline or achievement of HbA1c < 7%) at weeks 12 and 24 from baseline; changes in blood pressure (systolic and diastolic), weight, and body mass index (BMI) at weeks 6, 12, 18, and 24 post-baseline; 6) the proportion of participants with a 5% or greater decrease in weight and BMI at 6, 12, 18, and 24 weeks post-baseline (**Fig 1**).

The exploratory outcomes include assessing the changes in various health indicators at multiple time points compared with the baseline. Changes in lipid profiles, including total cholesterol, low-density lipoprotein cholesterol, high-density lipoprotein cholesterol (HDL-C), and triglyceride levels, will be observed at 6, 12, 18, and 24 weeks. Similarly, alterations in liver function markers, such as aspartate aminotransferase, alanine aminotransferase, and gamma-glutamyl transferase, as well as kidney function indicators, including estimated glomerular filtration rate, urine albumin to creatinine ratio, and urine glucose to creatinine ratio, will be assessed at these same intervals. Additionally, we will measure comprehensive changes in body composition analysis indicators, such as body fat mass, visceral fat mass, skeletal muscle mass, waist/hip circumference, and various hydration and fat ratio metrics at 12- and 24-week intervals. Finally, we will evaluate the variation in the risk of cardiovascular disease complications, assessed using the atherosclerotic cardiovascular disease (ASCVD) risk score at 12 and 24 weeks.

Safety outcomes consist of a comprehensive analysis of laboratory tests, vital signs, and physical examination results. Laboratory tests include various hematological and biochemical parameters to detect potential intervention-induced disorders. Routinely recorded vital sign measurements such as blood pressure, heart rate, respiratory rate, and body temperature identify deviations from normal ranges. A qualified medical professional conducts a physical examination to assess the participants’ health and detect clinical abnormalities.

### Sample size

Based on the results of a pilot study, the difference in mean HbA1c levels between the control group and those using the WellCheck application over 24 weeks in patients with T2D was 0.4% (8.0% vs. 7.6%), with a standard deviation (s) of 1.1%. Using these results and calculating with G*Power 3.1.9.7 [18], we estimated the required total sample size to be 137 subjects per group for a total of 274 subjects, with a corrected significance level of 5% and power of 85%.

Since this study employs cluster randomization, we further adjusted the sample size calculation by incorporating the design effect to account for potential intra-cluster correlations among patients within the same clinic. We calculated the design effect using the standard formula:


Design\ effect=1+(m−1)×ICC

Referring to previous studies conducted in primary care settings for patients with type 2 diabetes [19,20], we conservatively adopted an intraclass correlation coefficient (ICC) value of 0.02 and assumed an average cluster size (*m*) of 20 participants per center. Therefore, we calculated the resulting design effect to be 1.38, and applying this adjustment to the initial sample size estimate gives a revised total sample size of 378 participants. Finally, considering an anticipated dropout rate of approximately 20%, we adjusted the target recruitment number further to approximately 480 participants.

### Allocation

We will conduct cluster randomization by dividing the participating primary care centers into subgroups based on basic characteristics (e.g., age of the treating physician [above or below 45 years] and location [metropolitan or non-metropolitan areas]). Subsequently, we will randomly assign the intervention and control groups within these subgroups to conduct the trial.

An independent randomization officer not associated with this clinical trial will generate a randomization list using the Proc Plan procedure in SAS (version 9.4; SAS Institute, Cary, NC, USA) to ensure strict randomization. The developer of the interactive web response system (IWRS) will provide the generated list, and the IWRS will verify the randomization numbers.

We will evaluate screening test results after obtaining written informed consent from the participants for this clinical trial. We will assign a randomization number and randomize participants to either the intervention or control group in a 1:1 ratio only if they meet the inclusion criteria and do not fall under the exclusion criteria.

### Data collection

We will collect data in this study using a predesigned electronic case report form (eCRF), and all utilized electronic data capture (EDC) systems will comply with the U.S. Code of Federal Regulations (CFR) for processing and managing electronic data. Only authorized individuals can access the EDC system, a certified electronic data collection system. The system will record all the actions involving entering, modifying, saving, and deleting data in the eCRF. **Fig 2** presents the collection schedules for all data collection items.

**1) Obtaining written consent and assigning participant registration number**

Before collecting any data related to this study, the researchers will thoroughly explain the purpose and details of the study to potential participants using a participant information sheet. We will ask participants to provide voluntary consent and complete a consent form, including the participant’s name, signature, and date of signature. Although the dates of written consent and Visit 1 may differ, participants should have provided consent before participation in the clinical study commences. A unique registration number is automatically assigned in the eCRF on registration after obtaining participant consent.

**2) Inclusion and exclusion criteria verification**

We will verify the inclusion and exclusion criteria at Visit 1, confirming that the participants meet all the inclusion criteria, not the exclusion criteria.

**3) “WellCheck” app installation and registration**

Participants registered in the study must install the “WellCheck” smartphone application, complete the registration process, and then enter the institution code provided for research purposes to link the app with the hospital.

**4) Demographic Information**

At Visit 1, we will collect the participants’ basic information, including initials, sex, date of birth, age, and information on pregnancy and breastfeeding for verification. We will obtain pregnancy status through interviews or, if available, from pregnancy test results.

**5) T2D information**

At Visit 1, we will collect the date of T2D diagnosis from the participants.

**6) Medical history investigation**

We will define and record clinically significant medical conditions or abnormalities observed up to Visit 1. This includes the history of illnesses within six months before Visit 1 and any ongoing conditions, along with their diagnoses and dates.

**7) Alcohol consumption, smoking, and lifestyle information**

At Visit 1, we will collect basic information regarding the participants’ alcohol consumption, smoking habits, and lifestyle for initial verification, and we will record subsequent changes in these areas at all visits. We typically verify these items through interviews and medical records during regular medical visits, with the trial group also having the option to utilize data collected through the “WellCheck” application.

We will categorize participants based on their alcohol consumption as “Current drinker,” “Past drinker,” or “Never drinker.” We define “Current drinker” as having consumed 12 or more units of alcohol in their lifetime and having been drunk at least once in the past 12 months, “Past drinker” as having consumed 12 or more units of alcohol in their lifetime but not having been drunk in the past 12 months, and “Never drinker” as having consumed less than 12 units of alcohol in their lifetime [21]. We will record the number of drinks consumed per week and the amount of alcohol consumed in one sitting for current drinkers.

We categorize smoking history as “Current smoking,” “Past smoking,” or “Non-smoking.” We define “Current smoking” as having smoked 5 or more packs (100 cigarettes) in the lifetime and within the last 30 days, “Past smoking” as having smoked 5 or more packs (100 cigarettes) in the lifetime but not within the previous 30 days, and “Non-smoking” as having smoked less than 5 packs (100 cigarettes) in the lifetime [22].

Regarding lifestyle information, eating habits include irregular eating, overeating, and excess carbohydrates/sugar, fat, and salt intake. Exercise habits include the type of exercise (walking, cardio, or strength training), number of exercises per week, and their intensity (less than 30 min, less than an hour, or longer).

**8) Physical Measurements**

We will measure body weight and BMI at each visit. The CRF automatically calculates the BMI using height and weight measurements. We will measure height once during Visit 1.

**9) Vital Signs**

At every visit, we will record vital signs, including blood pressure (systolic/diastolic) and pulse rate. We will take blood pressure and pulse rate measurements after at least 5 min of rest in a quiet environment while sitting in a chair with back support. Participants should avoid smoking and consuming alcohol and caffeine for at least 30 min before the measurement.

**10) Body Composition Analysis**

We will conduct body composition analysis at available facilities during Visits 1, 3, and 5. The metrics include skeletal muscle mass, body fat mass, body fat percentage, muscle mass, waist-to-hip ratio, total body water, intracellular water, extracellular water, extracellular-to-intracellular water ratio, and visceral fat percentage.

**11) Concomitant and Preceding Medication**

For preceding medications, we will collect only diabetes treatments administered within four weeks before Visit 1. Regarding concomitant medications, we will collect information on all medicines taken consistently for more than three months during the study period after Visit 1, including those for treating T2D. The collected information includes the name of the medication (brand name), dosage and administration details (single dosage, unit, frequency of administration, and route of administration), duration of treatment (start date, end date, and whether ongoing), purpose of administration, and reasons for dose changes or discontinuation, if applicable.

**12) Laboratory Tests**

We will perform laboratory tests based on medical records from actual clinical environments and standard clinical practice. If HbA1c results collected within 4 weeks before Visit 1 and other test results collected within 3 months are available, these can substitute for laboratory tests at Visit 1.

We will analyze ASCVD risk post-study using the ASCVD 2013 Risk Calculator from the American Heart Association/American College of Cardiology [23] based on information collected in the eCRF (age, sex, systolic blood pressure, total cholesterol, HDL-C, treatment for hypertension, diagnosis of diabetes, and smoking status). For the trial group, the WellCheck application’s automatic calculation feature allows researchers (attending physicians) to input cholesterol and blood pressure values during consultations with participants.

**13) WellCheck application user experience satisfaction survey**

At Visit 5, we will conduct a satisfaction survey on the WellCheck application’s user experience by healthcare professionals and participants. We specifically tailored the WellCheck user satisfaction questionnaires for this study, incorporating core elements from existing validated tools designed to assess patient satisfaction and evaluate mHealth apps. We developed the questionnaires by referencing key literature addressing domains such as patient-provider communication, participants’ acceptance and understanding of health education, self-management support, and overall treatment quality [24–26]. This survey comprises 10 questions and utilizes a 5-point scale to assess general satisfaction with the WellCheck application. It focuses on the effectiveness of chronic disease management and improvements in work efficiency resulting from the application’s use, not on specific diseases.

The 10 questions in the WellCheck user experience satisfaction survey for healthcare professionals evaluate the improvement in managing chronic diseases and enhancing the efficiency of medical tasks with the application. The 5-point scale ranges from 1) Strongly Disagree, 2) Disagree, 3) Neutral, 4) Agree, to 5) Strongly Agree (S1 Table).

The 10 questions for the participants aim to assess whether the application contributed to their satisfaction with their relationship with healthcare providers and their experience of the treatment process. The scale is structured similarly, ranging from 1) Strongly Disagree to 5) Strongly Agree (S2 Table).

In cases where participants discontinue or withdraw from the study, the researcher will record the data collected up to that point in the eCRF.

### Data management

Periodic audits will confirm the quality of collected data. If discrepancies occur between the eCRF and source documents, with inappropriate entries or logical inconsistencies, the sponsor or data management personnel will collaborate with the principal investigator to review the validity of the concerned items. Corrections through documentation will ensure the accuracy of the records. Once the sponsor confirms that the eCRF and database are error-free, the database will be locked to prevent accidental or unauthorized changes in the data.

The sponsor has the right to request the verification or modification of the collected data during the data processing phase. Upon receiving such requests, researchers must recheck or amend the data accordingly to ensure that their responses align with the request. This guarantees that the data entered into the eCRF through electronic signatures are accurate, complete, decipherable, and timely. The eCRFs created via the EDC system will be copied to electronic storage media and distributed to each research institution upon the conclusion of the study.

The principal investigator is responsible for storing and managing all data and records collected at each institution, including data of participants who withdrew their consent or dropped out midway. All documents collected during the study period must be stored in a secure location accessible only to the principal investigator, co-investigators, and designated research personnel and equipped with security measures to prevent unauthorized access. The principal investigator must retain these documents for three years from the date of study completion, with the possibility of extending this period, if necessary, by the sponsor. These documents may be subject to inspection by the sponsors or relevant regulatory authorities. Without written permission from the sponsor, the researchers should not destroy the documents related to this study. Upon the expiration of the retention period, in consultation with the sponsor, the researchers will immediately shred paper documents, and destroy electronic records so that they cannot be restored or regenerated.

### Statistical methods

Descriptive statistics will describe continuous variables (number of subjects, mean, standard deviation, median, minimum, and maximum) and categorical variables presented as frequencies and percentages. To assess baseline comparability between intervention and control groups, we will calculate standardized mean differences (SMDs) for key baseline characteristics, including age, sex, HbA1c, BMI, and diabetes duration. We will explicitly include variables exhibiting significant imbalance between groups (SMD > 0.1) as covariates in subsequent analyses [27].

Given our cluster-randomized design, we will analyze primary, secondary, exploratory, and safety outcomes using mixed-effects models or generalized estimating equations (GEE), which are robust methods specifically designed for clustered data [28,29]. Mixed-effects models will include patient-level covariates as fixed effects, and inter-center variability as random effects [30]. We will adjust multiple comparisons within exploratory outcomes—such as measures of lipids, liver and kidney function, body composition, and ASCVD risk—using the Benjamini–Hochberg false discovery rate method, with significance defined at q ≤ 0.05 [31].

To robustly handle potential missing data due to optional visits (Weeks 6 and 18) and telephone-based data collection, we will first evaluate the extent and pattern of missingness across groups. If missing data is minimal and evenly distributed, we will consider a complete-case analysis as an initial approach. However, if missing data is substantial or disproportionately distributed between groups, we will employ multiple imputation methods—specifically, multiple imputation by chained equations (MICE)—a widely-used method in healthcare research [32]. Additionally, we will perform sensitivity analyses to ensure the robustness and validity of our conclusions under various missing-data scenarios.

A two-sided approach, with a significance level of 5%, will be used for all tests [33–35]. If a subgroup analysis based on participant characteristics is necessary, we can analyze each item in the same manner as the efficacy and safety outcome variables (e.g., analysis of primary outcomes by sex). Additionally, we will perform subgroup or sensitivity analyses based on diabetes duration to investigate whether and how the length of time since diabetes diagnosis influences the observed outcomes.

### Data monitoring

The sponsor will conduct monitoring to protect the rights and welfare of the research participants, the accuracy, completeness, and verifiability of the study data reported by the principal investigator through comparison with source documents, and ensure the conduction of the study according to the approved protocol and relevant regulations. Monitoring personnel designated by the sponsor will monitor the study through regular visits and telephone communications by at the study institutions. During the visits, the monitoring personnel will primarily review the source documents, management records of the study software, and the storage status of essential study documents. They will also check the progress procedures and records of the study, discuss any issues such as violations with the principal investigator and study personnel, and ensure that appropriate modifications and actions are taken.

### Patient and public involvement

Patients did not participate in the study design and will not participate in the recruitment or conduct of the study.

### Ethics and dissemination

The Public Institutional Review Board (IRB) designated by Ministry of Health and Welfare approved the protocol (IRB no. P01-202405-01-016, approved on May 14, 2024).

The researcher should obtain informed consent from the participants based on the ethical principles of the Declaration of Helsinki and the Bioethics and Safety Act standards. The researcher thoroughly explains the research to the participants (or legal representatives) and obtains written consent before beginning any research-related procedures. We receive consent privately (e.g., in an office or consultation room). Considering that participants may vary widely in digital literacy and technology accessibility, particularly among older adults, our informed consent process explicitly addresses these factors. During participant enrollment, trained research personnel will thoroughly explain the digital requirements of the study and assess each participant’s comfort and familiarity with smartphone use and app navigation. Research staff will actively address and manage participants’ ongoing questions and concerns regarding digital technology use by throughout the study duration to ensure inclusivity and equal access.

A locked laboratory will store the research data. The principal investigator is responsible for the participants’ medical record numbers and institutional registration numbers in a separate file and encrypted to prevent identification from the clinical study data. According to the Enforcement Rule of Bioethics and Safety Act, we will retain records related to clinical research for three years after the completion of the study, and we will destroy documents containing personal information after the retention period, as per the Personal Information Protection Act. Researchers will password-protect files containing personal information, and any publication of clinical study results will exclude information that could potentially identify patients.

Researchers will verify the health status of each participant before enrolling them in the clinical trial to ensure their suitability for participation. Treatment and care for participants’ conditions must proceed independently of the trial, with the necessary medical treatment and care provided under clinical judgment during and after the trial period. This trial does not present any risks beyond those associated with routine care in a clinical setting; thus, there is no need for additional compensation for study-related risks. Medical law, the principal investigator’s professional indemnity insurance, and related institutional policies adequately protect the participants and researchers. The existing legal liabilities of medications govern the compensation for pharmaceuticals.

The sponsor owns all the data and results obtained through the study, and with the sponsor’s approval, the researchers will present the study results as academic papers at national and international conferences. If the study protocol requires modifications, we plan to obtain IRB approval for amendments at each clinical trial stage.

## Discussion

This study leverages a primary care-based, prospective, multicenter, cluster-randomized, pragmatic trial design to offer a comprehensive and real-world evaluation of the effectiveness of the “WellCheck” digital healthcare application in managing T2D. Cutting-edge treatment options, specifically Envlo and Envlomet tablets allow for a targeted investigation of their glycemic lowering and metabolic efficacy. Furthermore, by encompassing a broad age range of participants (19–80 years) diagnosed with T2D, this study can markedly enhance the generalizability of its findings, ensuring that they are relevant to a diverse patient population.

Pragmatic trials study an intervention under the usual conditions of application and generally use routine care as the comparator, contrary to explanatory trials, testing an intervention under ideal conditions [36,37]. In cluster randomized trials, treatment interventions are allocated to clusters (i.e., groups of individuals) rather than to individuals. This is for manipulating the physical or social environment of the intervention when an individual intervention would likely result in contamination between the intervention and control participants at the group level [38]. Using cluster randomization to organize primary care centers into intervention and control groups according to specific characteristics further strengthens our methodology. This approach minimizes bias and enhances the reliability of the results, ensuring a well-structured comparison of outcomes across various healthcare settings.

However, the study design has certain limitations that warrant consideration. A major challenge of a cluster randomized design is potential latent confounding owing to the assignment of intervention and control groups to separate institutions [38]. The variations between clusters in implementation fidelity, participant demographics, and local practices can potentially affect the observed effectiveness of the intervention. Such disparities may introduce variability in outcomes, complicating the interpretation of an intervention’s true efficacy across different settings. To mitigate these limitations, we have utilized stratified randomization to balance institutional characteristics, and our statistical analyses will explicitly incorporate cluster-level random effects alongside patient-level fixed effects using robust statistical methods such as mixed-effects models and GEE.

Additionally, this study is conducted as a pragmatic clinical trial; thus, neither participants nor outcome assessors are blinded to intervention assignment. Due to the nature of the intervention—specifically, active physician-mediated feedback versus passive self-monitoring—implementing blinding is inherently challenging. Therefore, the possibility of bias associated with the lack of blinding should be considered when interpreting the study outcomes.

Finally, generalizing the results of this study to other diabetes medications besides enavogliflozin is limited. Our study specifically investigates a “digitally-integrated therapeutic approach” by explicitly combining the WellCheck digital healthcare application with enavogliflozin-based therapy. Thus, findings from this initial exploratory study may not be directly applicable to other SGLT2 inhibitors or diabetes treatments. Further research involving a broader range of medications is necessary to confirm whether the observed outcomes can be replicated with other pharmacological agents. Despite this limitation, focusing on a specific medication-digital integration provides valuable insights into optimizing digital healthcare applications in conjunction with pharmacotherapy, contributing to a nuanced understanding of managing T2D in diverse clinical contexts.

## Conclusion

In conclusion, this primary care-based, prospective, multicenter, cluster-randomized pragmatic trial evaluating the “WellCheck” digital healthcare application holds significant clinical utility and value in the management of type 2 diabetes. When validated, the findings of this study will contribute to the evidence base on the use of digital healthcare applications, potentially validating WellCheck as a valuable tool for improving glycemic control, reducing cardiovascular risk factors, and enhancing overall metabolic health in patients with type 2 diabetes. This research has the potential to inform clinical practice guidelines and policy decisions, ultimately improving the quality of care and health outcomes for individuals with type 2 diabetes.

## Supporting information

S1 File SPIRIT 2013 checklist. (DOCX)

S1 Table Questionnaire healthcare providers’ satisfaction with using WellCheck. (DOCX)

S2 Table Questionnaire particpants’ satisfaction with using WellCheck. (DOCX)

S1 Protocol Study protocol (original document). (DOCX)

S2 Protocol Study protocol (English translation). (DOCX)
