# Supplementary material for: Impact of the WellCheck smartphone app linked to electronic health records on clinical outcomes in patients with type 2 diabetes: Study protocol for primary care-based, prospective, multicenter, cluster-randomized, pragmatic clinical trials
Source: PLoS One. 2025 Aug 7;20(8):e0329003. doi: 10.1371/journal.pone.0329003 (PMC12331031; doi:10.1371/journal.pone.0329003)
Supplement: S1 Table — (DOCX) [file pone.0329003.s002.docx]

**S1 Table. Questionnaire on** **healthcare providers’ satisfaction with using WellCheck**

※ Has using WellCheck helped your healthcare team more effectively manage chronic conditions and improve efficiency?

|  | **Strongly Disagree** | **Disagree** | **Neutral** | **Agree** | **Strongly Agree** |
| --- | --- | --- | --- | --- | --- |
| **1.** Can you **better collect and verify information needed for treatment, such as a patient's blood sugar, lifestyle, and medical history**, using the WellCheck? |  |  |  |  |  |
| **2.** Has using WellCheck enabled your **office to provide your patients with better quality and more personalized care**? |  |  |  |  |  |
| **3.** Do patient education using WellCheck **help increase your patients' knowledge and understanding of healthcare, diabetes, and disease management**? |  |  |  |  |  |
| **4.** Do you feel that providing patient education through the WellCheck app has **helped patients take control of their healthcare at home**? |  |  |  |  |  |
| **5.** Do you feel that providing patient education and monitoring using the WellCheck has **motivated and encouraged patients** to better adhere to their health care? |  |  |  |  |  |
| **6.** Has the WellCheck app encouraged **patients to regularly measure and record their blood sugar or weight on their own**? |  |  |  |  |  |
| **7.** Has offering the WellCheck app **helped improve patient adherence and retention**? |  |  |  |  |  |
| **8.** Have you been able to **provide better quality care to your patients** using WellCheck? |  |  |  |  |  |
| **9. Do you anticipate** that using WellCheck to manage your patients will **increase rapport and satisfaction with your patients, resulting in higher visit retention**? |  |  |  |  |  |
| **10.** How likely are you to **recommend** WellCheck's patient management system **to other physicians**? |  |  |  |  |  |
