# Supplementary material for: Impact of the WellCheck smartphone app linked to electronic health records on clinical outcomes in patients with type 2 diabetes: Study protocol for primary care-based, prospective, multicenter, cluster-randomized, pragmatic clinical trials
Source: PLoS One. 2025 Aug 7;20(8):e0329003. doi: 10.1371/journal.pone.0329003 (PMC12331031; doi:10.1371/journal.pone.0329003)
Supplement: S2 Table — (DOCX) [file pone.0329003.s003.docx]

**S2 Table. Questionnaire on** **participants’ satisfaction with using WellCheck**

※ Did using WellCheck help improve your relationship with your healthcare professional, your satisfaction, and your experience with treatment?

|  | **Strongly Disagree** | **Disagree** | **Neutral** | **Agree** | **Strongly Agree** |
| --- | --- | --- | --- | --- | --- |
| **1.** Has the WellCheck app enabled you to **communicate better with your healthcare team about your blood sugar, weight management, and treatment**? |  |  |  |  |  |
| **2.** Does using the WellCheck app help your **healthcare provider at the hospital better understand your current health status**? |  |  |  |  |  |
| **3.** Did the **health information and education** provided by the hospital through the WellCheck app **increase your knowledge and understanding of your healthcare, diabetes, and disease management**? |  |  |  |  |  |
| **4.** Did the **health information and education** provided by the hospital through the WellCheck app **help you manage your own health at home**? |  |  |  |  |  |
| **5.** Do you think the **healthcare providers** using the WellCheck app **motivate and encourage you to stay on track with your healthcare**? |  |  |  |  |  |
| **6.** Have you been able to **measure and record your blood sugar or weight regularly on your own** after using the WellCheck app? |  |  |  |  |  |
| **7.** Has the WellCheck app **helped you stay on track with your medications**? |  |  |  |  |  |
| **8.** Do you think the WellCheck app has helped you receive **better quality care and treatment**? |  |  |  |  |  |
| **9.** Would you be **more likely to choose a healthcare provider who offers care, education, and blood sugar and weight management** using the WellCheck app? |  |  |  |  |  |
| **10.** How likely are you to **recommend a healthcare institution that provides care, education, and blood sugar and weight management** using the WellCheck app to **others**? |  |  |  |  |  |
