# Supplementary material for: Impact of the WellCheck smartphone app linked to electronic health records on clinical outcomes in patients with type 2 diabetes: Study protocol for primary care-based, prospective, multicenter, cluster-randomized, pragmatic clinical trials
Source: PLoS One. 2025 Aug 7;20(8):e0329003. doi: 10.1371/journal.pone.0329003 (PMC12331031; doi:10.1371/journal.pone.0329003)
Supplement: S1 Protocol — (DOCX) [file pone.0329003.s004.docx]

**임상연구계획서**

**2형 당뇨병 환자에서 전자의무기록 연동형 디지털 헬스케어 스마트폰 어플리케이션 '웰체크' 사용 시 연구자와의 연동 관리 여부가 임상경과에 미치는 효과를 확인하기 위한 일차의료기관 기반 전향적, 다기관, 군집 무작위배정, 실용 임상연구**

**A Primary Care-Based, Prospective, Multicenter, Cluster-Randomized, Pragmatic Clinical Trials to Determine the Effect of Linking data with Investigators When Using the Electronic Health Record-Linked Smartphone Application, ‘Well Check’, on Clinical Outcomes in Patients with Type 2 Diabetes Mellitus**

| **연구대상 소프트웨어:** | **디지털 헬스케어 스마트폰 어플리케이션 ‘웰체크’** |
| --- | --- |
| **연구계획서 번호:** | **DW_ODNENV_DB_01** |
| **연구계획서 버전:** | **V1.1** |
| **개발자:** | **㈜오디엔** |
| **의뢰자:** | **㈜대웅제약** |
| **연구계획서 작성일:** | **2024-05-16** |

**연구계획서 제·개정 이력**

| **No.** | **Version No.** | **Version Date** | **변경 내용** |
| --- | --- | --- | --- |
| 1 | 1.0 | 2024-05-02 | 해당사항 없음(제정) |
| 2 | 1.1 | 2024-05-16 | 무작위 배정 구체화 수정 |

**의뢰자**

| 회사명 | ㈜대웅제약 | | |
| --- | --- | --- | --- |
| 의뢰책임자 | 이창재, 박성수 | | |
| 주소 | 서울특별시 강남구 봉은사로 114길 12 | 우편번호 | 06170 |
| 연락처 | 02-550-8800 | | |

**연구계획서 요약**

| **연구계획서 번호** | DW_ODNENV_DB_01 |
| --- | --- |
| **임상연구 제목** | 2형 당뇨병 환자에서 전자의무기록 연동형 디지털 헬스케어 스마트폰 어플리케이션 '웰체크' 사용 시 연구자와의 연동 관리 여부가 임상경과에 미치는 효과를 확인하기 위한 일차의료기관 기반 전향적, 다기관, 군집 무작위배정, 실용 임상연구 |
| **연구계획서 버전** | V1.1 |
| **작성일** | 2024-05-16 |
| **단계 및 디자인** | 일차의료기관 기반 전향적, 다기관, 군집 무작위배정, 실용 임상연구 |
| **임상연구 실시기관** | 전국의 1차 의료기관 24 개소 |
| **임상연구 목적** | 실제 진료 환경에서 2형 당뇨병 환자를 대상으로 24주간 ‘웰체크’ 사용 시 연구자와의 연동 관리 여부에 따른 임상경과의 개선 효과 확인 |
| **의뢰자** | ㈜대웅제약 |
| **대상질환** | 2형 당뇨병 |
| **선정·제외기준** | **선정기준**  아래의 선정기준을 모두 만족하는 자를 선정한다.   - 만 19세 이상, 80세 이하의 성인 - 2형 당뇨병 환자로 허가사항에 근거하여 엔블로정 또는 엔블로멧서방정을 투여 중이거나 투여할 예정인 자 - 스마트폰을 이용하여 디지털 헬스케어 스마트폰 어플리케이션을 사용하는 데에 어려움이 없는 자 - 임상연구 참여기간 동안 혈당조절을 위해 개별적으로 적절한 운동과 식사요법을 병행할 예정인 자 - 연구 참여 기간 동안 적절한 피임법^*^에 따라 피임에 동의하거나, 임신할 계획이 없는 가임여성 및 남성   *호르몬성 피임제, 자궁 내 장치 또는 자궁 내 시스템의 이식, 정관수술, 난관 결찰, 이중 차단피임법(자궁 경부캡 또는 피임용 격막과 남성용 콘돔의 동시 사용 등) 등   - 연구대상자가 본 임상연구에 대하여 자세한 설명을 듣고 이해한 후, 자의로 임상연구 참여와 연구기간 동안 대상자 주의사항을 준수하기로 서면 동의한 자   **제외기준**  아래의 제외기준 중 어느 하나라도 해당되는 자는 본 임상연구에서 제외한다.     - 2형 당뇨병 이외의 당뇨병(1형 당뇨병, 당뇨병성 케톤산증, 임신성 당뇨병 등)인 자 - 엔블로정 또는 엔블로멧 서방정의 허가사항에 따라 투여 금기에 해당하는 자 - 엔블로정 또는 엔블로멧서방정의 구성성분에 과민반응 및 그 병력이 있는 환자 - 사구체여과율(eGFR, estimated Glomerular Filtration Rate) 30 mL/min/1.73m^2^ 미만인 환자 - 사구체여과율(eGFR, estimated Glomerular Filtration Rate) 60 mL/min/1.73m^2^ 미만의 신장애 환자, 말기 신질환 또는 투석중인 환자 - 중등증 및 중증의 간장애 환자(AST 또는 ALT > 정상 상한치의 3배, Total Bilirubin > 정상 상한치의 2배, 간염 또는 간부전) - 뉴욕심장학회(New York heart association, NYHA)의 분류에 의한 class III 혹은 IV인 자 - 등록 시점 3개월 이내부터 비만치료제 또는 체중 감량 약물을 사용한 치료 또는 기타 치료(수술, 식이요법 등)으로 인해 체중이 불안정한 환자 - 심신미약자 - 임부 및 수유부 - 다른 임상시험에 참여하여 임상시험용의약품 또는 임상시험용의료기기를 투여(적용)중인 자 - 기타 연구자(담당의사)의 판단에 따라 본 임상연구에 참여하는 것이 적합하지 않은 것으로 판단된 자 |
| **목표 연구대상자 수** | 약 480명 (통계학적 유의성 확보를 위한 최소 인원수에 중도 탈락자 약 20%를 고려) |
| **임상연구 기간** | - 예상 전체 연구기간: 첫 IRB 승인일 ~ 2026년 12월 31일   (단, 연구대상자 등록률에 따라 변경될 수 있음)   - 대상자별 임상연구 기간: 약 24주(6개월) |
| **연구대상소프트웨어** | 디지털 헬스케어 스마트폰 어플리케이션 ‘웰체크’ |
| **임상연구 설계 및 방법** | 본 임상연구는 1차 의료기관의 실제 진료환경에서 2형 당뇨병 환자를 대상으로 24주간 ‘웰체크’ 사용 시 연구자와의 연동 관리 여부에 따른 혈당, 혈압, 체중의 개선 효과를 확인하기 위한 일차의료기관 기반 전향적, 다기관, 군집 무작위배정, 실용 임상연구로 설계되었다.  실제 진료환경에서 연구자(담당의사)의 의학적 판단에 따라 2형 당뇨병 환자 중 스마트폰을 이용한 디지털헬스케어 모바일 어플리케이션을 사용하는 데에 어려움이 없는 환자를 모집한다.  ‘웰체크’의 임상적 근거를 확인하기 위해 전향적, 다기관 임상연구를 진행하되, 기관에서 시험군과 대조군을 명확히 구분하여 중재를 수행하기 어려운 일차의료기관의 현실적 상황을 반영하여 1:1군집 무작위배정을 통해 시험군(연구자(담당의사) 연동 관리 ‘웰체크’ 사용군)을 진료하는 기관과 대조군(단순 자가 관리 ‘웰체크’ 사용군)을 진료하는 기관으로 의료기관을 구분한 후 연구를 수행 한다.  본 임상연구는 실제 진료환경에서 수행되는 인구학적 정보, 신체계측, 활력징후, 검사실 검사 등의 정보를 ‘웰체크’ 사용 시작 후 최대 24주까지 수집한다. 자료는 실제 진료환경에서 기록되는 의무기록 및 ‘웰체크’를 통해 수집한 자료를 근거로 수집하며, 본 임상연구에서 의무적으로 규정된 방문 및 검사 또는 처치는 없다.  그러나 진료환경에 따라 연구대상자 등록일(베이스라인, 0일) 로부터 6주(±2주), 12주(±2주), 18주(±2주), 24주(±2주) 시점에 전향적으로 유효성 및 안전성 평가 자료를 수집하는 추적방문이 진행될 예정이며, 수집 항목은 연령, 성별, 과거 및 현재 병력, 생활습관(흡연, 음주, 운동량), 선행 및 병용 약제 등의 기본 임상적 정보, 그리고 체중, 체질량지수, 체지방량, (체성분분석기, optional), 공복혈당, 당화혈색소, 혈압(수축기/이완기), 총콜레스테롤, LDL 콜레스테롤, HDL 콜레스테롤, 중성지방, 간기능 검사(AST, ALT, γ-GTP) 등의 임상 지표, 안전성 평가 항목으로 활력징후, 검사실 검사 등 이다. 이때 연구자는 일상 진료 과정에서 수집된 자료를 근거하여 연구에 필요한 자료를 수집한다.  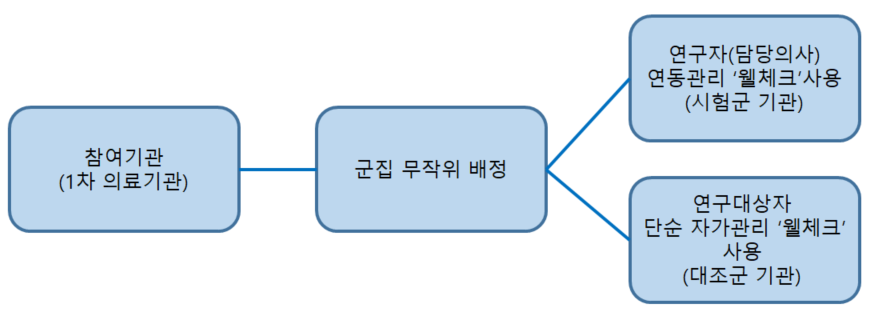<임상연구 설계>  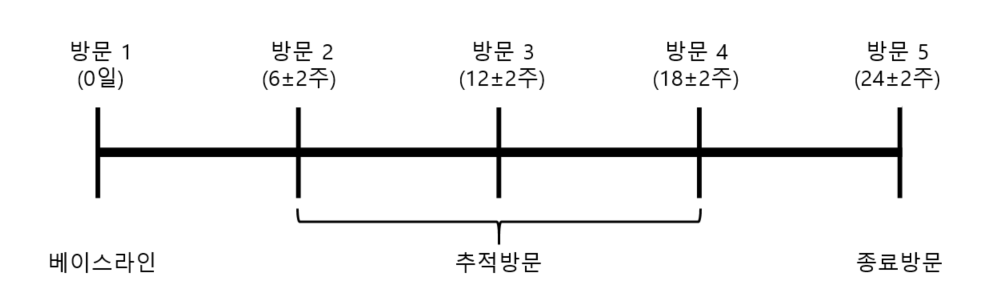  <임상연구 흐름도> |
| **유효성 평가변수** | **1차 유효성 평가변수**   1. 베이스라인 대비 24주 시점의 HbA1c변화율 및 변화량   **2차 유효성 평가변수**   1. 베이스라인 대비 6, 12, 18, 24주 시점의 FPG 변화율 및 변화량 2. 베이스라인 대비 6, 12, 18주 시점의 HbA1c 변화율 및 변화량 3. 베이스라인 대비 6, 12, 18, 24주 시점의 HbA1c <7% 달성 대상자 비율 4. 베이스라인 대비 6, 12, 18, 24주 시점의 HbA1c <6.5% 달성 대상자 비율 5. 베이스라인 대비 12, 24주 시점의 치료적 반응[베이스라인 대비 각 평가 시점에서 HbA1c 변화량(베이스라인의 HbA1c–각 평가 시점의 HbA1c)> 0.5 % 또는 HbA1c < 7 %] 달성 대상자 비율 6. 베이스라인 대비 6, 12, 18, 24주 시점의 혈압(수축기혈압, 이완기혈압) 변화량 7. 베이스라인 대비 6, 12, 18, 24주 시점의 체질량지수(BMI) 및 체중 변화량 8. 베이스라인 대비 6, 12, 18, 24주 시점의 5% 이상 체중 및 BMI 감소 대상자 비율   **탐색적 평가변수**   1. 베이스라인 대비 6, 12, 18 24주 시점의 지질농도(Total cholesterol, LDL-C, HDL-C, Triglyceride)의 변화량 2. 베이스라인 대비 6, 12, 18, 24주 시점의 간기능 관련 지표(AST, ALT, γ-GTP)의 변화량 3. 베이스라인 대비 6, 12, 18, 24주 시점의 신기능 관련 지표[e-GFR, UACR(Urine Albumin to Creatinine Ratio), UGCR(Urine Glucose to Creatinine Ratio)] 변화량 4. 베이스라인 대비 12, 24주 시점의 체성분분석계 지표(골격근량, 체지방량, 체지방률, 근육량, 허리/엉덩이 둘레, 체수분, 세포내수분, 세포외수분, 세포외수분비, 복부지방률 등) 변화량 5. 베이스라인 대비 12, 24주 시점의 심혈관질환 합병증 위험도(ASCVD risk) 변화량 |
| **안전성 평가변수** | 1. 검사실 검사, 활력징후, 신체검사 결과 |
| **통계분석 방법** | **일반적 원칙**  연속형 변수는 기술통계량(대상자의 수, 평균, 표준편차, 중앙값, 최소값, 최대값)을 제시하고, 범주형 변수는 빈도와 백분율을 제시한다. 특별한 명시가 없는 경우 모든 검정은 유의수준 5% 하에서 양측검정을 원칙으로 하며, 모든 p-value에 대해서는 소수점 최대 3자리까지 제시하고, 이외의 경우 소수점 두번째 자리까지 나누어 떨어지지 않을 경우, 소수점 세번째 자리에서 반올림하여 소수점 2자리까지 제시한다.  **유효성 평가변수**  1차 및 2차 유효성 평가변수에 대하여 베이스라인, 6주, 12주, 18주, 24주 시점에 기술통계량을 제시하고, 베이스라인 대비 24주 시점 혹은 6주, 12주, 18주 시점의 차이에 대하여 변화량에 대한 Independent sample t-test, Mann Whitney U test, 또는 Wilcoxon sum rank test를 시행하여 분석한다.  **탐색적 평가변수**  탐색적 평가변수에 대하여 베이스라인, 6주, 12주, 18주, 24주 시점에 기술통계량을 제시하고, 베이스라인 대비 24주 시점 혹은 6주, 18주 시점의 차이에 대하여 변화량에 대한 Independent sample t-test, Mann Whitney U test, 또는 Wilcoxon sum rank test를 시행하여 분석한다. |

**임상연구 자료수집 일정표**

| **수집 일정**  **수집 항목** | **베이스라인** | **추적방문 (선택, ☎)** | **추적방문** | **추적방문 (선택, ☎)** | **종료방문** |
| --- | --- | --- | --- | --- | --- |
|  | 방문1 | 방문2 | 방문3 | 방문4 | 방문5 |
|  | 0일 | 6주(±2주) | 12주(±2주) | 18주(±2주) | 24주(±2주) |
| 서면동의 취득^1)^ | ○ |  |  |  |  |
| 선정/제외기준 확인 | ○ |  |  |  |  |
| 무작위배정 번호 부여 | ○ |  |  |  |  |
| 웰체크 설치 및 기관 연동 | ○ |  |  |  |  |
| 인구학적 정보^2)^ | ○ |  |  |  |  |
| 2형 당뇨병 정보^3)^ | ○ |  |  |  |  |
| 병력 조사^4)^ | ○ |  |  |  |  |
| 음주/흡연/생활습관^5)^ | ○ | (○) | ○ | (○) | ○ |
| 신체계측^6)^ | ○ | (○) | ○ | (○) | ○ |
| 활력징후^7)^ | ○ | (○) | ○ | (○) | ○ |
| 체성분분석계(Inbody) 검사^8)^ | (○) | (○) | (○) | (○) | (○) |
| 선행/병용약물^9)^ | ○ | (○) | ○ | (○) | ○ |
| 검사실 검사^10)^ | ○ | (○) | ○ | (○) | ○ |
| 웰체크 사용 빈도 (접속일 수) |  |  |  |  | ○ |
| 웰체크 사용 경험 만족도 설문^11)^ |  |  |  |  | ○ |

*본 임상연구를 위한 추가적인 방문 및 실시되는 검사실 검사는 없다. 일상적인 진료환경에서 방문1(베이스라인, 0일) 이후 24주까지 6주 간격(방문2: 6주, 방문3: 12주, 방문4: 18주, 방문5: 24주)의 자료를 수집 하는 것을 권장하며, 방문 2(6주)와 방문 4(18주)는 연구자(담당의사)의 판단에 따라 선택 방문 및 유선을 통한 자료 수집이 가능하고, 연구 중 중지/탈락 된 경우 해당 시점까지 가능한 최대한으로 수집된 자료를 증례기록서에 기록한다.

1. 임상연구 절차를 수행하기 전에 서면동의서를 취득하여야 한다. 서면동의일과 방문1(베이스라인, 0일)은 상이할 수 있으나, 임상연구 참여 전 반드시 동의 취득이 선행되어야 한다.
2. 인구학적 정보[이니셜, 성별, 연령(생년월), 임신 여부 및 수유 여부]를 수집한다.
3. 2형 당뇨병 정보(진단일)를 수집한다.
4. 병력은 방문 1(베이스라인, 0일) 이전 6개월 이내의 병력을 수집한다.
5. 음주/흡연/생활습관은 매 방문 시, 시험군의 경우 ‘웰체크’ 내 ‘내원 전 문진’ 기능 또는 진료시 연구자(담당의사)의 문진으로 수집하며, 대조군의 경우 진료 시 연구자(담당의사)의 문진으로 수집한다. 음주력은 주당 음주 횟수, 1회당 음주량에 대한 정보를 포함한다. 흡연력은 현재 흡연, 금연, 비흡연 중 한 가지를 선택한다. 생활습관은 식습관, 운동습관을 포함하며, 식습관 항목에는 불규칙한 식사, 과식, 탄수화물/당 과다섭취, 지방 과다섭취, 염분 과다섭취 항목 중 복수 선택 가능하며, 운동습관 항목에는 주당 운동횟수, 운동종류(걷기, 유산소운동, 근력운동), 운동강도(30분 이내, 1시간 이내, 장시간)를 포함한다.
6. 신체계측(신장, 체중, 체질량지수) 정보를 수집하며, 체질량지수는 신장 및 체중을 통해 EDC 자동계산으로 계산된다. 신장은 방문1(베이스라인, 0일) 시점에만 수집하며, 소수점 첫째 자리까지 수집한다.
7. 최소 5분 이상의 휴식을 취한 후 활력징후(수축기 혈압, 이완기 혈압, 맥박) 정보를 수집한다. 매 방문마다 측정 검사 결과가 있는 경우, 결과를 수집한다.
8. 체성분분석계(골격근량, 체지방량, 체지방률, 근육량, 허리/엉덩이 둘레, 체수분, 세포내수분, 세포외수분, 세포외수분비, 복부지방률 등) 검사는 방문1(베이스라인, 0일), 방문3(추적방문, 12주) 및 방문5(종료방문, 24주)에서 수집한다. 이 변수는 탐색적 임상변수로 측정 가능한 기관에서만 시행한다.
9. - 선행약물: 방문1(베이스라인, 0일) 기준 4주 이내에 2형 당뇨병 치료 목적의 당뇨병치료제에 한하여 약물명(성분명 또는 상품명), 투여 기간(투여 시작일, 투여 종료일, 지속 여부), 1회 투여 용량/단위, 1일 투여 횟수, 투여 경로 정보를 수집한다.

- 병용약물: 방문1(베이스라인, 0일) 이후 연구 기간 동안 3개월 이상 꾸준히 복용하는 2형 당뇨병 치료 목적 약물 약물을 포함한 모든 병용약물(성분명 또는 상품명), 투여 사유, 투여 기간(투여 시작일, 투여 종료일, 지속 여부), 1회 투여 용량/단위, 1일 투여 횟수, 투여 경로, 투여 용량 변경 또는 투여 중단 여부 및 사유) 정보를 수집한다.

1. 검사실 검사는 실제 진료환경에서 통상적인 진료 방법에 따라 진행한 경우 수집하며, 방문1(베이스라인, 0일) 기준으로 4주 이내 HbA1c 결과 및 그 외 3개월 이내 수행한 검사 결과가 있는 경우, 해당 검사 결과로 방문1(베이스라인, 0일) 검사실 검사를 대체할 수 있다. 심혈관질환 합병증 위험도(ASCVD risk)는 ASCVD 2013 Risk Calculator from AHA/ACC 공식을 따르며, eCRF 내 수집된 정보(성별, 연령, 수축기 혈압, 총 콜레스테롤, HDL-C, 고혈압 약물치료 여부, 당뇨병 진단 여부, 흡연 여부 항목)를 토대로 연구 종료 후 분석을 시행할 예정이며, 시험군은 웰체크 내 자동 계산 기능으로 연구자(담당의사)가 콜레스테롤, 혈압 값을 입력하여 대상자 상담 시 사용이 가능하다.

| 주요 검사실 검사 | HbA1c, FPG(FBS) |
| --- | --- |
| 기타 검사실 검사 (혈액 검사) | Total Cholesterol, HDL-C, LDL-C, Triglyceride, ALT, AST, γ-GTP, Creatinine, e GFR, UACR, UGCR |

1. 종료방문 시 의료진(담당의사) 및 대상자의 웰체크 사용 경험에 대한 설문조사를 서면으로 시행한다.

**목차**

[1. 서론 14](#_Toc165628317)

[1.1 연구의 배경 및 이론적 근거 14](#_Toc165628318)

[2. 임상연구의 목적 17](#_Toc165628319)

[2.1 1차 목적 17](#_Toc165628320)

[2.2 2차 목적 17](#_Toc165628321)

[2.3 탐색적 목적(탐색적 평가) 17](#_Toc165628322)

[2.4 안전성 평가 18](#_Toc165628323)

[3. 연구대상자의 선정 19](#_Toc165628324)

[3.1 연구 대상자 수 19](#_Toc165628325)

[3.2 산출근거 19](#_Toc165628326)

[3.3 선정기준 20](#_Toc165628327)

[3.4 제외기준 20](#_Toc165628328)

[3.5 연구 완료 및 중지/탈락 21](#_Toc165628329)

[3.6 임상연구의 중단 22](#_Toc165628330)

[4. 임상연구 방법 23](#_Toc165628331)

[4.1 전반적인 임상연구 디자인 23](#_Toc165628332)

[4.2 임상연구 기간 24](#_Toc165628333)

[4.3 임상연구의 진행 및 일정 24](#_Toc165628334)

[방문 일정 24](#_Toc165628335)

[4.4 수집정보 및 항목 25](#_Toc165628336)

[4.5 무작위 배정 30](#_Toc165628337)

[5. 연구대상 소프트웨어 32](#_Toc165628338)

[5.1 연구대상 소프트웨어의 정보 32](#_Toc165628339)

[5.2 연구대상 소프트웨어의 사용 34](#_Toc165628340)

[6. 자료 관리 35](#_Toc165628341)

[6.1 근거 문서 35](#_Toc165628342)

[6.2 자료 입력 35](#_Toc165628343)

[6.3 자료 확인 36](#_Toc165628344)

[6.4 자료 보관 36](#_Toc165628345)

[6.5 자료 열람 36](#_Toc165628346)

[7. 평가기준 및 평가방법과 통계분석 방법 37](#_Toc165628347)

[7.1 평가변수 37](#_Toc165628348)

[7.2 통계분석 방법 38](#_Toc165628349)

[유효성 평가 대상자군 38](#_Toc165628350)

[안전성 평가 대상자군 38](#_Toc165628351)

[1차 유효성 평가 변수 38](#_Toc165628352)

[2차 유효성 평가 변수 38](#_Toc165628353)

[탐색적 평가 변수 39](#_Toc165628354)

[활력징후 39](#_Toc165628355)

[검사실 검사 39](#_Toc165628356)

[8. 윤리적 고려사항 및 행정적 절차 40](#_Toc165628357)

[8.1 공용기관생명윤리위원회/ 임상시험심사위원회(IRB) 40](#_Toc165628358)

[8.2 윤리적 고려사항 40](#_Toc165628359)

[8.3 품질 보증 및 점검 40](#_Toc165628360)

[8.4 연구대상자 동의 41](#_Toc165628361)

[8.5 연구계획서 승인 및 변경 41](#_Toc165628362)

[8.6 임상연구 실시기관 모니터링 41](#_Toc165628363)

[8.7 기밀 유지 및 연구대상자의 비밀 보장 42](#_Toc165628364)

[8.8 연구대상자 안전보호에 관한 대책 42](#_Toc165628365)

[8.9 임상연구 결과의 이용 및 발표 43](#_Toc165628366)

[9. 연구 책임자 44](#_Toc165628367)

[9.1 연구 총괄 책임자 정보 44](#_Toc165628368)

[9.2 연구 총괄 책임자의 역할 및 책임 44](#_Toc165628369)

[10. 별첨 45](#_Toc165628370)

[11. 참고문헌 46](#_Toc165628371)

**약어 목록**

| ALT | Alanine Aminotransferase | 알라닌아미노전달효소 |
| --- | --- | --- |
| ASCVD | Atherosclerotic cardiovascular disease | 죽상경화성 심혈관질환 |
| AST | Aspartate Aminotransferase | 아스파르테이트아미노전달효소 |
| BMI | Body Mass Index | 체질량지수 |
| CFR | Code of Federal Regulations | 미국연방정부규정 |
| CS | Clinically Significant | 임상적으로 의미 있는 비정상 |
| DPP-4 | dipeptidyl peptidase 4 | 다이펩타이드분해효소 |
| eCRF | electronic Case Report Form | 전자증례기록서 |
| EDC | Electronic Data Capture | 전자 데이터 수집 |
| FPG | Fasting Plasma Glucose | 공복혈당 |
| GCP | Good Clinical Practice | 의약품 임상시험 관리기준 |
| GLP-1 | Glucagon-like Peptide-1 | 글루카곤양 펩티드-1 |
| γ-GTP | Gamma glutamyl transferase | 감마글루타밀전이효소 |
| HbA1c | Hemoglobin A_1c_ | 헤모글로빈 A1c |
| HDL-C | High Density Lipoprotein Cholesterol | 고밀도 지질단백질 |
| ICH | International Council for Harmonisation | 국제의약품규제조화위원회 |
| IEC | Independent Ethics Committee | 독립된 윤리위원회 |
| IRB | Institutional Review Board | 기관생명윤리위원회 |
| LDL-C | Low Density Lipoprotein Cholesterol | 저밀도 지질단백질 |
| NCS | Not Clinically Significant | 임상적으로 의미 없는 비정상 |
| SE | Standard Error | 표준 오차 |
| SOP | Standard Operating Procedures | 표준작업지침서 |
| TC | Total Cholesterol | 총콜레스테롤 |
| TG | Triglyceride | 트리글리세리드 |
| UACR | Urine Albumin to Creatinine Ratio | 소변 알부민-크레아티닌 비율 |
| UGCR | Urine Glucose to Creatinine Ratio | 소변 포도당-크레아티닌 비율 |
|  |  |  |
|  |  |  |
|  |  |  |

# 서론

## 연구의 배경 및 이론적 근거

당뇨병, 비만, 이상지질혈증은 중요한 대사성 질환이며, 심장마비, 뇌졸중, 고혈압과 같은 심혈관 질환과 관련된 중요한 위험요소이다. 특히 당뇨병은 유전적, 환경적 원인에 의해 인슐린의 기능에 이상이 생겨 혈액 속의 포도당이 세포로 전달/저장되지 못하고 혈액 중에 지나치게 많아져 혈당의 수치가 정상인보다 높아지는 고혈당증(Hyperglycemia) 증상을 보이는 심각한 대사성 질환이다. 세계 당뇨병 재단(International Diabetes Federation, IDF)에 따르면 전세계 당뇨병 환자 수는 2017년 4억2천5백만명에서 2019년 4억6천3백만명으로 증가하였으며, 2040년에는 전체 성인의 약 10% 이상인 6억4천2백만명으로 폭발적인 증가를 보일 것으로 예상된다 [1]. 국내에서도 경제성장과 소득증가로 인한 식습관의 서구화, 운동부족, 수명연장 등의 복합적인 요인으로 당뇨병 환자가 급증세를 보이고 있으며, 2007년 300만명에서 2010년 351만명으로 늘어났고, 2030년에는 540만명(통계청, 전 인구의 10.85%)으로 늘어날 것으로 추산된다.

당뇨병은 크게 1형 당뇨병(Type 1 Diabetes:T1D), 2형 당뇨병(Type 2 Diabetes:T2D), 임신성 당뇨병(Gestational Diabetes)으로 분류된다. 이 중에서 2형 당뇨병은 전세계 당뇨병의 90% 이상을 차지하고 있는 가장 흔한 유형의 당뇨병으로, 초기에는 몸의 세포가 인슐린에 완전히 반응하지 못하여 발생하나, 인슐린 저항성이 시작되며 부적절한 시기에 인슐린 생산 증가가 촉진된다 [2]. 질병이 진행됨에 따라 불충분한 인슐린 생성은 췌장 베타 세포가 수요를 따라가지 못하는 결과로 발생할 수 있다.

일반적으로 처음 2형 당뇨병을 진단받은 환자에게서는 우선 적극적인 생활습관개선을 하고, 이것만으로 당화혈색소 목표에 도달하지 못한 경우에 약제치료를 시작한다 [3]. 기존 경구용 당뇨병 치료제는 크게 인슐린제제, 설폰우레아(Sulfonylurea, SU)계열 약물, 티아졸리딘디온(Thiazolidinedione,TZD)계열 약물, 비구아니드(biguanide)계열 약물, α-Glucosidase 억제제, 메티글리티나이드계, 인크레틴 유사체, DPP-4(Dipeptidyl Peptidase-4)억제제 등으로 구분할 수 있다.

당뇨병의 치료는 일반적으로 미국 당뇨병 학회(American Diabetes Association, ADA)의 가이드라인을 참고하여 심혈관질환 여부 및 당뇨병 진행 정도에 따라 단독 또는 병용 투여 요법이 사용된다. 미국당뇨병학회에서는 현재 대부분의 당뇨병 성인에게 당화혈색소(HbA1c) 수치 7.0% 미만, 혈압 140/90 mmHg 미만(심혈관 질환 위험이 높은 환자의 경우 130/90 mmHg 미만), 저밀도 지단백 콜레스테롤(LDL-C) 100 mg/dL 미만을 달성할 것을 권장하고 있다 [4]. 또한, 국내 당뇨병 치료지침에 따르면 2형 당뇨병 환자에서 일반적인 혈당조절 목표는 HbA1c 6.5% 미만이다.

2형 당뇨병 환자를 대상으로 시행한 전향적 연구인 United Kingdom Prospective Diabetes Study (UKPDS) 결과에 의하면 2형 당뇨병 환자에서 당화혈색소(HbA1c) 수치를 1% 감소시키면 미세혈관합병증은 37%, 심근경색은 14% 감소시키는 것으로 보고되었으며, 연구마다 약간의 차이는 있으나 적극적이고 엄격한 혈당조절은 당뇨병 합병증 발생을 예방할 수 있으며, 이미 발생된 합병증의 진행속도를 늦출 수 있는 가장 효과적인 방법으로 보고되었다.

뿐만 아니라, 2형 당뇨병 진단과 동시에 모든 환자는 생활습관개선을 위한 교육을 실시하고 실천이 필요하다. 당뇨병 자기관리교육의 목표는 당뇨병환자가 혈당조절을 포함해 효과적으로 당뇨병을 관리하여 당뇨병합병증과 사망률 등 예후를 개선함으로써 궁극적으로 환자의 삶의 질을 높이는 데 있으며, 당뇨병 자기관리는 환자가 당뇨병 관리에 필요한 지식과 방법을 습득하여 건강한 식사계획을 세우고 정기적인 신체활동을 하며 효과적으로 자기관리 행동변화를 할 수 있게 하고 다양한 상황에 대처할 수 있도록 돕는다. 2형 당뇨병 환자를 대상으로 한 무작위대조 임상연구에서 자기관리 및 교육은 건강과 관련된 문제 발생 시 그 대처능력을 향상시켰고 자기관리 행동을 개선시켰으며, 자기효능감 및 임파워먼트(empowerment)가 강화되었고, 당뇨병 관리의 질이 향상되었다. 당뇨병 치료목표의 달성을 위해서는 환자의 효과적인 자기관리 및 정신적 안녕이 기반되어야 한다.

당뇨병 환자들은 당뇨병을 처음 진단받았을 때 자기관리교육을 통하여 효과적인 자기관리를 시작할 수 있어야 한다. 당뇨병은 지속적 관리가 필요한 만성질환이므로, 환자의 상황이 변화될 때 마다 자기관리교육을 보완하고 지속시켜야 하며, 건강한 생활습관의 지속을 위해서는 최소 6개월의 관리가 필요하며, 메타분석 결과 6-12개월에 걸쳐 10시간 이상 시행된 자기관리 교육은 혈당개선과 사망률 감소 효과를 보였다. 따라서 당뇨병 자기관리 및 교육은 치료목표에 도달하지 못했을 때나, 생애주기의 변화 또는 합병증 발생 등 환자상태에 따라, 나이 및 개인의 요구와 취향을 반영한 환자 위주의 교육이 효과적이다.

위와 같이 상호작용적, 지속적인 과정으로 정의되는 당뇨병 관련 인성 교육 및 지원을 통해 당뇨병에 대한 중재를 성공적으로 자가 관리하는 데 필요한 지식, 기술 및 능력을 개발하는 것은 성공적인 것으로 입증되었고 [4], 기술이 발달함에 따라 휴대폰 메시지, 모바일 애플리케이션, 웹기반 알고리듬 등 다양한 방식의 디지털 건강코칭이 혈당을 개선시키고 당뇨병환자의 자기관리능력을 향상시킬 수 있다는 연구결과들이 보고되고 있으므로 교육 또는 상담과 결합된 자가 모니터링은 고혈압 환자의 혈압 관리 및 약물 순응도를 개선하는 데 도움이 될 수 있다 [5].

전 세계적으로 의료 분야에서 정보 및 통신 기술의 사용이 빠르게 확대되고 있다. [6]. 디지털 의료 기술은 검증된 의료 서비스와 환자 자가 관리 사이의 격차를 해소함으로써 접근성, 효과성, 삶의 질을 향상시키는 방법으로 인정받고 있다 [7]. 그럼에도 불구하고 그 결과는 일관되지 않았다. 두 건의 리뷰에 따르면 원격 의료가 혈압을 효과적으로 낮추는 것으로 나타난 반면, 다른 리뷰에서는 이러한 결과가 일관되게 입증되지 않았다 [8,9]. 디지털 헬스케어 스마트폰 어플리케이션은 당뇨병, 고혈압과 같은 만성 질환 관리에 널리 사용되어 왔으며, 의료 서비스 제공 향상, 환자 참여도 증가, 자가 관리 개선에 대한 유망한 전망을 제공한다. 그러나 이러한 어플리케이션을 병원 시스템 내에 통합하여 이러한 질환을 관리하는 데 미치는 영향에 대해서는 아직 결정적인 증거가 부족하다 [10].

이에, 본 임상연구를 통하여 2형 당뇨병 성인 환자를 대상으로, 일상 진료 하에서 혈당을 낮추고, 혈압과 체중을 조절하기 위해 병원 연계 디지털 헬스케어 스마트폰 어플리케이션 ‘웰체크(Well Check)’을 사용하는 것의 실제 효과를 확인하고, 기타 유효성 및 안전성 정보를 평가하여 실제 사용 자료를 확보 하는 것을 목표로 하였다.

# 임상연구의 목적

본 임상연구는 실제 진료 환경에서 2형 당뇨병 환자를 대상으로 24주간 ‘웰체크’ 사용 시 연구자와의 연동 관리 여부에 따른 임상경과의 개선 효과의 확인을 목적으로 한다.

## 1차 목적

베이스라인 이후 24주 시점의 HbA1c 변화율 및 변화량을 평가한다.

## 2차 목적

1. 베이스라인 이후 6, 12, 18, 24주 시점의 FPG 변화율 및 변화량
2. 베이스라인 이후 6, 12, 18주 시점의 HbA1c 변화율 및 변화량
3. 베이스라인 이후 6, 12, 18, 24주 시점의 HbA1c <7% 달성 대상자 비율
4. 베이스라인 이후 6, 12, 18, 24주 시점의 HbA1c <6.5% 달성 대상자 비율
5. 베이스라인 대비 12, 24주 시점의 치료적 반응[베이스라인 대비 각 평가 시점에서 HbA1c 변화량(베이스라인의 HbA1c–각 평가 시점의 HbA1c)> 0.5 % 또는 HbA1c < 7 %] 달성 대상자 비율
6. 베이스라인 이후 6, 12, 18, 24주 시점의 혈압(수축기혈압, 이완기혈압) 변화량
7. 베이스라인 이후 6, 12, 18, 24주 시점의 체중 및 체질량지수(BMI) 변화량
8. 베이스라인 이후 6, 12, 18, 24주 시점의 5% 이상 체중 및 BMI 감소 대상자 비율

## 탐색적 목적(탐색적 평가)

1. 베이스라인 이후 6, 12, 18, 24주 시점의 지질농도(Total cholesterol, LDL-C, HDL-C, Triglyceride)의 변화량
2. 베이스라인 이후 6, 12, 18, 24주 시점의 간기능 관련 지표(AST, ALT, γ-GTP)의 변화량
3. 베이스라인 이후 6, 12, 18, 24주 시점의 신기능 관련 지표[e-CFR, UACR(Urine Albumin to Creatinine Ratio), UGCR(Urine Glucose to Creatinine Ratio)] 변화량
4. 베이스라인 이후 12, 24주 시점의 체성분분석계 지표(골격근량, 체지방량, 체지방률, 근육량, 허리/엉덩이 둘레, 체수분, 세포내수분, 세포외수분, 세포외수분비, 복부지방률 등) 변화량
5. 베이스라인 이후 12, 24주 시점의 심혈관질환 합병증 위험도(ASCVD risk) 변화량

## 안전성 평가

1. 검사실 검사, 활력징후, 신체검사 결과

# 연구대상자의 선정

## 연구 대상자 수

최소 480명 (통계학적 유의성 확보를 위한 최소 인원수, 중도탈락 약 20% 고려)

## 산출근거

Pilot study 결과를 참조하여 2형 당뇨병 환자에서 웰체크 사용 24주 후 대조군 대비 평균 당화혈색소의 차이를 0.4% (8.0% vs. 7.6%), 표준편차(*s*)를 1.1%로 간주하였다. 이 결과를 참고하여 G*Power 3.1.9.7을 이용하여 연구대상자수(*n*)를 계산할 때 필요한 총 샘플 사이즈는 약 274명으로 추산된다.

본 연구는 군집 무작위 배정을 사용하므로, 동일 의료기관 내 환자간의 잠재적 군집 내 상관관계를 고려하기 위해 설계 효과(design effect)를 통합하여 샘플 사이즈 계산을 추가로 조정하였다. 설계 효과는 다음 표준 공식을 사용하여 계산하였다.

$$Design effect=1+\left( m-1 \right)\times\mathrm{ICC}$$

제2형 당뇨병 환자를 대상으로 1차 진료 환경에서 수행된 선행연구[11, 12]를 참고하여, 보수적으로 군집 내 상관계수(intraclass correlation coefficient, ICC) 값을 0.02로 채택하고, 의료기관당 평균 군집 크기(m)를 20명으로 가정하였다. 따라서 설계 효과는 1.38로 계산되었으며, 이러한 조정을 초기 연구대상자수 추정치에 적용하면 수정된 총 샘플 사이즈는 378명으로 추산된다. 마지막으로, Drop-out rate를 약 20%로 상정하여 약 480명의 연구대상자를 모집하고자 한다.


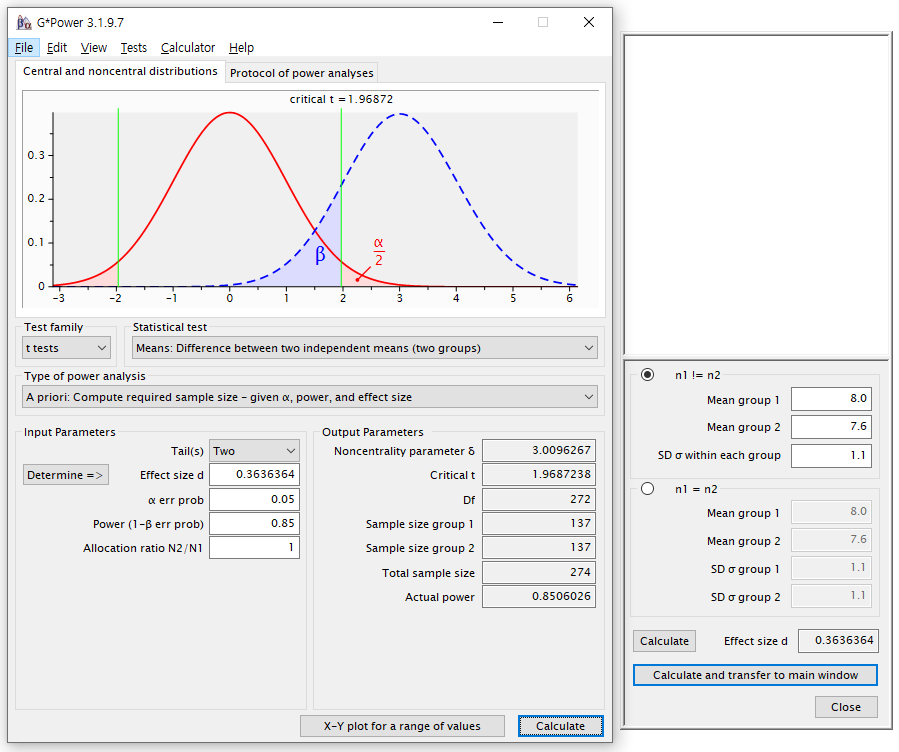


<연구대상자 수 산출 – 당화혈색소 기준>

## 선정기준

아래의 선정기준을 모두 만족하는 자를 선정한다.

1. 만 19세 이상, 80세 이하의 성인
2. 2형 당뇨병 환자로 허가사항에 근거하여 엔블로정 또는 엔블로멧서방정을 투여 중이거나 투여할 예정인 자
3. 스마트폰을 이용하여 디지털 헬스케어 스마트폰 어플리케이션을 사용하는 데에 어려움이 없는 자
4. 임상연구 참여기간 동안 혈당조절을 위해 개별적으로 적절한 운동과 식사요법을 병행할 예정인 자
5. 연구 참여 기간 동안 적절한 피임법^*^에 따라 피임에 동의하거나, 임신할 계획이 없는 가임여성 및 남성

*호르몬성 피임제, 자궁 내 장치 또는 자궁 내 시스템의 이식, 정관수술, 난관 결찰, 이중 차단피임법(자궁 경부캡 또는 피임용 격막과 남성용 콘돔의 동시 사용 등) 등

1. 연구대상자가 본 임상연구에 대하여 자세한 설명을 듣고 이해한 후, 자의로 임상연구 참여와 연구기간 동안 대상자 주의사항을 준수하기로 서면 동의한 자

## 제외기준

아래의 제외기준 중 어느 하나라도 해당되는 자는 본 임상연구에서 제외한다.

1. 2형 당뇨병 이외의 당뇨병(1형 당뇨병, 당뇨병성 케톤산증, 임신성 당뇨병 등)인 자
2. 엔블로정 또는 엔블로멧서방정의 허가사항에 따라 투여 금기에 해당하는 자

- 엔블로정 또는 엔블로멧서방정 구성성분에 과민반응 및 그 병력이 있는 환자
- 사구체여과율(eGFR, estimated Glomerular Filtration Rate) 30 mL/min/1.73m^2^ 미만인 환자
- 사구체여과율(eGFR, estimated Glomerular Filtration Rate) 60 mL/min/1.73m2 미만의 신장애 환자, 말기 신질환 또는 투석중인 환자
- 중등증 및 중증의 간장애 환자(AST 또는 ALT > 정상 상한치의 3배, Total Bilirubin > 정상 상한치의 2배, 간염 또는 간부전)
- 뉴욕심장학회(New York heart association, NYHA)의 분류에 의한 class III 혹은 IV인 자

1. 등록 시점 3개월 이내부터 비만치료제 또는 체중감량 약물을 사용한 치료 또는 기타 치료(수술, 식이요법 등)으로 인해 체중이 불안정한 환자
2. 심신미약자
3. 임부 및 수유부
4. 다른 임상시험에 참여하여 임상시험용의약품 또는 임상시험용의료기기를 투여(적용)중인 자
5. 연구 등록 전 웰체크 사용 경험이 있는 자
6. 기타 연구자(담당의사)의 판단에 따라 본 임상연구에 참여하는 것이 적합하지 않은 것으로 판단된 자

## 연구 완료 및 중지/탈락

1. **연구대상자의 연구 완료 및 연구 종료**

연구대상자의 최초방문(Baseline) 후 24주 시점의 추적방문 절차가 완료되면 해당 연구 대상자는 임상연구를 완료한 것으로 간주한다. 본 임상연구에서는 마지막 연구대상자의 임상연구 종료를 전체 임상연구의 종료로 정의한다.

1. **연구대상자 연구 중지/탈락**

연구기간 동안 연구대상자는 본인의 의지로 언제든지 임상연구를 중단할 수 있으며, 안전, 행위 또는 행정상의 이유로 연구자 또는 의뢰자의 재량에 의해 언제든지 중지/ 탈락할 수 있다. 임상연구에서 연구대상자가 중지/탈락될 수 있는 경우는 다음과 같으며, 연구자는 연구대상자의 중지/탈락하는 경우, 해당 시점까지 수집된 연구자료를 eCRF에 작성한다.

1. 임상연구 진행 도중 연구대상자의 선정/제외기준 위반을 발견한 경우(Not meet the inclusion/exclusion criteria)
2. 연구대상자 또는 연구대상자 대리인의 동의철회[Withdrawal by Subject (or his/her legal representative)]
3. 연구대상 소프트웨어 비순응(Non-Compliance with Investigational Product)

: 연구대상자가 연구대상 소프트웨어와 관련된 연구자의 지시에 동의하지 않거나 따르지 않은 경우

1. 추적불능(Lost to follow-up)

: 연구대상자의 추적이 불가능한 경우

1. 연구자 판단(Physician Decision)

연구대상자의 중지/탈락이 발생하는 경우, 연구자는 의뢰자에게 통보하고 임상연구 종료평가를 완료하도록 최선을 다해야 한다. 중지/탈락 사유를 근거문서와 eCRF에 기록하여야 한다.

## 임상연구의 중단

연구책임자, 연구자(IRB 승인 및 연구책임자가 위임한 연구담당자)는 임상연구 과정에서 수집된 결과에 비추어 임상연구를 지속하는 것이 바람직하지 않다고 판단될 경우, 연구 지원기관(의뢰자)와 협의하여 임상연구를 조기 중단 또는 일시 중지 할 수 있으며, 의뢰자는 안전성 혹은 관리상의 이유로 임상연구를 조기중단 또는 일시 중지 시킬 수 있다. 연구책임자는 이 사실을 IRB에 즉시 보고하고, 조기중단 또는 일시 중지에 대한 상세한 사유를 제출 해야 한다.

의뢰자에 의한 연구중단 사유는 다음과 같다.

1. 본 연구대상의약품의 품목 승인이 취소되거나 판매를 중지하는 경우
2. 의뢰자의 판단 상 본 연구의 진행이 의학적 또는 윤리적으로 정당화되지 않는다고 판단한 경우.
3. 연구대상자 모집이 지나치게 지체되어 연구의 진행이 어렵다고 판단되는 경우 등

어떠한 이유로든 연구가 조기 중단 또는 일시 중지된 경우 연구자는 연구대상자에게 이 사실을 알리고 적절한 조치와 추적방문이 이루어질 수 있도록 한다. 연구자는 중단된 시점까지 진행된 연구대상자에 대한 eCRF, 연구 진행현황 및 결과를 정리하여 의뢰자에게 전달한다.

# 임상연구 방법

## 전반적인 임상연구 디자인

본 임상연구는 1차 의료기관의 실제 진료환경에서 2형 당뇨병 환자를 대상으로 24주간 ‘웰체크’ 사용 시 연구자와의 연동 관리 여부에 따른 혈당, 혈압, 체중의 개선 효과를 확인하기 위한 일차의료기관 기반, 전향적, 다기관, 군집 무작위배정, 실용 임상연구로 설계하였다.

일상적인 진료 방문 기간 동안 연구자(담당의사)의 의학적 판단에 따라 2형 당뇨병의 치료로 엔블로정 또는 엔블로멧 서방정을 투여중이거나, 투여가 가능하다고 판단되는 환자 중에서 스마트폰을 이용한 디지털 헬스케어 스마트폰 어플리케이션을 사용하는데 어려움이 없는 환자가 본 연구에 등록 가능하다.

단, 기관에서 시험군과 대조군을 무작위배정하여 배정군에 따라 별도 관리하기 어려운 1차 의료기관의 현실적 상황을 반영하여, 군집 무작위배정 방법을 통해 시험군[연구자(담당의사) 연동 관리 ‘웰체크’ 사용군] 기관과 대조군(연구대상자 단순 자가관리 ‘웰체크’ 사용군) 기관을 구분하여 기관을 선정한 후 연구를 수행하고자 한다.


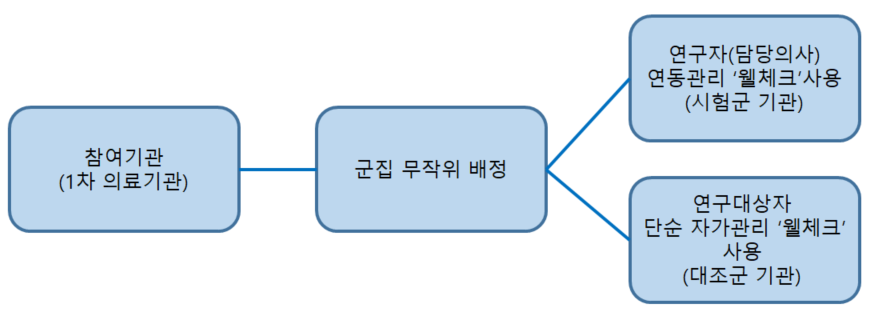
그림 1. 임상연구 설계

본 임상연구에서 수집되는 자료는 실제 진료환경에서 기록되는 의무기록을 근거로 수집하며, 인구학적 정보, 신체계측, 활력징후, 검사실 검사 등의 정보를 수집하며, 본 임상연구에서 의무적으로 규정된 방문 및 검사 또는 처치는 없다.

그러나 진료 환경에 따라 연구대상자 등록일(Baseline)로부터 최대 24주 기간 동안 전향적으로 의무기록 자료를 수집하는 추적방문이 진행 될 예정이다.

대상자 별 전향적 의무기록 자료 수집 시점은 등록일(Baseline, 0일), 방문2(추적방문, 6주), 방문3(추적방문, 12주), 방문 4(추적방문, 18주), 방문5(최종, 24주)시점으로 계획하였으며, 이 중 방문 2, 방문 4는 연구자(담당의사)의 판단에 따라 선택적으로 수집할 수 있으며, 유선을 통한 추적방문을 허용한다.


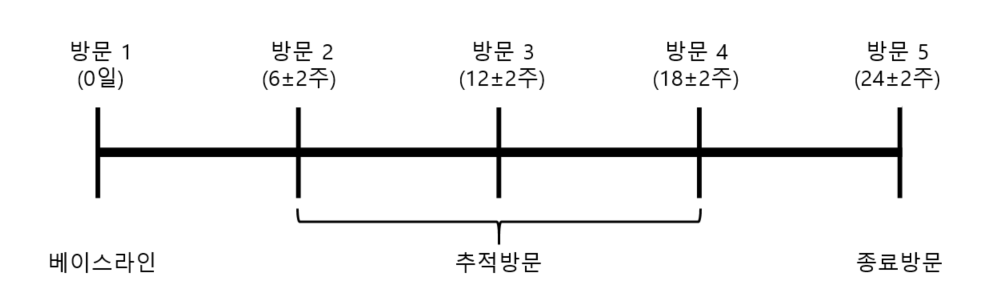


그림 2. 임상연구 흐름도

## 임상연구 기간

본 연구 기간은 IRB승인일로부터 2026년 12월 31일까지 진행될 예정이다. 각 연구대상자 별 자료수집 기간은 최대 24주(6개월)로, 해당 기간 동안 연구계획서에 명시된 정보를 수집한다. 단, 연구대상자 선정의 어려움 등으로 인해 연구 진행에 영향을 미칠 수 있는 상황이 발생한 경우에는 기간의 변동이 있을 수 있다.

## 임상연구의 진행 및 일정

### 방문 일정

본 연구에서 의무사항으로 규정된 방문, 검사, 검사실적 검사 또는 처치는 없다. 통상적인 진료방문 시 진료 기록, 연구자 평가, 검사실 검사치 등을 이용하여 전향적으로 수집할 것이다. 각 연구자들은 베이스라인과 추적방문 기간 동안 연구대상자들에 대해 사전에 규정된 정보를 수집할 것이다.

1. **방문1(베이스라인, 0일)**

최초방문(베이스라인)에 아래 각 항목에 대한 정보를 수집한다.

- 연구대상자 동의 취득 및 연구대상자 등록 번호 부여
- 선정/제외기준 확인
- 디지털 헬스케어 스마트폰 어플리케이션 ‘웰체크’ APP설치 및 병원 연동
- 인구학적 정보 (이니셜, 성별, 연령(생년월), 임신 여부 및 수유 여부)
- 2형 당뇨병 정보 (진단일)
- 병력 정보 (대상 질환을 제외한 등록 전 6개월 이내의 과거병력 및 현재 병력)
- 음주/흡연/생활습관 정보
- 신체계측 (신장, 체중, 체질량지수)
- 활력징후 (수축기/이완기 혈압, 맥박)
- 체성분 분석계 검사 (골격근량, 체지방량, 체지방률, 근육량, 허리/엉덩이 둘레, 체수분, 세포내수분, 세포외수분, 세포외수분비, 복부지방률 등 포함)
- 선행 (등록 전 4주 이내 당뇨병치료제)약물/ 병용약물(등록 시점 복용중인 당뇨병치료제를 포함한 모든 병용 투여 약물)
- 검사실 검사 (HbA1c, FPG, Total Cholesterol, LDL-C, HDL-C, Triglyceride, ALT, AST, Creatinine, e GFR, UACR, UGCR 등)

1. **추적방문**

추적방문은 최초방문(베이스라인)으로부터 6주 간격으로 방문2(6주±2주), 방문3(12주±2주), 방문4(18주±2주), 방문 5(24주±2주)를 실시할 수 있으며, 방문 2와 방문 4는 연구자(담당의사)의 판단에 따라 선택 방문 및 유선을 통한 자료 수집이 가능하다. 추적 방문 시 아래 각 항목에 대한 정보를 수집한다.

- 음주/흡연/생활습관 정보
- 신체계측 (체중, 체질량지수)
- 활력징후 (수축기/이완기 혈압, 맥박)
- 체성분 분석계 검사 (골격근량, 체지방량, 체지방률, 근육량, 허리/엉덩이 둘레, 체수분, 세포내수분, 세포외수분, 세포외수분비, 복부지방률 등 포함)
- 병용약물
- 검사실 검사 (HbA1c, FPG, Total Cholesterol, LDL-C, HDL-C, Triglyceride, ALT, AST, Creatinine, e GFR, UACR, UGCR 등)
- (연구 종료 시) 웰체크 사용 경험 만족도 설문

## 수집정보 및 항목

1. **서면동의 취득 및 연구 대상자 스크리닝번호 부여**

연구대상자는 내원한 외래 환자를 대상으로 하며, 필요 시 기관 상황에 따라 원내 게시판의 모집공고문을 게시하여 대상자의 자발적인 연구 참여를 통해 모집한다.

연구자는 본 연구와 관련된 자료를 수집하기 전 연구대상자에게 대상자 설명문을 통해 본 연구의 목적과 내용에 대하여 상세히 설명하고, 연구대상자의 자발적인 동의를 얻고 연구대상자의 성명과 서명, 서명일이 포함된 동의서를 득한다.

서면동의일과 방문1(베이스라인, 0일)은 상이할 수 있으나, 임상연구 참여 전 반드시 동의 취득이 선행되어야 한다.

대상자의 스크리닝 번호는 연구대상자 동의 취득 후 e CRF에 대상자 등록 시 자동으로 부여되며, 대상자 번호는 ‘AXX-ZZZ’ 형식의 총 6자리로 다음과 같이 구성된다.

- ‘A’: 분류기호 (A, B)
- ‘XX’: 연구기관 번호 (01, 02 ~)
- ‘ZZZ’: 각 기관별 동의한 연구대상자 번호 (001, 002 ~)

1. **선정/제외기준 확인**

선정/제외기준 확인은 방문1(베이스라인, 0일) 시점에 선정기준을 모두 만족하는지 여부, 제외기준 중 어느 하나라도 해당되는지 여부를 확인한다.

1. ‘**웰체크’ APP 설치 및 가입**

연구에 등록된 대상자는 스마트폰 어플리케이션 ‘웰체크’를 설치하고, 회원가입을 진행 후 연구용으로 부여된 기관 코드를 입력하여 병원 연동을 시행한다.

**[시험군]**

시험군의 스마트폰 어플리케이션 ‘웰체크’ 기본 사용법은 다음과 같이 권고하며, ‘웰체크’ 기능 내에서 대상자의 의학적 상태를 고려하여 연구자 및 대상자가 자유롭게 사용한다. 대상자로부터 ‘웰체크’의 [혈당], [혈압], [체중], [복약 기록] 등의 만성질환 관리를 위한 자가 기록이 누락 되거나, 주기적으로 발송되는 [건강 교육] 등의 이수가 적절히 이루어지지 않을 시, 연구자가 대상자에게 ‘웰체크’의 메시지 발송 기능으로 ‘웰체크’를 통한 자가 관리 및 교육 이수 등을 독려할 수 있다.

1. 방문1(베이스라인, 0일)

- 대상자: ‘웰체크 어플리케이션’ [기초 문진], [건강검진 결과], 시행, [복약 알람] 설정
- 연구자(담당의사): ‘웰체크의사웹’ [혈액 검사 결과] 입력, [심혈관질환 합병증 위험도(ASCVD risk)] 평가, 연동된 대상자의 병력, 생활습관, 증상 확인하여 상담 시 활용
- ‘별첨 6. 대상자 웰체크 사용 가이드’ 팜플렛 제공 및 안내

1. 방문 2 (추적방문, 6주) ~ 방문 5(종료방문, 24주)

- 대상자: 내원 시 ‘웰체크 어플리케이션’ [내원 전 문진] 입력
- 연구자(담당의사): ‘웰체크의사웹’ 환자 자가기록 확인 및 분석결과(문진 기록 혈당, 혈압, 체중기록, 복약기록), 최근 증상 및 생활습관(음주/흡연/운동) 피드백, [혈액 검사 결과](검사 시) 입력, [심혈관질환 합병증 위험도(ASCVD risk)] 평가 및 상담 시 활용

1. 연구 기간 내(방문 1 ~ 방문 5)

- 대상자: ‘웰체크 어플리케이션’ [혈당] 입력(일1회 이상), 복약 기록(복약 시), [혈압] 및 [체중] 기록(주1회 이상), [주치의 메시지] 교육 이수(주2회), [건강 교육] 활용
- 연구자(담당의사): ‘웰체크의사웹’ 주의환자^*^ 모니터링(주 1회), 혈당 미측정자 독려 메시지(월 1회)

**저혈당 54mg/dL 이하 또는 고혈당 200mg/dL 이상일 경우 ‘웰체크의사웹’으로 주의환자 알림 발송*

- 대상자 교육자료 발송(주 2회, 자동발송)

**[대조군]**

대조군의 스마트폰 어플리케이션 ‘웰체크’ 기본 사용법은 다음과 같이 권고하며, ‘웰체크’ 기능 내에서 대상자가 자유롭게 자가 기록 형식으로 사용한다. 연구기간 동안 대상자의 ‘웰체크’ 사용에 대한 연구자의 모니터링 및 활용을 시행하지 않는다.

1. 방문 1(베이스라인, 0일) ~ 방문 5(종료방문, 24주)

- ‘웰체크 어플리케이션’ 에 대한 별도의 안내나 설정은 시행하지 않음, 자유롭게 자가 기록 형식으로 사용
- 연구자(담당의사): ‘웰체크의사웹’으로 별도의 관리나 상담 활용을 시행하지 않으며, 일상 진료 시행
- 대상자 교육자료 발송(주 2회, 자동발송)

1. **인구학적 정보**

방문1(베이스라인, 0일) 시점에 대상자의 기초정보를 확인하기 위하여 다음의 항목을 수집한다. 임신 여부는 문진 또는 임신검사 결과가 있는 경우 수집한다.

- 이니셜
- 성별
- 생년월, 연령(만 나이)
- 임신 및 수유 여부

1. **2형 당뇨병 정보**

방문1(베이스라인, 0일) 시점에 2형 당뇨병의 진단일을 수집한다.

1. **병력 조사**

병력의 경우, 방문1(베이스라인, 0일) 전까지 관찰된 대상질환을 제외한 임상적으로 유의한 의학적 상태 또는 비정상으로 정의하며, 방문1(베이스라인, 0일) 전 6개월 이내의 병력 및 현재 진행중인 병력에 대하여 각 병력의 진단명 및 진단일을 수집한다.

1. **음주/흡연/생활습관 정보**

방문1(베이스라인, 0일) 시점에 대상자의 기초정보를 확인하기 위하여 다음의 항목을 수집하고, 이후 모든 방문 시점에 변화된 사항이 있는 경우 추가적으로 수집한다. 통상적인 진료 방문에서의 문진과 진료기록을 통해 가능한 확인하며, 시험군의 경우 ‘웰체크’ 어플리케이션 내 수집된 자료를 활용할 수 있다.

1. 음주력
   - 현재 음주^*^: 평생 12잔(Units) 이상 음주하였고, 12개월 이내에 1회 이상 음주
   - 과거 음주: 평생 12잔(Units) 이상 음주하였으나, 12개월 이내 음주하지 않음
   - 비음주: 평생 12잔(Units) 미만으로 음주

^*^ 현재 음주자의 경우, 주당 음주 횟수, 1회당 음주량을 확인한다.

1. 흡연력
   - 현재 흡연: 평생 담배 5갑(100개피) 이상 피웠고, 30일 이내 흡연
   - 과거 흡연: 평생 담배 5갑(100개피) 이상 피웠고, 30일 이내 흡연하지 않음
   - 비흡연: 평생 담배 5갑(100개피) 미만으로 흡연
2. 생활습관(식습관): 불규칙한 식사, 과식, 탄수화물/당 과다섭취 여부, 지방 과다섭취 여부, 염분 과다섭취 여부
3. 생활습관(운동습관): 운동종류(걷기, 유산소운동, 근력운동), 주당 운동 횟수, 운동 강도(30분 이내, 1시간 이내, 장시간)
4. **신체계측**

모든 방문시점에 체중, 체질량지수를 수집하며, 체질량지수는 신장 및 체중을 통해 CRF에 자동으로 계산된다. 신장은 방문1(베이스라인, 0일) 시점 1회 수집한다.

1. **활력징후**

모든 방문시점에 활력징후로 아래의 항목을 수집한다.

- 혈압(수축기/이완기)
- 맥박

혈압 및 맥박은 측정 전 최소 5분 동안 휴식을 취한 후 조용한 환경에서 등받이가 있는 의자에 앉아 측정하는 것을 권장하며, 가능한 측정 전 30분 이내에는 흡연, 알코올, 카페인 섭취를 하지 않도록 한다.

1. **체성분분석계 검사**

측정이 가능한 기관에 한하여 방문1(베이스라인, 0일), 방문3(추적방문, 12주) 및 방문 5(종료방문, 24주) 시점에 아래의 항목을 포함하여 수집한다.

- 체성분분석계 지표: 골격근량, 체지방량, 체지방률, 근육량, 허리/엉덩이 둘레, 체수분, 세포내수분, 세포외수분, 세포외수분비, 복부지방률 등

1. **선행/병용약물**

선행약물의 경우 방문1(베이스라인, 0일) 기준 4주 이내에 투여한 당뇨병치료제에 한하여 수집하며, 병용약물은 방문1(베이스라인, 0일) 이후 연구 기간 동안 3개월 이상 꾸준히 복용하는 2형 당뇨병 치료 목적 약물을 포함 모든 병용약물에 대한 투여 정보를 수집한다.

- 약물명(상품명)
- 용법·용량 (1회 투여 용량, 단위, 투여 빈도, 투여 경로)
- 투여기간(투여시작일, 투여 종료일, 지속여부)
- 투여 목적
- 용량 변경 또는 투여 중단된 경우 용량 변경 또는 투여 중단 사유

1. **검사실 검사**

검사실 검사는 실제 진료환경에서 통상적인 진료 방법에 따라 검사를 수행한 경우 의무기록을 토대로 수집하며, 방문1(베이스라인, 0일) 기준으로 4주 이내 수집된 HbA1c 결과 및 그 외 3개월 이내 수집된 검사 결과가 있는 경우, 해당 검사 결과로 방문1(베이스라인, 0일) 검사실 검사를 대체할 수 있다.

심혈관질환 합병증 위험도(ASCVD risk)는 ASCVD 2013 Risk Calculator from AHA/ACC 공식을 따르며, eCRF내 수집된 정보(성별, 연령, 수축기 혈압, Total Cholesterol, HDL-C, 고혈압 약물치료 여부, 당뇨병 진단 여부, 흡연 여부 항목)를 토대로 연구 종료 후 분석을 시행할 예정이며, 시험군은 ‘웰체크’ 어플리케이션 내 자동 계산 기능으로 연구자(담당의사)가 콜레스테롤, 혈압 값을 입력하여 대상자 상담 시 사용이 가능하다.

수집될 수 있는 검사실 검사 항목의 예시는 아래와 같다.

| 주요 검사실 검사 | HbA1c, FPG |
| --- | --- |
| 기타 검사실 검사 | Total Cholesterol, HDL-C, LDL-C, Triglyceride, ALT, AST, γ-GTP, Creatinine, e GFR, UACR, UGCR |

1. **웰체크 사용 경험 만족도 설문**

의료진 및 대상자의 웰체크 사용 경험 만족도 설문은 방문5(종료방문, 24주)에 서면으로 시행한다.

의료진 및 대상자의 웰체크 사용 경험 만족도 설문은 각 10개의 문항과 5개의 척도로 구성된 사용 만족도 평가도구로서 어떤 특정 질환군을 목표로 하는 것이 아닌, 웰체크 어플리케이션을 사용함으로 인한 만성 질환자 관리의 효과성 및 업무효율 개선, 사용 경험 만족 여부를 확인할 수 있는 일반적인 설문이다.

의료진의 웰체크 사용 경험 만족도 설문 10개의 문항은 어플리케이션을 사용함으로써 만성질환 환자 관리의 효과성, 의료진의 업무 효율성의 개선이 있었는지 등을 확인하고자 하며, 5개의 척도는 1) 전혀 그렇지 않다 2) 그렇지 않다 3) 보통 4) 그렇다 5) 매우 그렇다 로 구성되어 있다.

대상자의 웰체크 사용 경험 만족도 설문 10개의 문항은 어플리케이션을 사용함으로써 의료진과의 관계 만족도, 치료 과정 경험 등이 도움이 되었는지를 확인하고자 하며, 5개의 척도는 1) 전혀 그렇지 않다 2) 그렇지 않다 3) 보통 4) 그렇다 5) 매우 그렇다 로 구성되어 있다.

## 무작위 배정

본 임상연구는 기관에서 시험군과 대조군을 무작위 배정하여 배정군에 따라 별도 관리하기 어려운 1차 의료기관의 현실적 상황을 반영하여, 군집 무작위배정 (cluster randomization)방법을 통해 시험군[연구자(담당의사) 연동 관리 ‘웰체크’ 사용군] 기관과 대조군(연구대상자 단순 자가관리 ‘웰체크’ 사용군) 기관을 구분하여 기관을 선정한 후 연구를 수행하고자 한다.

본 연구의 군집 무작위배정은 참여하는 1차 의료기관의 기본 특성[진료의사의 연령 (45세 이상, 이하), 지역 (수도권, 비수도권)]에 대한 소집단으로 구분하고, 이러한 소집단별로 시험군과 대조군을 무작위 할당하여 시험군 군집 또는 대조군 군집에 1:1의 비율로 군집 무작위배정을 실시한다.

엄격한 무작위배정을 위해, 본 임상연구와 관련이 없는 무작위배정 담당자가 독립적으로 무작위배정 목록을 작성하며 이를 위하여 SAS(Ver. 9.4 이상, SAS Institute, Cary, NC, USA)의 PLAN 프로시저(Proc Plan procedure)를 사용한다. 생성된 무작위배정 목록은 IWRS(interactive web response system) 개발자에게 제공한다. 무작위배정 번호의 확인은 IWRS를 통하여 진행한다.

대상자가 본 임상연구에 참여할 것을 서면 동의하면 대상자의 스크리닝 검사 결과를 평가한다. 선정기준에 적합하고 제외기준에 해당하지 않는 대상자에 한하여 무작위배정 등록 번호(Randomization Enrollment Number)를 부여한다.

무작위배정 등록 번호는 ‘AAA-RXX-YYY’와 같이 부여되며 다음을 의미한다.

- ‘AAA’: 연구기관 구분 번호- ‘R’: Cluster Randomization을 의미- ‘XX’: 층화 구분자 [진료의사의 연령 (45세 이상, 이하), 지역 (수도권, 비수도권)]

- ‘YYY’: 무작위배정을 받은 시험대상자 순서

|  | 수도권 (X1) | 비수도권 (X2) |
| --- | --- | --- |
| 45세 이상 (1X) | 11 | 12 |
| 45세 이하 (2X) | 21 | 22 |

# 연구대상 소프트웨어

## 연구대상 소프트웨어의 정보

**
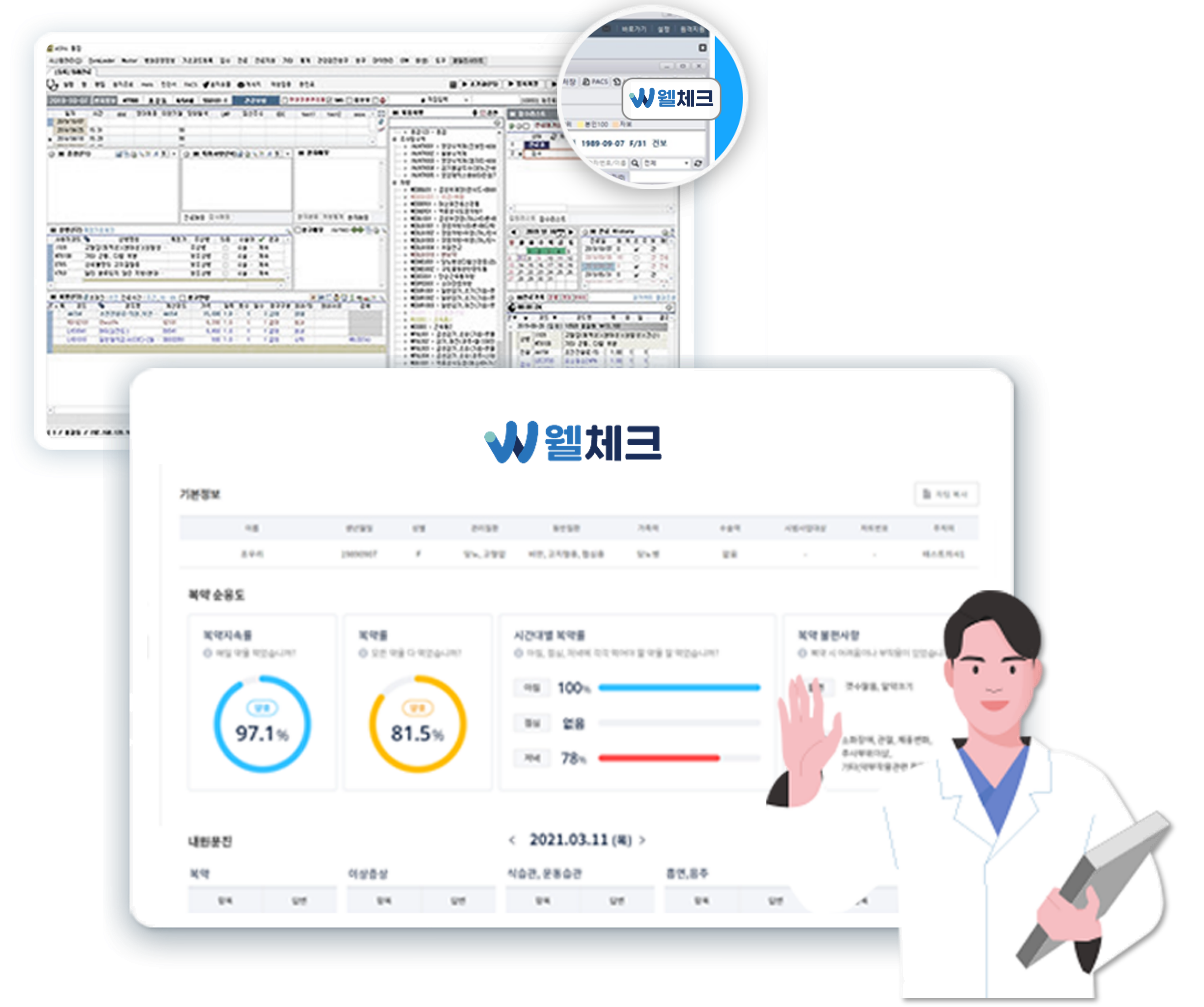
**

병원연계 디지털헬스 어플리케이션 웰체크(Well Check)는 '환자용 어플리케이션(App)'과 '의사용 웹(Web) 및 전자의무기록(EMR)'을 연결하여 당뇨병, 고혈압을 관리할 수 있다는 점에서 다른 단순 디지털헬스 어플리케이션과 차별화된다. 의사는 웰체크를 이용해 환자의 건강상태를 편리하게 확인하고 ‘의사용 Web’에서 관리할 수 있다.

웰체크는 사용자가 문진을 통해 혈압과 혈당 및 특정 건강 문제를 기록할 수 있으며, 혈압과 혈당이 주 1회 이상 기록되지 않으면 사용자에게 알람을 보낸다. 그런 다음 데이터를 의사에게 전송하여 분석 및 모니터링할 수 있다. 사용자가 목표치에 비해 혈압이나 혈당이 너무 높거나 낮게 기록되면 의사는 판단에 따라 피드백 메시지를 보내 관리할 수 있다. 이를 통해 의사는 환자를 관리하고 위험 환자를 별도로 모니터링할 수 있다. 또한 사용자는 목표 대비 자신의 수치가 얼마나 효과적으로 관리되고 있는지 그래프, 목록, 표 중 원하는 방식으로 확인할 수 있다. 또한, 국민건강보험공단에서는 환자가 전 생애에 걸쳐 시행한 건강검진 결과를 연동하여 주치의에게 제시하고 주치의로부터 피드백 메시지를 받을 수 있도록 하고 있다.

1. **웰체크 어플리케이션의 주요 기능**
2. 병원 차트(EMR) 연동 – 이지스, 씨차트, 비트A, 센스, 위하고H
3. 혈압, 혈당, 복약, 동반증상, 생활습관 등 분석
4. 주의환자 모니터링, 필요시 내원 알림 기능
5. 주치의 메시지 발송
6. 건강검진 결과 연동
7. 검사 추천, 교육
8. 연속혈당 리포트 분석
9. 해외 진료지원 서비스
10. 비만 관리
11. **웰체크 어플리케이션의 활용방법**
12. 병원 계정 가입 및 설정

- 최초 가입(<https://www.well-check.co.kr>) 🡪 의사용 Web 접속 🡪 병원 프로필 설정 🡪 그외 병원 설정(치료목표, 모니터링 기준, 검사 설정)

1. 첫 진료 시
2. 환자와 병원 연결 (환자 App – 의사 Web 연동)
3. 환자의 병력 청취, 생활습관 평가
4. 당뇨병 관리 검사 추천/교육
5. 건강검진 결과 연동
6. 기초 문진, 검사실 검사매 진료 시 – 환자 자가기록 확인, 상담
7. 환자 증상, 생활습관 파악
8. 혈당 Overview: 식전/식후, 혈당추세, 예상 당화혈색소의 변화
9. 혈당/혈압/복약기록 함께 확인: 복약 알림 기능 사용 중인 환자
10. 복약 순응도 분석
11. 검사 시 – 원내 검사 결과 전송
12. 환자별 검사 내역 전송, 상담
    - 원내에서 측정한 혈당/당화혈색소, 혈중 지질, 간기능 검사결과, 체성분(인바디) 결과를 의사 Web에 입력하여 환자에게 전송, 과거 기록 누적 관리
13. 심혈관질환 합병증 위험도 계산: 콜레스테롤, 혈압 값 입력
14. 건강검진결과 확인
15. 진료 후 관리
16. 당뇨병 교육 컨텐츠 제공 (주 2회)
    - 질환 정보, 식습관, 운동, 합병증 관리
    - 교육컨텐츠 완독률 확인 가능
17. 주의환자 모니터링 (주 1회)

- 혈당, 혈압, 체중 관리 목표/주의 알람 기준 설정, 목표치를 벗어날 경우 주 1회 모니터링 및 관리
- ‘메시지 보내기’ 기능 및 ‘비대면 상담’ 기능

1. 혈당, 혈압, 체중 자가기록 입력 순응도 모니터링 (주 1회)

- 기록 미입력자 조회 및 입력 독려 메시지 전송 (관리 환자는 주 1회 이상 자가기록 입력하도록 관리, 월 1회 합병증 체크)

## 연구대상 소프트웨어의 사용

본 임상연구에서 연구대상자의 ‘웰체크’ 사용은 실제 진료환경에서 각 대상자의 상황에 따라 연구대상 소프트웨어의 기능 범위 내에서 전적으로 연구자(담당의사)가 대상자의 의학적 상태를 고려하여 적절히 결정한다.

#

# 자료 관리

## 근거 문서

근거문서는 본 연구의 자료수집 활동의 결과물로 정의하고, 다음에 해당하는 것을 근거자료 등 임상연구 관련 자료로 규정한다.

1. 근거문서(Source document) 자료 및 기록: 연구대상자 설명서 및 동의서, 진찰기록 등 의무기록, 검사실 검사 기록, 임상연구에 관여하는 약제부 및 검사부 등에 보관되어 있는 기록 등
2. 임상연구를 재현 또는 평가하는 데 필요한 관련 임상 소견, 관찰, 그 밖의 행위 등이 기록된 원본 또는 원본의 공식 사본에 담겨있는 모든 정보.

본 연구에 존재하는 모든 근거 문서는 연구기관의 연구자에 의해 기록 및 보관되며, 근거문서에는 권한이 있는 자만 접근 및 열람이 가능하다.

## 자료 입력

의뢰자 또는 업무를 위임받은 자는 표준작업지침서(Standard Operating Procedures, SOP)에 따라 자료 관리를 수행한다. 모니터 요원은 eCRF와 근거문서의 일치 및 기재 내용의 적절성을 확인하고 eCRF를 회수 또는 추가 수정할 수 없도록 조치를 취한다. 또한, 자료 관리 담당자는 eCRF에 기재된 내용의 적절성과 그 기재 내용이 데이터베이스에 정확하게 입력되어 있는 지의 여부를 점검하고, 데이터베이스 상에서 논리적 모순의 유무를 확인한다.

본 연구에서 수집하고자 하는 자료는 사전에 고안된 e-CRF를 사용하여 수집할 것이며, 사용하는 모든 전자 데이터 수집(Electronic Data Capture, EDC) 체계는 미국연방정부규정(Code of Federal Regulations, CFR)(21 CFR Part 11) 및 임상연구 전자자료의 처리 및 관리를 위한 가이드라인을 따른다. EDC 시스템은 공인된 전자 자료 수집체계로 권한이 부여된 경우에만 접속할 수 있으며, EDC 시스템을 통해 e-CRF를 입력, 수정, 저장, 삭제하는 모든 추적 사항이 기록될 것이다. 의뢰자는 수집된 자료를 처리하는 과정에서 자료의 확인 또는 수정을 위한 요청을 할 수 있다. 연구자는 해당 확인을 요청 받은 자료에 대해 재확인하거나 수정함으로써 요청에 응답할 책임이 있으며, 전자서명을 통해 입력한 e-CRF의 자료가 정확하고, 완전하며, 해독이 가능하고, 시기 적절하다는 것을 보증한다. EDC 시스템을 통해 작성된 e-CRF는 연구 종료 후, 사본을 전자 저장 매체에 담아 각 연구기관에 전달하며, 다른 기본문서들과 동일한 기준으로 보관될 것이다.

## 자료 확인

eCRF와 근거문서의 불일치, 기재 내용의 비 적절성 및 논리적 모순 등이 확인되는 경우 의뢰자 또는 자료 관리 담당자는 연구책임자와 함께 해당 항목의 타당성을 검토하고 필요한 경우 문서를 통해 기록을 정정하도록 한다.

의뢰자는 eCRF 및 데이터베이스에 오류가 없는 것을 확인한 후에 실수 또는 무단으로 자료가 변경되지 못하도록 데이터베이스를 잠금(Database lock)한다. 잠금 이후에는 임의로 임상연구 자료를 수정할 수 없다.

## 자료 보관

연구책임자는 연구 참여에 대한 동의 철회 및 중도탈락 대상자를 포함하여 각 기관에 수집되는 모든 자료 및 기록(전자문서를 포함)을 보관하고 관리해야 하는 책임이 있다.

연구 기간 동안 수집된 모든 문서는 연구책임자 및 공동연구자, 위임을 받은 연구담당자만 접근이 가능한 시건장치가 있는 장에 보관하도록 한다.

생명윤리 및 안전에 관한 법률 시행규칙 제15조에 따라 연구책임자는 임상연구 종료일 기준으로 3년 동안 보관해야 한다(단, 의뢰자가 필요하다고 판단하는 경우 보관기간을 연장할 수 있다).

이러한 문서는 의뢰자나 관련 규제 당국의 실태조사 시 조사의 대상이 되며, 연구자는 의뢰자의 서면 허가 없이 임상연구와 관련된 어떤 문서도 파기해서는 안 된다. 연구자는 이런 문서들이 사고나 조기 파손되지 않도록 예방책을 강구해야 한다.

자료의 보관기간이 만료되면 의뢰자와의 협의에 따라 종이문서는 즉시 파쇄하고 전자문서는 복원 또는 재생되지 않도록 파기될 것이다.

## 자료 열람

본 연구에 관련된 의뢰자, 모니터요원 및 점검자는 본 연구의 모니터링과 점검 및 진행사항 관리를 위한 목적으로 연구대상자의 기록을 열람할 수 있다. 연구자는 본 연구의 계약이 체결됨으로써 의뢰자 그리고/또는 임상시험 수탁기관의 모니터요원 및 점검자가 연구대상자의 차트와 증례기록서 기록을 검증하기 위하여 해당 문서를 열람하여 검토할 수도 있음을 숙지하여야 한다.

이러한 정보들은 기밀로 보관되어야 하며, 기밀 보관을 위한 시설과 관리 기준을 갖추고 있어야 한다. 연구자는 의뢰자 그리고/또는 임상시험수탁기관에게 필요한 지원을 보장하여야 한다. 연구자는 의뢰자, 규제기관, IRB의 권한이 있는 대리인이 연구 관련 절차와 데이터 확인을 위해 연구대상자의 원본 의무 기록에 직접 접근하여 검토할 수 있도록 승인해야 한다.

# 평가기준 및 평가방법과 통계분석 방법

## 평가변수

1. **유효성 평가 변수**
2. **1차 유효성 평가변수**

- 베이스라인 대비 24주 시점의HbA1c 변화율 및 변화량

1. **2차 유효성 평가변수**
   1. 베이스라인 대비 6, 12, 18, 24주 시점의FPG 변화율 및 변화량
   2. 베이스라인 대비 6, 12, 18주 시점의HbA1c 변화율 및 변화량
   3. 베이스라인 대비 6, 12, 18, 24주 시점의HbA1c <7% 달성 대상자 비율
   4. 베이스라인 대비 6, 12, 18, 24주 시점의HbA1c <6.5% 달성 대상자 비율
   5. 베이스라인 대비 12, 24주 시점의 치료적 반응[베이스라인 대비 각 평가 시점에서 HbA1c 변화량(베이스라인의 HbA1c–각 평가 시점의 HbA1c)> 0.5 % 또는 HbA1c < 7 %] 달성 대상자 비율
   6. 베이스라인 대비 6, 12, 18, 24주 시점의 혈압(수축기혈압, 이완기혈압) 변화량
   7. 베이스라인 대비 6, 12, 18, 24주 시점의 체질량지수(BMI) 및 체중 변화량
   8. 베이스라인 대비 6, 12, 18, 24주 시점의 5%이상 체중 및 BMI 감소 대상자 비율
2. **탐색적 평가변수**
3. 베이스라인 대비 6, 12, 18, 24주 시점의 지질농도(Total cholesterol, LDL-C, HDL-C, Triglyceride)의 변화량
4. 베이스라인 대비 6, 12, 18, 24주 시점의 간기능 관련 지표(AST, ALT, γ-GTP)의 변화량
5. 베이스라인 대비 6, 12, 18, 24주 시점의 신기능 관련 지표(e-GFR, UACR(Urine Albumin to Creatinine Ratio), UGCR(Urine Glucose to Creatinine Ratio)) 변화량
6. 베이스라인 대비 12, 24주 시점의 체성분분석계 지표(골격근량, 체지방량, 체지방률, 근육량, 허리/엉덩이 둘레, 체수분, 세포내수분, 세포외수분, 세포외수분비, 복부지방률 등) 변화량
7. 베이스라인 대비 12주, 24주 시점의 심혈관질환 합병증 위험도(ASCVD risk) 변화량
8. **안전성 평가변수**
9. 검사실 검사, 활력징후, 신체검사 결과

## 통계분석 방법

1. **분석군의 정의**

### 유효성 평가 대상자군

선정/제외기준을 만족하여 본 연구에 등록된 안전성 평가 대상자 중에서 베이스라인 이후 HbA1c 검사 수치가 1회 이상 수집된 연구대상자를 대상으로 한다.

### 안전성 평가 대상자군

선정/제외기준을 만족하여 본 연구에 등록된 대상자 중에서 허가사항에 따라 연구대상의약품을 최소 1회 이상 투여하고, 안전성 평가가 이루어진 모든 연구대상자를 대상으로 한다.

1. **분석 방법**
2. **일반적 원칙**

연속형 변수는 기술통계량(대상자의 수, 평균, 표준편차, 중앙값, 최소값, 최대값)을 제시하고, 범주형 변수는 빈도와 백분율을 제시한다. 특별한 명시가 없는 경우 모든 검정은 유의수준 5% 하에서 양측검정을 원칙으로 하며, 모든 p-value에 대해서는 소수점 최대 3자리까지 제시하고, 이외의 경우 소수점 두번째 자리까지 나누어 떨어지지 않을 경우, 소수점 세번째 자리에서 반올림하여 소수점 2자리까지 제시한다.

결측치가 최소이고 균등하게 분포하는 경우, 완전 사례 분석(complete-case analysis) 기법을 사용하며, 결측치가 상당하거나 집단 간에 불균형적으로 분포하는 경우 다중 대체(multiple imputation) 기법을 사용한다. 또한, 다양한 결측치 시나리오에서 결론의 견고성과 타당성을 보장하기 위해 민감도 분석을 수행한다.

1. **인구학적 자료와 베이스라인 특성**

연구대상자의 인구통계학적(연령, 성별 등) 자료와 베이스라인 정보에 대하여 연속형 자료는 평균, 표준편차, 중앙값, 최소값 및 최대값을 제시하고, 범주형 자료의 경우 빈도와 백분율을 제시한다.

시험군과 대조군 간의 베이스라인 비교성을 평가하기 위해 주요 베이스라인 특성(연령, 성별, HbA1c, BMI, 당뇨병 유병 기간 등)에 대한 표준화된 평균 차이(standardized mean difference, SMD)를 계산한다. 군 간에 유의미한 불균형(SMD >0.1)을 보이는 변수는 후속 분석에서 공변량으로 포함한다.

1. **유효성 평가변수**

### 1차 유효성 평가 변수

당화혈색소의 변화 및 변화율에 대하여 베이스라인, 24주 시점에 기술통계량을 제시하고, 베이스라인 대비 24주 시점의 차이에 대하여 변화량은 군집 효과를 명확하게 설명하기 위해 혼합 효과 모형 또는 일반화 추정 방정식(GEE)을 시행하여 분석한다. 베이스라인 시점에 유의미하게 불균형한 것으로 확인된 환자 수준 공변량(SMD >0.1)은 고정 효과(fixed effect)로, 의료기관 간 변동성은 임의 효과(random effect)로 포함한다.

### 2차 유효성 평가 변수

2차 유효성 평가변수 각 항목에 대하여 베이스라인, 12주, 24주 시점에 기술통계량을 제시하고, 베이스라인 대비 24주 시점 혹은 12주 시점의 차이에 대하여 변화량은 군집 효과를 명확하게 설명하기 위해 혼합 효과 모형 또는 GEE을 시행하여 분석한다. 환자 수준 공변량은 고정 효과(fixed effect)로, 기관 간 변동성은 임의 효과(random effect)로 포함한다.

### 탐색적 평가 변수

탐색적 평가변수의 각 항목에 대하여 베이스라인, 12주, 24주 시점에 기술통계량을 제시하고, 베이스라인 대비 24주 시점 혹은 12주 시점의 차이에 대하여 변화량은 군집 효과를 명확하게 설명하기 위해 혼합 효과 모형 또는 GEE을 시행하여 분석한다. 환자 수준 공변량은 고정 효과(fixed effect)로, 기관 간 변동성은 임의 효과(random effect)로 포함한다. 탐색적 평가변수 내의 다중 비교는 Benjamini-Hochberg false discovery rate (FDR) 방법을 사용하여 조정되며, 통계적 유의성은 q ≤ 0.05에서 정의된다.

1. **안전성 평가변수**

### 활력징후

활력징후에 대하여 베이스라인, 6주, 12주, 18주, 24주 시점에 기술통계량을 제시하고 베이스라인 대비 6주, 12주, 18주, 24주 시점의 차이에 대하여 변화량은 군집 효과를 명확하게 설명하기 위해 혼합 효과 모형 또는 GEE을 시행하여 분석한다. 환자 수준 공변량은 고정 효과(fixed effect)로, 기관 간 변동성은 임의 효과(random effect)로 포함한다.

### 검사실 검사

검사실 검사에 대하여 베이스라인, 6주, 12주, 18주, 24주 시점에 기술 통계량을 제시하고 베이스라인 대비 6주, 12주, 18주, 24주 시점의 차이에 대하여 변화량은 군집 효과를 명확하게 설명하기 위해 혼합 효과 모형 또는 GEE을 시행하여 분석한다. 환자 수준 공변량은 고정 효과(fixed effect)로, 기관 간 변동성은 임의 효과(random effect)로 포함한다.

또한, 정상(Normal or NCS), 임상적으로 의미 있는 비정상(CS) 변화를 빈도 및 백분율로 요약하여 제시한다. 또한, 임상적으로 의미 있는 비정상(CS) 항목에 대한 대상자의 상세정보를 목록으로 제시한다.

1. **하위집단 분석**

대상자의 특성에 따른 하위집단 분석이 필요한 경우에는 각 항목에 대해 유효성 평가변수 및 안전성 평가변수의 항목과 동일하게 분석을 진행할 수 있다(예. 성별에 따른 1차 유효성 평가 결과 분석 등).

# 윤리적 고려사항 및 행정적 절차

## 공용기관생명윤리위원회/ 임상시험심사위원회(IRB)

본 임상연구는 실시 전 연구계획서를 포함하여 본 임상연구와 관련된 모든 사항에 대하여 IRB로부터 사전 승인을 받아야 한다. IRB는 연구의 윤리적, 의학적 타당성을 심사하여 연구의 시작 전에 결정된 내용을 연구자 및 연구 지원기관(의뢰자)에게 문서로 전달할 것이다. 또한 임상연구기간 동안, 임상연구계획서의 개정이나 변경이 있는 경우에는 IRB에 제출되어야 하며 승인을 받아야 한다.

## 윤리적 고려사항

본 임상연구는 “헬싱키선언(인간 대상 의학연구 윤리 원칙)”에 기초한 윤리적 원칙 및 생명윤리 및 안전에 관한 법률 및 관계 법령을 준수하여 실시하며, 연구대상자의 권리, 안전, 복지를 우선적으로 고려한다.

## 품질 보증 및 점검

의뢰자는 의뢰자 표준작업지침서에 근거하여 임상연구 품질 보증 및 임상연구 자료의 품질 관리를 이행함에 따라 임상연구의 실시, 자료의 생성, 기록 및 보고 절차가 연구계획서, 생명윤리 및 안전에 관한 법률 및 관련 규정을 준수하여 실시되고 있다는 사실을 보증한다.

점검자(Auditor)는 아래의 점검 절차에 따라 점검을 실시한다.

1. 점검자는 의약품 등의 안전에 관한 규칙 [별표 4] 의약품 임상시험 관리기준에 따라 점검을 실시하고 임상연구의 품질을 보증한다.
2. 점검자는 eCRF가 적절한 절차에 따라 기재, 수정 및 확인, 처리되고 있으며 임상연구 계획 및 목적에 따라 임상연구가 수행되고 있는지를 확인한다.
3. 점검자는 임상연구가 생명윤리 및 안전에 관한 법률 및 관련 규정, 표준작업지침서에 따라 이루어지고 있는지 확인한다.
4. 점검자는 점검보고서를 작성 및 검토한 후 의뢰자에게 송부하며, 정해진 절차에 따라 점검보고서를 확정한 후 점검확인서를 발행한다.
5. 점검을 통해 확인된 위반 사항에 대해 의뢰자는 이를 시정하고 재발 방지 조치를 수행하며, 연구자의 지속적인 위반 또는 중대한 위반이 확인된 경우, 의뢰자는 해당 임상연구 실시기관의 임상연구 참여를 중지시킬 수 있다.

## 연구대상자 동의

연구대상자 설명서 및 동의서는 IRB의 승인 후 사용할 수 있다. 연구자는 헬싱키선언에 근거한 윤리적 원칙 및 생명윤리 및 안전에 관한 법률 기준에 따라 연구대상자에게 정보활용 동의를 받아야 한다. 연구자는 반드시 모든 연구 관련 절차를 시행하기에 앞서 연구대상자(또는 대리인)에게 연구에 대하여 충분히 설명하고 연구대상자로부터 서면동의를 받아야 한다. 동의 취득은 독립된 장소(진료실, 상당실 등)에서 이루어 져야 한다. 연구자는 서명된 동의서 원본을 연구자 파일에 보관하여야 하며, 서명된 동의서 사본 및 설명한 설명문은 연구대상자(또는 대리인)에게 제공하여야 한다.

연구대상자가 이해능력, 의사표현능력 결여 등의 사유로 의사소통이 어려운 경우에는 대상자의 법정대리인의 서면 동의를 얻어야 한다. 법정대리인 또는 법정대리인이 없는 경우 배우자, 직계존속, 직계비속 순으로 하되, 직계존속 또는 직계비속이 여러 사람일 경우 협의하여 정하고, 협의가 되지 아니하면 연장자가 대리인이 된다.

법정대리인이 동의하는 경우에도 가능한 연구대상자도 동의서에 자필로 날짜와 서명을 기재하도록 한다.

연구대상자 또는 대리인이 글을 읽을 수 없는 경우, 참관인(공정한 입회인)을 두어 동의를 얻는 전 과정에 참석하도록 한다. 연구대상자 또는 대리인이 구두로 연구 참여에 동의하고, 가능한 경우 동의서에 자필로 서명한 후 참관인이 서명함으로써 동의서 상의 정보가 정확하게 대상자 또는 대리인에게 설명되었고, 해당 사실을 이해하고 동의 과정이 자유 의사에 의해 진행되었다는 것을 증명한다.

본 연구 진행 중 연구대상자 설명서 및 동의서가 변경될 경우, IRB의 재승인을 받아야 한다.

## 연구계획서 승인 및 변경

연구 시작 전, 연구자는 연구계획서를 비롯한 관련 문서 및 절차, 연구대상자 설명서 및 동의서 등에 대하여 IRB의 승인을 받아야 한다.

승인받은 연구계획서를 변경하여 실시하고자 하는 경우, 임상연구 단계별로 계획서 또는 변경계획서에 대하여 IRB의 승인을 받는다. 연구계획서의 승인 이전에는 연구대상자를 임상연구에 참여시킬 수 없다.

## 임상연구 실시기관 모니터링

의뢰자는 연구대상자의 권리와 복지 보호, 연구책임자가 보고한 임상연구 관련 자료와 근거문서의 대조를 통한 자료의 정확성, 완전성 및 검증 가능성을 확인하고, 승인된 연구계획서 및 관련규정에 따라 수행되는지 확인하기 위하여 모니터링을 실시한다.

임상연구에 대한 모니터링은 의뢰자가 지정한 모니터 요원의 정기적인 임상연구 실시기관 방문과 전화 연락을 통해서 이루어질 것이다. 모니터 요원은 임상연구 실시기관 방문 시 기본적으로 근거문서, 연구대상 소프트웨어 관리 기록, 임상연구 기본 문서 파일 보관 현황 등을 확인한다. 또한, 연구 진행 절차 및 기록을 확인하고 위반 사항 등 문제가 있을 경우, 연구책임자 및 연구담당자와 상의하여 적절하게 수정하고 조치를 취하도록 논의한다.

모니터링 방문은 연구책임자 및 연구담당자와 모니터 요원이 협의하여 적절한 시점에 수행하며, 연구책임자 및 연구담당자는 모니터 요원이 eCRF와 대조·확인할 수 있도록 모든 연구대상자의 근거문서를 열람에 적극 협조한다.

## 기밀 유지 및 연구대상자의 비밀 보장

연구의 자료는 잠금 장치가 있는 연구실에 보관하도록 한다. 연구대상자의 의무기록 번호 및 기관별 등록번호는 연구책임자의 책임 하에 별도의 파일로 보관될 것이며, 이를 암호화하여 임상연구 자료로부터 개인 신상 확인이 불가능하도록 관리할 것이다. 생명윤리법 시행규칙 제15조에 따라 임상연구 관련 기록을 연구가 종료된 시점부터 3년간 보관할 예정이며, 보관 기간이 지난 문서 중 개인정보에 관한 사항은 개인정보보호법 시행령 제16조에 따라 파기할 예정이다. 개인신상 정보가 보관되어 있는 파일은 암호를 설정하여 보관하며, 임상연구의 결과물 발표 시에도 환자의 신상을 알 수 있는 어떠한 정보도 포함되지 않도록 한다.

## 연구대상자 안전보호에 관한 대책

임상연구 실시기관은 본 임상연구의 실시에 필요한 설비와 전문인력을 갖추고, 임상연구를 적절하게 실시할 수 있도록 준비에 완벽을 기해야 한다.

연구자는 연구대상자를 임상연구에 등록하기 전에 각 연구대상자들의 건강상태를 확인하여 임상연구에 참여하기에 적합한지 철저히 확인하여야 한다. 또한, 연구자는 연구계획서를 충분히 숙지하고 연구계획서에 따라 연구를 실시한다.

본 연구는 연구대상자에 대한 진료 및 질환 치료는 본 연구와 독립적으로 진행되어야 하며, 연구기간뿐만 아니라 연구 종료 후에도 임상적 판단 하에 필요한 진료 및 치료를 실시하도록 한다.

본 연구는 임상 환경에서의 진료방식 하에서 일상적인 치료 외에 다른 위험은 존재하지 않는다. 따라서 연구와 관련된 위험이 존재하지 않으므로 연구대상자 보상을 추가적으로 진행할 필요가 없다. 의료법 및 연구책임자의 전문직업 배상책임보험 및 관련기관에서 연구대상자와 참여연구자 모두에 대해 충분히 보호한다.

본 연구는 연구대상자에게 의약품이 제공되지 않으므로 의약품에 대한 보상은 기존 의약품의 법적 책임이 적용된다

## 임상연구 결과의 이용 및 발표

본 연구 수행 기간 동안 발생한 모든 자료와 결과는 의뢰자가 소유하며 이를 보고 및 발표하는 것을 원칙으로 한다. 의뢰자는 연구계획서에 따라 실시된 연구 결과에 대해 결과보고서를 작성하여 연구자에게 알리도록 한다.

연구자는 의뢰자의 사전 서면 동의 없이 본 연구의 결과와 관련한 어떤 출판, 발표 또는 정보 공개를 해서는 안되며, 또한 연구담당자도 이를 준수할 수 있도록 확인해야 한다.

연구자가 본 연구의 결과에 대해 학술 논문 발표 또는 출판 하고자 하는 경우 의뢰자의 승인이 필요하며, 의뢰자는 발표의 허용여부 결정 및 발표하기 전에 발표내용을 검토할 권리가 있다.

# 연구 책임자

## 연구 총괄 책임자 정보

성명: 이상열

소속: 경희의료원 내분비대사내과, 경희디지털헬스센터

직위: 교수, 센터장

주소: (02447) 서울특별시 동대문구 경희대로 23

## 연구 총괄 책임자의 역할 및 책임

연구 총괄 책임자는 본 연구 전체의 질 관리 및 연구대상자의 안전을 보호해야 하는 책임을 갖는다. 연구 주요 문서를 검토하고, 본 연구 수행 중 각 기관에서 발생하는 사항에 대해 총괄하며 연구 참여 기관 및 연구자 사이의 의견 조정의 역할을 수행한다. 또한 연구 수행 전 IRB의 심의 승인을 득하고, 연구 기간 중 발생하는 변경 사항 및 기타 사항을 IRB에 보고할 책임이 있으며, 데이터 분석 및 결과보고서를 작성하여 연구 종료 시까지 연구가 원활히 진행되도록 관리한다.

# 별첨

별첨 1. 연구실시기관 및 연구책임자의 성명

별첨 2. 연구대상자 모집 공고문

별첨 3. 연구대상자 설명서 및 동의서

별첨 4. 연구대상자 만족도 설문지

별첨 5. 의료진 만족도 설문지

별첨 6. 대상자 웰체크 사용 가이드

# 참고문헌

1. International Diabetes Federation. IDF Diabetes Atlas, ninth edition, 2019.

2. International Diabetes Federation. IDF Diabetes Atlas, tenth edition, 2021..

3. 대한당뇨병학회. 당뇨병 진료지침 2019.

4. American Diabetes Association. 9. Cardiovascular disease and risk management: standards of medical care in diabetes—2018. Diabetes care 2018;41:S86-S104.

5. Williams B, Mancia G, Spiering W, Agabiti Rosei E, Azizi M, Burnier M, et al. 2018 ESC/ESH Guidelines for the management of arterial hypertension: The Task Force for the management of arterial hypertension of the European Society of Cardiology (ESC) and the European Society of Hypertension (ESH). European Heart Journal 2018;39:3021-104.

6. Timpel P, Oswald S, Schwarz PEH, Harst L. Mapping the Evidence on the Effectiveness of Telemedicine Interventions in Diabetes, Dyslipidemia, and Hypertension: An Umbrella Review of Systematic Reviews and Meta-Analyses. J Med Internet Res 2020;22:e16791.

7. Cameron JD, Ramaprasad A, Syn T. An ontology of and roadmap for mHealth research. International journal of medical informatics 2017;100:16-25.

8. Ma Y, Cheng HY, Cheng L, Sit JW. The effectiveness of electronic health interventions on blood pressure control, self-care behavioural outcomes and psychosocial well-being in patients with hypertension: A systematic review and meta-analysis. International journal of nursing studies 2019;92:27-46.

9. Jamshidnezhad A, Kabootarizadeh L, Hoseini SM. The effects of smartphone applications on patients self-care with hypertension: a systematic review study. Acta Informatica Medica 2019;27:263.

10. Stevens S, Gallagher S, Andrews T, Ashall-Payne L, Humphreys L, Leigh S. The effectiveness of digital health technologies for patients with diabetes mellitus: A systematic review. Front Clin Diabetes Healthc 2022;3:936752.
