# Supplementary material for: Impact of the WellCheck smartphone app linked to electronic health records on clinical outcomes in patients with type 2 diabetes: Study protocol for primary care-based, prospective, multicenter, cluster-randomized, pragmatic clinical trials
Source: PLoS One. 2025 Aug 7;20(8):e0329003. doi: 10.1371/journal.pone.0329003 (PMC12331031; doi:10.1371/journal.pone.0329003)
Supplement: S2 Protocol — (DOCX) [file pone.0329003.s005.docx]

**Clinical Study Protocol**

**A Primary Care-Based, Prospective, Multicenter, Cluster-Randomized, Pragmatic Clinical Trials to Determine the Effect of Linking data with Investigators When Using the Electronic Health Record-Linked Smartphone Application, ‘Well Check’, on Clinical Outcomes in Patients with Type 2 Diabetes Mellitus**

| **Investigational Software:** | **Digital Healthcare Smartphone Application ‘Well Check’** |
| --- | --- |
| **Protocol Number:** | **DW_ODNENV_DB_01** |
| **Protocol Version:** | **V1.1** |
| **Developer:** | **ODN Co., Ltd.** |
| **Sponsor:** | **Daewoong Pharmaceutical Co., Ltd.** |
| **Date of Protocol Preparation:** | **2024-05-16** |

**Protocol Establishment and Revision History**

| **No.** | **Version No.** | **Version Date** | **Change(s)** |
| --- | --- | --- | --- |
| 1 | 1.0 | 2024-05-02 | Not Applicable (Newly Established) |
| 2 | 1.1 | 2024-05-16 | Change in Detailed Randomization Procedure |

**Sponsor**

| Company Name | Daewoong Pharmaceutical Co., Ltd. | | |
| --- | --- | --- | --- |
| Sponsor Representatives | Chang Jae Lee, Sung Soo Park | | |
| Address | 12, Bongeunsa-ro 114-gil, Gangnam-gu, Seoul, Republic of Korea | Postal Code | 06170 |
| Contact | 02-550-8800 | | |

**Protocol Synopsis**

| **Protocol Number** | DW_ODNENV_DB_01 |
| --- | --- |
| **Clinical Study Title** | A Primary Care-Based, Prospective, Multicenter, Cluster-Randomized, Pragmatic Clinical Trials to Determine the Effect of Linking data with Investigators When Using the Electronic Health Record-Linked Smartphone Application, ‘Well Check’, on Clinical Outcomes in Patients with Type 2 Diabetes Mellitus |
| **Protocol Version** | V1.1 |
| **Date** | 2024-05-16 |
| **Phase and Design** | Primary care-based, prospective, multicenter, cluster-randomized, pragmatic clinical study |
| **Clinical Study Sites** | 24 Primary Care Institutions Nationwide |
| **Objective of Clinical Study** | To confirm the improvement effects on clinical outcomes over 24 weeks in patients with Type 2 Diabetes using “Well Check” with or without investigator-linked management in real-world clinical settings |
| **Sponsor** | Daewoong Pharmaceutical Co., Ltd. |
| **Target Indication** | Type 2 Diabetes |
| **Inclusion/Exclusion Criteria** | **Inclusion Criteria**  Select individuals who meet all of the following criteria:   - Adults aged 19 to 80 years - Patients with Type 2 Diabetes who are currently receiving or are scheduled to receive Envlo Tab. or Envlomet SR Tab. based on the approved conditions - Individuals who have no difficulty using a digital healthcare smartphone application on their smartphone - Individuals who plan to engage in appropriate exercise and dietary therapy for blood glucose control during the clinical study period - Women of childbearing potential and men who agree to use appropriate contraceptive methods or have no plans for pregnancy during the study period   *Hormonal contraceptives, intrauterine device (IUD) or intrauterine system (IUS) implants, vasectomy, tubal ligation, double barrier methods (such as simultaneous use of cervical cap or diaphragm with male condom), etc.   - Individuals who have listened to and understood the detailed explanation of the clinical study, and have voluntarily signed a written informed consent agreeing to participate in the study and to comply with subject precautions during the study period   **Exclusion Criteria**  Individuals who meet any of the following exclusion criteria will be excluded from this clinical study:     - Individuals with diabetes other than Type 2 Diabetes (e.g., Type 1 Diabetes, Diabetic Ketoacidosis, Gestational Diabetes, etc.) - Individuals who fall under the contraindications for administration according to the approved conditions of Envlo Tab. or Envlomet SR Tab. - Patients who have hypersensitivity or a history of hypersensitivity to any component of Envlo Tab. or Envlomet SR Tab. - Patients with an estimated Glomerular Filtration Rate (eGFR) less than 30 mL/min/1.73 m² - Patients with renal impairment with an eGFR less than 60 mL/min/1.73 m², end-stage renal disease, or those on dialysis - Patients with moderate to severe hepatic impairment (AST or ALT > 3 times the upper limit of normal, Total Bilirubin > 2 times the upper limit of normal, hepatitis, or liver failure) - Individuals classified as class III or IV according to the New York Heart Association (NYHA) classification - Patients who have undergone treatment with obesity medications or weight loss drugs, or other treatments (surgery, diet, etc.) leading to unstable weight within 3 months prior to the enrollment date - Individuals with mental incapacity - Pregnant or breast-feeding women - Individuals participating in another clinical trial and receiving (applying) investigational drugs or investigational medical devices - Individuals deemed unsuitable for participation in this clinical study based on the judgment of the investigator (responsible physician) |
| **Target Number of Subjects** | Approximately 480 subjects (considering about 20% dropouts to ensure statistical significance) |
| **Duration of Study** | - Expected Total Duration of Study: Date of first IRB approval to December 31, 2026   (may be subject to change depending on the subject enrollment rate)   - Study Period per Subject: Approximately 24 weeks (6 months) |
| **Investigational Software** | Digital healthcare smartphone application ‘Well Check’ |
| **Clinical Study Design and Methods** | This clinical study is designed as a primary care-based, prospective, multicenter, cluster-randomized, pragmatic clinical trial to confirm the improvement effects on blood glucose, blood pressure, and weight over 24 weeks in patients with Type 2 Diabetes when using ‘Well Check’ with or without investigator-linked management in real-world primary care settings.  Patients with Type 2 Diabetes, who, based on the medical judgment of the investigator (responsible physician), have no difficulty using the digital healthcare mobile application on their smartphones, will be recruited in real-world practice settings.  To confirm the clinical basis of ‘Well Check,’ a prospective, multicenter clinical study will be conducted. However, considering the practical constraints of primary care settings where it is difficult to clearly distinguish test groups from control groups within the same institution, a 1:1 cluster randomization will be employed. This will divide study institutions into those administering to the test group (group using ‘Well Check’ with investigator (responsible physician)-linked management) and those administering to the control group (group using ‘Well Check’ for simple self-management).  This clinical study will collect demographic information, physical measurement, vital signs, and laboratory test results up to 24 weeks after starting the use of ‘Well Check’ in real-world clinical settings. Data will be collected based on medical records documented in real-world clinical settings and data gathered through ‘Well Check.’ There are no mandatory visits, tests, or treatments specifically required by this clinical study.  However, follow-up visits will be conducted to prospectively collect efficacy and safety evaluation data at approximately 6 weeks (±2 weeks), 12 weeks (±2 weeks), 18 weeks (±2 weeks), and 24 weeks (±2 weeks) from the date of subject enrollment (baseline, Day 0). The collected items include basic clinical information such as age, gender, past and current medical history, lifestyle habits (smoking, alcohol consumption, physical activity), previous and concomitant medications, and clinical indicators such as weight, body mass index (BMI), body fat mass (optional via body composition analyzer), fasting blood glucose, glycated hemoglobin (HbA1c), blood pressure (systolic/diastolic), total cholesterol, LDL cholesterol, HDL cholesterol, triglycerides, liver function tests (AST, ALT, γ-GTP), and safety evaluation parameters such as vital signs and laboratory tests. During the study, the investigator will collect the necessary data based on the information gathered during routine clinical care.  Participating Institutions  (Primary Care Institutions)  Cluster Randomization  Investigator  (Responsible Physician)  Use of 'Well Check'  for Integrated Management  (Study Group Institutions)  Study Subjects  Use of 'Well Check'  for Simple Self-Management  (Control Group Institutions)  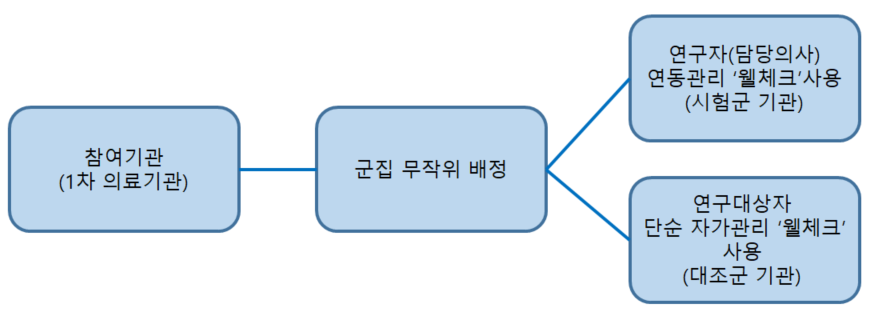<Clinical Study Design>  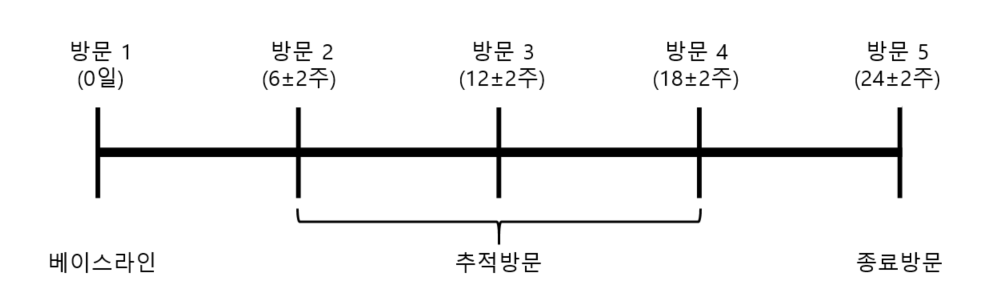  Visit 1  (0 day)  Visit 2  (6±2 weeks)  Visit 3  (12±2 weeks)  Visit 4  (18±2 weeks)  Visit 5  (24±2 weeks)  Baseline  Follow-Up Visits  Close-Out Visit (COV)  <Clinical Study Flowchart> |
| **Efficacy Endpoints** | **Primary Efficacy Endpoint**   1. Percent change and change of HbA1c at 24 weeks compared to baseline   **Secondary Efficacy Endpoints**   1. Percent change and change in FPG at 6, 12, 18, and 24 weeks compared to baseline 2. Percent change and change in HbA1c at 6, 12, and 18 weeks compared to baseline 3. Proportion of subjects achieving HbA1c <7% at 6, 12, 18, and 24 weeks compared to baseline 4. Proportion of subjects achieving HbA1c <6.5% at 6, 12, 18, and 24 weeks compared to baseline 5. Proportion of subjects achieving therapeutic response [change in HbA1c (baseline HbA1c – HbA1c at each evaluation time point) > 0.5% or HbA1c < 7%] at 12 and 24 weeks compared to baseline 6. Change in blood pressure (systolic and diastolic) at 6, 12, 18, and 24 weeks compared to baseline 7. Change in BMI and weight at 6, 12, 18, and 24 weeks compared to baseline 8. Proportion of subjects with a reduction of 5% or more in weight and BMI at 6, 12, 18, and 24 weeks compared to baseline   **Exploratory Endpoints**   1. Change in lipid levels (total cholesterol, LDL-C, HDL-C, triglycerides) at 6, 12, 18, and 24 weeks compared to baseline 2. Change in liver function markers (AST, ALT, γ-GTP) at 6, 12, 18, and 24 weeks compared to baseline 3. Change in renal function markers (e-GFR, UACR (Urine Albumin to Creatinine Ratio), UGCR (Urine Glucose to Creatinine Ratio)) at 6, 12, 18, and 24 weeks compared to baseline 4. Change in body composition analyzer index (skeletal muscle mass, body fat mass, body fat percentage, muscle mass, waist/hip circumference, body water, intracellular water, extracellular water, extracellular water ratio, abdominal fat percentage, etc.) at 12 and 24 weeks compared to baseline 5. Change in cardiovascular disease complication risk (Atherosclerotic Cardiovascular Disease risk, ASCVD risk) at 12 and 24 weeks compared to baseline |
| **Safety Endpoints** | 1. Laboratory tests, vital signs, physical examination results |
| **Statistical Analysis Method** | **General Principles**  Continuous variables will be presented as descriptive statistics (number of subjects, mean, standard deviation, median, minimum, maximum), and categorical variables will be presented as frequencies and percentages. Unless otherwise specified, all tests will be two-sided with a significance level of 5%. All p-values will be reported to a maximum of three decimal places. If not exactly divisible, values will be rounded to two decimal places, rounding up at the third decimal place.  **Efficacy Endpoints**  For primary and secondary efficacy endpoints, descriptive statistics will be presented at baseline, 6 weeks, 12 weeks, 18 weeks, and 24 weeks. Changes from baseline at 24 weeks or at 6, 12, and 18 weeks will be analyzed using the Independent sample t-test, Mann Whitney U test, or Wilcoxon sum rank test.  **Exploratory endpoint**  For exploratory endpoints, descriptive statistics will be presented at baseline, 6 weeks, 12 weeks, 18 weeks, and 24 weeks. Changes from baseline at 24 weeks or at 6, 12, and 18 weeks will be analyzed using the Independent sample t-test, Mann Whitney U test, or Wilcoxon sum rank test. |

**Schedule for Data Collection in Clinical Study**

| **Collection  Schedule**  **Collection Items** | **Baseline** | **Follow-Up Visits  (Option, ☎)** | **Follow-Up Visits** | **Follow-Up Visits  (Option, ☎)** | **Close-Out Visit (COV)** |
| --- | --- | --- | --- | --- | --- |
|  | Visit 1 | Visit 2 | Visit 3 | Visit 4 | Visit 5 |
|  | 0 day | Week 6  (±2 Weeks) | Week 12 (±2 Weeks) | Week 18 (±2 Weeks) | Week 24 (±2 Weeks) |
| Obtain Written Consent^1)^ | ○ |  |  |  |  |
| Inclusion/Exclusion Criteria Confirmation | ○ |  |  |  |  |
| Assignment of Randomization Number | ○ |  |  |  |  |
| Installation of Well Check and Link to Institution | ○ |  |  |  |  |
| Demographic Information^2)^ | ○ |  |  |  |  |
| Information on Type 2 Diabetes^3)^ | ○ |  |  |  |  |
| Medical History Survey^4)^ | ○ |  |  |  |  |
| Alcohol/Smoking/Lifestyle Habits^5)^ | ○ | (○) | ○ | (○) | ○ |
| Physical Measurement^6^ | ○ | (○) | ○ | (○) | ○ |
| Vital Signs^7)^ | ○ | (○) | ○ | (○) | ○ |
| Body Composition Analysis (InBody) Test ^8)^ | (○) | (○) | (○) | (○) | (○) |
| Prior/Concomitant Medications^9)^ | ○ | (○) | ○ | (○) | ○ |
| Laboratory Tests^10)^ | ○ | (○) | ○ | (○) | ○ |
| Frequency of Well Check Use (Number of Login Days) |  |  |  |  | ○ |
| Well Check Usage Satisfaction Survey^11)^ |  |  |  |  | ○ |

*There are no additional visits or laboratory tests conducted specifically for this clinical study. In a routine clinical setting, it is recommended to collect data at 6-week intervals from Visit 1 (Baseline, Day 0) to 24 weeks (Visit 2: 6 weeks, Visit 3: 12 weeks, Visit 4: 18 weeks, Visit 5: 24 weeks). Visit 2 (6 weeks) and Visit 4 (18 weeks) can be optional visits and data can be collected via phone calls, based on the investigator (responsible physician )'s discretion. If a subject withdraws or drops out from the study, the data collected up to that point should be recorded in the case report form to the maximum extent possible.

1. Written consent must be obtained before performing any clinical study procedures. The date of written consent and Visit 1 (Baseline, Day 0) may differ, but consent must be obtained before participation in the clinical study.
2. Collect demographic information [initials, gender, age (month and year of birth), pregnancy and breastfeeding status].
3. Collect information on Type 2 Diabetes (date of diagnosis).
4. Collect medical history within 6 months prior to Visit 1 (Baseline, Day 0).
5. For alcohol/smoking/lifestyle habits, data is collected at each visit. For the test group, this can be done through the "pre-visit questionnaire" function within "Well Check" or during consultation with the investigator (responsible physician). For the control group, data is collected through consultation with the investigator (responsible physician e) during visits. Alcohol history includes the frequency of drinking per week and the amount consumed per occasion. Smoking history includes selecting one of the following: current smoker, former smoker, or non-smoker. Lifestyle habits include dietary and exercise habits. Dietary habits can include irregular meals, overeating, excessive intake of carbohydrates/sugars, fats, or salt and multiple items can be selected. Exercise habits include the frequency of exercise per week, type of exercise (walking, aerobic exercise, strength training), and intensity of exercise (less than 30 minutes, less than 1 hour, long duration).
6. Collect physical measurements (height, weight, BMI), with BMI calculated automatically through EDC using height and weight. Height is collected only at Visit 1 (Baseline, Day 0) to the first decimal place.
7. After at least 5 minutes of rest, collect vital signs (systolic blood pressure, diastolic blood pressure, pulse rate). If there are measurement results at each visit, collect these results.
8. Body composition analysis (skeletal muscle mass, body fat mass, body fat percentage, muscle mass, waist/hip circumference, body water, intracellular water, extracellular water, extracellular water ratio, abdominal fat percentage) is conducted at Visit 1 (Baseline, Day 0), Visit 3 (Follow-up visit, 12 weeks), and Visit 5 (Close-out visit, 24 weeks). This variable is measured only in institutions where such measurement is possible and is considered an exploratory clinical variable.
9. - Prior Medications: Collect information on diabetes medications used specifically for the treatment of Type 2 Diabetes within 4 weeks prior to Visit 1 (Baseline, Day 0). This includes the drug name (generic or brand name), duration of administration (start date, end date, ongoing status), single dose amount/unit, daily administration frequency, and route of administration.

- Concomitant medications: During the study period after Visit 1 (Baseline, Day 0), collect information on all concomitant medications taken consistently for more than 3 months, including medications for the treatment of Type 2 Diabetes. This includes the drug name (generic or brand name), reason for administration, duration of administration (start date, end date, ongoing status), single dose amount/unit, daily administration frequency, route of administration, any dosage changes or discontinuation, and reasons for changes or discontinuation.

1. Laboratory tests are collected if conducted as part of routine clinical practice. If there are HbA1c results within 4 weeks prior to Visit 1 (Baseline, Day 0) or other test results within 3 months prior to Visit 1, these results can be used in place of the laboratory tests at Visit 1 (Baseline, Day 0). Cardiovascular disease complication risk (ASCVD risk) will be assessed using the ASCVD 2013 Risk Calculator from AHA/ACC. Analysis will be conducted after the study is completed based on information collected in the eCRF (gender, age, systolic blood pressure, total cholesterol, HDL-C, hypertension medication use, diabetes diagnosis, and smoking status). In the test group, the automatic calculation feature within the Well Check app allows investigators (responsible physicians) to input cholesterol and blood pressure values for use during patient consultations.

| Main Laboratory Tests | HbA1c, FPG(FBS) |
| --- | --- |
| Other Laboratory Tests  (Blood Tests) | Total Cholesterol, HDL-C, LDL-C, Triglyceride, ALT, AST, γ-GTP, Creatinine, e GFR, UACR, UGCR |

1. At the close-out visit, a written survey will be conducted to assess the experiences of both the medical staff (responsible physicians) and the subjects with using Well Check.

**Table of Contents**

[1. Introduction 13](#_Toc170399142)

[1.1 Background and Rationale for the Study 13](#_Toc170399143)

[2. Objective of Clinical Study 15](#_Toc170399144)

[2.1 Primary Objectives 15](#_Toc170399145)

[2.2 Secondary Objectives 15](#_Toc170399146)

[2.3 Exploratory Objectives (Exploratory Evaluation) 15](#_Toc170399147)

[2.4 Safety Assessment 16](#_Toc170399148)

[3. Selection of Subjects 17](#_Toc170399149)

[3.1 Number of Subjects 17](#_Toc170399150)

[3.2 Rationale 17](#_Toc170399151)

[3.3 Inclusion Criteria 18](#_Toc170399152)

[3.4 Exclusion Criteria 18](#_Toc170399153)

[3.5 Study Completion and Withdrawal/Dropout 18](#_Toc170399154)

[3.6 Study Termination 19](#_Toc170399155)

[4. Clinical Study Method 20](#_Toc170399156)

[4.1 Overall Study Design 20](#_Toc170399157)

[4.2 Duration of Study 21](#_Toc170399158)

[4.3 Study Procedure and Schedule 21](#_Toc170399159)

[Visit Schedule 21](#_Toc170399160)

[4.4 Information and Items Collected 22](#_Toc170399161)

[4.5 Randomization 26](#_Toc170399162)

[5. Investigational Software 27](#_Toc170399163)

[5.1 Information on the Investigational Software 27](#_Toc170399164)

[5.2 Use of Investigational Software 28](#_Toc170399165)

[6. Data Management 29](#_Toc170399166)

[6.1 Source Documents 29](#_Toc170399167)

[6.2 Data Entry 29](#_Toc170399168)

[6.3 Data Verification 29](#_Toc170399169)

[6.4 Data Storage 29](#_Toc170399170)

[6.5 Access to Data 30](#_Toc170399171)

[7. Evaluation Criteria and Methods, and Statistical Analysis Methods 30](#_Toc170399172)

[7.1 Endpoints 30](#_Toc170399173)

[7.2 Statistical Analysis Method 31](#_Toc170399174)

[Efficacy Analysis Set 31](#_Toc170399175)

[Safety Analysis Set 31](#_Toc170399176)

[Primary Efficacy Endpoints 31](#_Toc170399177)

[Secondary Efficacy Endpoints 31](#_Toc170399178)

[Exploratory Endpoints 31](#_Toc170399179)

[Vital Signs 32](#_Toc170399180)

[Laboratory Tests 32](#_Toc170399181)

[8. Ethical Considerations and Administrative Procedures 33](#_Toc170399182)

[8.1 Ethics Committee/Institutional Review Board (IRB) 33](#_Toc170399183)

[8.2 Ethical Considerations 33](#_Toc170399184)

[8.3 Quality Assurance and Audit 33](#_Toc170399185)

[8.4 Subject Informed Consent 33](#_Toc170399186)

[8.5 Approval and Amendment of the Protocol 34](#_Toc170399187)

[8.6 Monitoring of Clinical Study Sites 34](#_Toc170399188)

[8.7 Confidentiality and Privacy Protection for Subjects 34](#_Toc170399189)

[8.8 Measures to Protect the Safety of Subjects 35](#_Toc170399190)

[8.9 Utilization and Publication of Study Results 35](#_Toc170399191)

[9. Principal Investigator 36](#_Toc170399192)

[9.1 Information of Principal Investigator 36](#_Toc170399193)

[9.2 Roles and Responsibilities of the Principal Investigator 36](#_Toc170399194)

[10. Appendices 37](#_Toc170399195)

[11. References 38](#_Toc170399196)

**List of Abbreviations**

| ALT | Alanine Aminotransferase |
| --- | --- |
| ASCVD | Atherosclerotic cardiovascular disease |
| AST | Aspartate Aminotransferase |
| BMI | Body Mass Index |
| CFR | Code of Federal Regulations |
| CS | Clinically Significant |
| DPP-4 | dipeptidyl peptidase 4 |
| eCRF | electronic Case Report Form |
| EDC | Electronic Data Capture |
| FPG | Fasting Plasma Glucose |
| GCP | Good Clinical Practice |
| GLP-1 | Glucagon-like Peptide-1 |
| γ-GTP | Gamma glutamyl transferase |
| HbA1c | Hemoglobin A_1c_ |
| HDL-C | High Density Lipoprotein Cholesterol |
| ICH | International Council for Harmonisation |
| IEC | Independent Ethics Committee |
| IRB | Institutional Review Board |
| LDL-C | Low Density Lipoprotein Cholesterol |
| NCS | Not Clinically Significant |
| SE | Standard Error |
| SOP | Standard Operating Procedures |
| TC | Total Cholesterol |
| TG | Triglyceride |
| UACR | Urine Albumin to Creatinine Ratio |
| UGCR | Urine Glucose to Creatinine Ratio |
|  |  |
|  |  |
|  |  |

# Introduction

## Background and Rationale for the Study

Diabetes mellitus, obesity, and dyslipidemia are important metabolic disorders, as well as the significant risk factors associated with cardiovascular diseases such as heart attack, stroke, and hypertension. Diabetes, in particular, is a severe metabolic disorder characterized by hyperglycemia (elevated blood sugar levels) due to insulin dysfunction caused by genetic and environmental factors. This condition prevents glucose in the blood from being delivered to or stored in cells, resulting in excessively high blood sugar levels. According to the International Diabetes Federation (IDF), the global number of diabetes patients increased from 425 million in 2017 to 463 million in 2019 and is projected to rise explosively to 642 million by 2040, accounting for over 10% of the global adult population [1]. In South Korea, the number of diabetes patients has been rapidly increasing due to complex factors such as Westernized dietary habits, lack of exercise, and increased lifespan resulting from economic growth and higher income levels. The number of diabetes patients increased from 3 million in 2007 to 3.51 million in 2010 and is estimated to reach 5.4 million (Statistics Korea, 10.85% of the total population) by 2030.

Diabetes is broadly classified into Type 1 Diabetes (T1D), Type 2 Diabetes (T2D), and Gestational Diabetes. Among these, Type 2 Diabetes is the most common, accounting for over 90% of diabetes cases worldwide. It initially occurs when the body's cells do not fully respond to insulin, leading to insulin resistance and increased insulin production at inappropriate times [2]. As the disease progresses, inadequate insulin production can occur when pancreatic beta cells fail to meet demand

Generally, patients newly diagnosed with Type 2 Diabetes are advised to make aggressive lifestyle changes initially. If these changes alone do not achieve the target HbA1c levels, medication treatment is initiated [3]. Current oral diabetes medications can be broadly classified into insulin preparations, sulfonylureas (SU), thiazolidinediones (TZD), biguanides, α-glucosidase inhibitors, meglitinides, incretin mimetics, and DPP-4 inhibitors, etc.

The treatment of diabetes generally follows guidelines from the American Diabetes Association (ADA), with therapy tailored based on the presence of cardiovascular diseases and the progression of diabetes, employing monotherapy or combination therapy. The ADA currently recommends that most adults with diabetes achieve an HbA1c level below 7.0%, blood pressure below 140/90 mmHg (below 130/90 mmHg for those at high risk of cardiovascular disease), and low-density lipoprotein cholesterol (LDL-C) below 100 mg/dL [4]. Additionally, South Korean diabetes treatment guidelines suggest an HbA1c target below 6.5% for Type 2 Diabetes patients.

According to the United Kingdom Prospective Diabetes Study (UKPDS), a 1% reduction in HbA1c in Type 2 Diabetes patients decreased microvascular complications by 37% and myocardial infarction by 14%. Although there are slight variations between studies, aggressive and strict blood sugar control has been reported as the most effective method to prevent diabetes complications and slow the progression of already occurred complications.

Moreover, all patients diagnosed with Type 2 Diabetes should receive education and take action to improve their lifestyle upon diagnosis. The goal of diabetes self-management education is to improve diabetes management, reduce complications and mortality, and ultimately enhance the quality of life for patients. It helps patients acquire the knowledge and methods necessary to plan healthy meals, engage in regular physical activity, make effective self-management behavioral changes, and cope with various situations. Randomized controlled clinical trials on Type 2 Diabetes patients have shown that self-management and education improve the ability to handle health-related issues, enhance self-management behaviors, and increase self-efficacy and empowerment, thereby improving the quality of diabetes management. Effective self-management and psychological well-being are foundational for achieving diabetes treatment goals.

Patients must start effective self-management education upon the initial diagnosis of diabetes. As diabetes is a chronic disease requiring continuous management, self-management education should be supplemented and sustained whenever the patient's situation changes. To maintain healthy lifestyle habits, at least six months of management is needed. Meta-analysis results have shown that self-management education conducted for more than 10 hours over 6-12 months improves blood sugar control and reduces mortality. Therefore, diabetes self-management and education are effective when tailored to the patient's age, personal needs, and preferences, especially when treatment goals are not met, or life cycle changes or complications occur.

Interactive and continuous processes defined as diabetes-related education and support have proven successful in helping patients develop the knowledge, skills, and abilities needed for successful self-management [4]. As technology advances, various forms of digital health coaching, such as mobile phone messages, mobile applications, and web-based algorithms, have been reported to improve blood sugar control and enhance the self-management capabilities of diabetes patients. Combined with education or counseling, self-monitoring can help manage blood pressure and improve medication adherence in hypertension patients [5].

The use of information and communication technology in the medical field is rapidly expanding globally. [6]. Digital medical technology is recognized for improving accessibility, effectiveness, and quality of life by bridging the gap between validated medical services and patient self-management [7]. Despite this, the results have been inconsistent. While two reviews indicated that telemedicine effectively lowers blood pressure, another review did not consistently demonstrate these results [8,9]. Digital healthcare smartphone applications have been widely used to manage chronic diseases such as diabetes and hypertension, offering promising prospects for improving healthcare service delivery, increasing patient engagement, and enhancing self-management. However, there is still a lack of conclusive evidence on the impact of integrating these applications into hospital systems for managing these diseases [10].

Therefore, this clinical study aims to confirm the actual effectiveness of using the hospital-linked digital healthcare smartphone application 'Well Check' to lower blood sugar, control blood pressure, and manage weight in adult patients with Type 2 Diabetes under routine care. It also seeks to evaluate additional efficacy and safety information and obtain real-world usage data.

# Objective of Clinical Study

This clinical study aims to confirm the improvement effect of clinical outcomes in Type 2 Diabetes patients using ‘Well Check’ for 24 weeks in a real clinical setting, with or without investigator-managed linkage.

## Primary Objectives

Percent change and change of HbA1c at 24 weeks compared to baseline.

## Secondary Objectives

1. Percent change and change in FPG at 6, 12, 18, and 24 weeks compared to baseline
2. Percent change and change in HbA1c at 6, 12, and 18 weeks compared to baseline
3. Proportion of subjects achieving HbA1c < 7% at 6, 12, 18, and 24 weeks compared to baseline
4. Proportion of subjects achieving HbA1c < 6.5% at 6, 12, 18, and 24 weeks compared to baseline
5. Proportion of subjects achieving therapeutic response [change in HbA1c (baseline HbA1c – HbA1c at each evaluation time point) > 0.5% or HbA1c < 7%] at 12 and 24 weeks compared to baseline
6. Change in blood pressure (systolic and diastolic) at 6, 12, 18, and 24 weeks compared to baseline
7. Change in weight and BMI at 6, 12, 18, and 24 weeks compared to baseline
8. Proportion of subjects with a reduction of 5% or more in weight and BMI at 6, 12, 18, and 24 weeks compared to baseline

## Exploratory Objectives (Exploratory Evaluation)

1. Change in lipid levels (total cholesterol, LDL-C, HDL-C, triglycerides) at 6, 12, 18, and 24 weeks compared to baseline
2. Change in liver function markers (AST, ALT, γ-GTP) at 6, 12, 18, and 24 weeks compared to baseline
3. Change in renal function markers [e-CFR, UACR, UGC (Urine Albumin to Creatinine Ratio), UGCR (Urine Glucose to Creatinine Ratio)] at 6, 12, 18, and 24 weeks compared to baseline
4. Change in body composition analyzer indicators (skeletal muscle mass, body fat mass, body fat percentage, muscle mass, waist/hip circumference, body water, intracellular water, extracellular water, extracellular water ratio, abdominal fat percentage) at 12 and 24 weeks compared to baseline
5. Change in cardiovascular disease complication risk (ASCVD risk) at 12 and 24 weeks compared to baseline

## Safety Assessment

1. Laboratory tests, vital signs, physical examination results

# Selection of Subjects

## Number of Subjects

At least 480 subjects (minimum number to ensure statistical significance considering about 20% dropouts rate)

## Rationale

Based on the pilot study results, the difference in average HbA1c between the test group and the control group after 24 weeks of using Well Check in Type 2 Diabetes patients was considered to be 0.4% (8.0% vs. 7.6%), with a standard deviation (*s*) of 1.1%. Referring to these results, the total sample size (*n*) required was estimated to be approximately 274 subjects using G*Power 3.1.9.7.

Since this study employs cluster randomization, we further adjusted the sample size calculation by incorporating the design effect to account for potential intra-cluster correlations among patients within the same clinic. We calculated the design effect using the standard formula:

$$Design effect=1+\left( m-1 \right)\times ICC$$

Referring to previous studies conducted in primary care settings for patients with type 2 diabetes [11, 12], we conservatively adopted an intraclass correlation coefficient (ICC) value of 0.02 and assumed an average cluster size (*m*) of 20 participants per center. Therefore, we calculated the resulting design effect to be 1.38, and applying this adjustment to the initial sample size estimate gives a revised total sample size of 378 participants. Finally, considering an anticipated dropout rate of approximately 20%, we adjusted the target recruitment number further to approximately 480 participants.


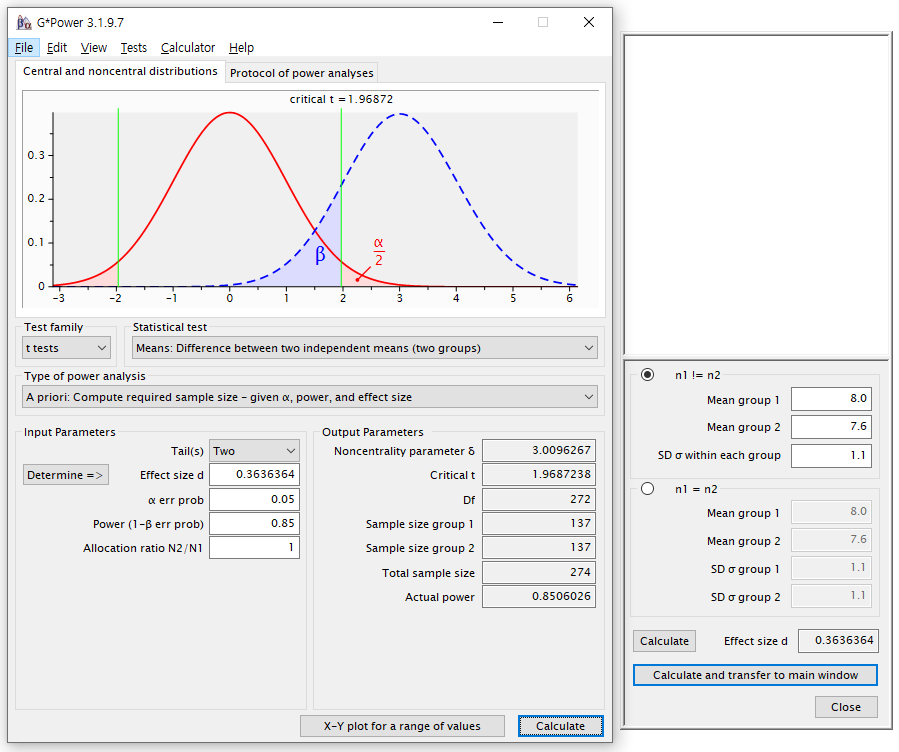


< Calculation for Subject Number – Based on Glycated hemoglobin>

## Inclusion Criteria

Select individuals who meet all of the following criteria:

1. Adults aged 19 to 80 years
2. Patients with Type 2 Diabetes who are currently receiving or are scheduled to receive Envlo Tab. or Envlomet SR Tab. based on the approved conditions
3. Individuals who have no difficulty using a digital healthcare smartphone application on their smartphone
4. Individuals who plan to engage in appropriate exercise and dietary therapy for blood glucose control during the clinical study period
5. Women of childbearing potential and men who agree to use appropriate contraceptive methods or have no plans for pregnancy during the observational study period

*Hormonal contraceptives, intrauterine device (IUD) or intrauterine system (IUS) implants, vasectomy, tubal ligation, double barrier methods (simultaneous use of cervical cap or diaphragm with male condom), etc.

1. Individuals who have listened to and understood the detailed explanation of the clinical study, and have voluntarily signed a written informed consent agreeing to participate in the study and to comply with subject precautions during the study period

## Exclusion Criteria

Individuals who meet any of the following exclusion criteria will be excluded from this clinical study

1. Individuals with diabetes other than Type 2 Diabetes (e.g., Type 1 Diabetes, Diabetic Ketoacidosis, Gestational Diabetes, etc.)
2. Individuals who fall under the contraindications for administration according to the approved conditions of Envlo Tab. or Envlomet SR Tab.

- Patients who have hypersensitivity or a history of hypersensitivity to any component of Envlo Tab. or Envlomet SR Tab.
- Individuals with an estimated Glomerular Filtration Rate (eGFR) less than 30 mL/min/1.73m²
- Patients with renal impairment with an eGFR less than 60 mL/min/1.73m², end-stage renal disease, or those on dialysis
- Individuals with moderate to severe hepatic impairment (AST or ALT > 3 times the upper limit of normal, Total Bilirubin > 2 times the upper limit of normal, hepatitis, or liver failure)
- Individuals classified as class III or IV according to the New York Heart Association (NYHA) classification

1. Patients who have undergone treatment with obesity medications or weight loss drugs, or other treatments (surgery, diet, etc.) leading to unstable weight within 3 months prior to the enrollment date
2. Individuals with mental incapacity
3. Pregnant or breast-feeding women
4. Individuals participating in another clinical trial and receiving investigational drugs or investigational medical devices
5. Individuals who have prior experience using the Well Check application before study enrollment
6. Individuals deemed unsuitable for participation in this clinical study based on the judgment of the investigator (responsible physician)

## Study Completion and Withdrawal/Dropout

1. **Subject’s Completion and Termination of Study Participation**

A subject is considered to have completed the study upon completing the follow-up visit procedures at the 24-week point after their first visit (baseline). The study is deemed complete when the last subject finishes their study participation.

1. **Subject’s Withdrawal/Dropout of Study Participation**

Subjects can discontinue their participation in the study at any time at their discretion. The investigator or sponsor can also withdraw/drop a subject’s participation at any time for safety, behavioral, or administrative reasons. The following are conditions under which a subject might be withdrawn/dropped from the study, and the investigator shall enter the collected study data into the eCRF up to the point of withdrawal/dropout:

1. A subject not meeting the inclusion/exclusion criteria was discovered during the clinical study
2. Withdrawal by subject (or his/her legal representative)
3. Non-Compliance with Investigational Product

: Subjects do not agree to or follow the investigator’s instructions related to the investigational software.

1. Lost to follow-up

: Subjects cannot be contacted for follow-up.

1. Physician Decision

If a subject is withdrawn/drops out, the investigator must notify the sponsor and make every effort to complete the end-of-study evaluation. The reasons for withdrawal/dropout must be documented in the source document and the eCRF.

## Study Termination

The principal investigator and the investigator (IRB-approved and delegated by the principal investigator) may, in consultation with the study sponsor, decide to terminate early or temporarily suspend the clinical study if it is deemed undesirable to continue based on the results collected during the study process. The sponsor may also terminate the study early for safety or administrative reasons. The principal investigator must promptly report this to the IRB and provide detailed reasons for the early termination or suspension.

Reasons for study termination by the sponsor are as follows:

1. Cancellation or suspension of the marketing authorization of the investigational product.
2. The sponsor determines that continuing the study is not medically or ethically justified.
3. Excessive delays in subject recruitment make the study unfeasible.

In the event of early termination or temporary suspension, the investigator must inform the subjects and ensure appropriate actions and follow-up visits are carried out. The investigator must summarize and report the status and results up to the point of termination in the eCRF and provide this information to the sponsor.

# Clinical Study Method

## Overall Study Design

This clinical study is designed as a primary care-based, prospective, multicenter, cluster-randomized, pragmatic clinical trial to confirm the improvement effects on blood glucose, blood pressure, and weight over 24 weeks in patients with Type 2 Diabetes when using ‘Well Check’ with or without investigator-linked management in real-world primary care settings.

Eligible subjects include patients currently being treated with Envlo Tab. or Envlomet SR Tab. for Type 2 Diabetes or those deemed eligible for such treatment by the investigator (responsible physician). Subjects must also be able to use the digital healthcare smartphone application without difficulty.

Due to the practical constraints of primary care settings, where it is challenging to manage test group and control groups separately within the same institution, cluster randomization will be used. Institutions will be randomized as either the test group [institutions managing subjects with the 'Well Check' app in conjunction with the investigator (responsible physician)] or the control group (institutions where subjects use the 'Well Check' app for self-management). The study will be conducted following this setup.


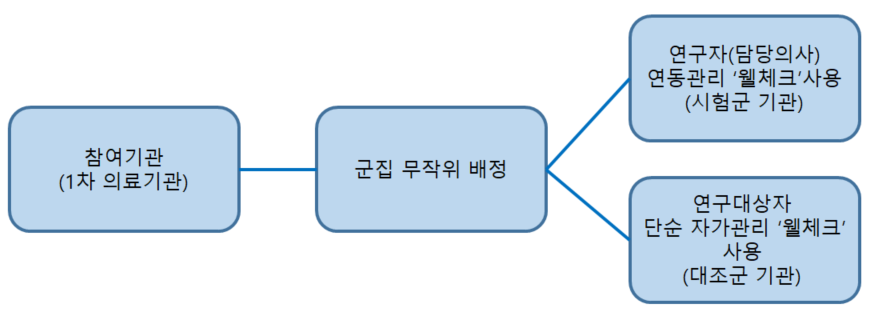


Study Subjects

Use of 'Well Check'
for Simple Self-Management

(Control Group Institutions)

Investigator
(Responsible Physician)

Use of 'Well Check'
for Integrated Management
(Study Group Institutions)

Cluster Randomization

Participating Institutions

(Primary Care Institutions)

Figure 1. Clinical Study Design

Data collection for this clinical study will be based on medical records documented during routine clinical visits. Collected data will include demographic information, physical measurements, vital signs, and laboratory test results. There are no mandatory visits, tests, or treatments specified by this study.

However, prospective data collection through follow-up visits will be conducted up to 24 weeks from the baseline registration date.

The planned time points for prospective data collection are baseline (0 days), visit 2 (6 weeks), visit 3 (12 weeks), visit 4 (18 weeks), and visit 5 (24 weeks). Visits 2 and 4 can be optionally collected based on the investigator (responsible physician)'s judgment and allow for telephone follow-ups.


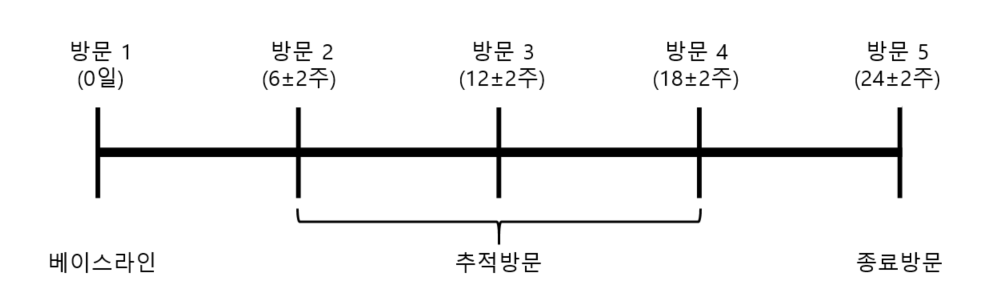


Visit 1

(0 days)

Visit 2

(6±2 weeks)

Visit 3

(12±2 weeks)

Visit 4

(18±2 weeks)

Visit 5

(24±2 weeks)

Baseline

Follow-Up Visits

Close-Out Visit (COV)

Figure 2. Clinical Study Flowchart

## Duration of Study

This study is expected to last till December 31, 2026 from the date of IRB approval. For each subject, the data collection period will be up to 24 weeks (6 months), during which the information specified in the study protocol will be collected. However, if circumstances arise that affect the study progress, such as difficulties in subject recruitment, the period may vary.

## Study Procedure and Schedule

### Visit Schedule

There are no mandatory visits, tests, or treatments specified by this study. Data will be prospectively collected using medical records, investigator assessments, and laboratory test results from routine clinical visits. Each investigator will collect predefined information from subjects at baseline and during the follow-up period.

1. **Visit 1 (Baseline, Day 0)**

At the first visit (baseline), the following information will be collected:

- Obtain subject consent and assign subject enrollment number
- Inclusion/Exclusion Criteria Confirmation
- Installation and Hospital Integration of the Digital Healthcare Smartphone Application ‘Well Check’
- Demographic information [initials, gender, age (month and year of birth), pregnancy and breastfeeding status].
- Information on Type 2 Diabetes (Diagnosis date)
- Medical history (Past medical history within 6 months prior to enrollment (excluding the target disease) and current medical history)
- Alcohol/Smoking/Lifestyle information
- Physical measurements (height, weight, BMI)
- Vital signs (systolic/diastolic blood pressure, pulse rate)
- Body composition analysis (include skeletal muscle mass, body fat mass, body fat percentage, muscle mass, waist/hip circumference, body water, intracellular water, extracellular water, extracellular water ratio, abdominal fat percentage, etc.)
- Prior medications (diabetes treatments within 4 weeks prior to enrollment) and concomitant medications (all medications, including diabetes treatments, taken at the time of enrollment).
- Laboratory tests (HbA1c, FPG, Total Cholesterol, LDL-C, HDL-C, Triglyceride, ALT, AST, Creatinine, e GFR, UACR, UGCR, etc.)

1. **Follow-Up Visits**

Follow-up visits will be conducted at 6-week intervals from the first visit (baseline) as Visit 2 (6 weeks ±2 weeks), Visit 3 (12 weeks ±2 weeks), Visit 4 (18 weeks ±2 weeks), and Visit 5 (24 weeks ±2 weeks). Visits 2 and 4 may be optional and data collection can be conducted via phone calls, based on the investigator (responsible physician)’s discretion. During follow-up visits, the following information will be collected:

- Alcohol/Smoking/Lifestyle information
- Physical measurements (weight, BMI)
- Vital signs (systolic/diastolic blood pressure, pulse rate)
- Body composition analysis (include skeletal muscle mass, body fat mass, body fat percentage, muscle mass, waist/hip circumference, body water, intracellular water, extracellular water, extracellular water ratio, abdominal fat percentage, etc.)
- Concomitant medications
- Laboratory tests (HbA1c, FPG, Total Cholesterol, LDL-C, HDL-C, Triglyceride, ALT, AST, Creatinine, e GFR, UACR, UGCR, etc.)
- (End of Study) Well Check User Experience Satisfaction Survey

## Information and Items Collected

1. **Obtaining Written Consent and Assigning Screening Numbers to Study Subjects**

Study subjects are outpatient visitors, and, if necessary, subjects are recruited through voluntary participation via recruitment announcements on the hospital's bulletin board.

Before collecting data related to this study, the investigator must explain the purpose and details of the study to the study subjects through an information sheet and obtain voluntary consent. This consent must include the subject's name, signature, and the signing date.

The date of written consent and Visit 1 (Baseline, Day 0) may differ, but consent must be obtained before participation in the clinical study.

Screening numbers for subjects are automatically assigned in the eCRF upon obtaining consent. Subject numbers are 6 digits and formatted as “AXX-ZZZ”, where:

- “A”: Classification code (A, B)
- “XX”: Institution number (01, 02 -)
- “ZZZ”: Subject number at each institution in the order of consent (001, 002 -)

1. **Inclusion/Exclusion Criteria Confirmation**

Inclusion/Exclusion criteria are confirmed at Visit 1 (Baseline, Day 0) to verify if all inclusion criteria are met and none of the exclusion criteria apply.

1. Installation and Registration of the ‘Well Check’ APP

Subjects enrolled in the study will install the 'Well Check' smartphone application, register, and enter the institution code provided for the study to link it with the hospital.

**[Test Group]**

For the test group, the basic usage of the 'Well Check' smartphone application is recommended as follows. Within the 'Well Check' app, investigators and subjects are encouraged to freely use the features considering the subject's medical condition. If self-recording for chronic disease management such as [Blood Glucose], [Blood Pressure], [Weight], [Medication Records], or regular completion of [Health Education] in 'Well Check' is insufficient, the investigator can encourage self-management and education completion via the messaging feature of 'Well Check.'

1. Visit 1 (Baseline, Day 0)

- Subject: Complete [Basic Questionnaire], [Health Checkup Results], and set [Medication Alarm] in the 'Well Check' application.
- Investigator (Responsible Physician): Enter [Blood Test Results], evaluate [ASCVD risk], and use the linked subject's medical history, lifestyle, and symptoms for consultation in the 'Well Check Doctor Web.'
- See Appendix 6. Provide and guide the “Attachment 6. Well Check User Guide” pamphlet.

1. Visit 2 (Follow-up Visit, 6 weeks) to Visit 5 (Close-out Visit, 24 weeks)

- Subject: Enter [Pre-visit Questionnaire] in the 'Well Check' application during visits.
- Investigator (Responsible Physician): Review and analyze the patient’s self-records (questionnaire, blood glucose, blood pressure, weight, medication records) and recent symptoms and lifestyle (alcohol/smoking/exercise) feedback in the 'Well Check Doctor Web.' Enter [Blood Test Results] (if tested), evaluate [ASCVD risk], and use it during consultations

1. During the Study Period (Visit 1 to Visit 5)

- Subject: Record [Blood Glucose] (at least once daily), [Medication Records] (when taking medication), [Blood Pressure] and [Weight] (at least once weekly) in the 'Well Check' application. Complete [Primary Doctor Messages] education (twice weekly) and use [Health Education].
- Investigator (Responsible Physician): Monitor cautionary patients* in 'Well Check Doctor Web' (once weekly) and send encouragement messages to those who do not measure blood glucose (once monthly)

**An alert is sent to 'Well Check Doctor Web' if the blood glucose level is below 54 mg/dL or above 200 mg/dL*

- Automatic dispatch of subject educational materials (twice weekly)

**[Control Group]**

For the control group, the basic usage of the 'Well Check' smartphone application is recommended as follows. Subjects are encouraged to use the 'Well Check' app in a self-recording format. The investigator will not monitor or utilize the subject's use of 'Well Check' during the study period.

1. Visit 1 (Baseline, Day 0) to Visit 5 (Close-out Visit, 24 weeks)

- No specific guidance or settings for the 'Well Check' application are provided; subjects use it freely for self-recording
- Investigator (Responsible Physician): No separate management or consultation via 'Well Check Doctor Web'; routine medical care is provided
- Automatic dispatch of subject educational materials (twice weekly)

1. **Demographic Information**

At Visit 1 (Baseline, Day 0), the following information is collected to verify the subject's basic information. Pregnancy status is collected through interview or existing pregnancy test results, if available.

- Initials
- Gender
- Month and year of birth, age
- Pregnancy and breastfeeding status

1. **Information on Type 2 Diabetes**

Collect the diagnosis date of type 2 Diabetes at Visit 1 (Baseline, Day 0).

1. **Medical History Review**

For medical history, any clinically significant medical conditions or abnormalities observed before Visit 1 (Baseline, Day 0), excluding the target disease, will be defined. The diagnosis name and date of each condition will be collected for medical history within 6 months prior to Visit 1 (Baseline, Day 0) and ongoing conditions.

1. **Alcohol/Smoking/Lifestyle Information**

At Visit 1 (Baseline, Day 0), the following information is collected to verify the subject's basic information, and any changes will be additionally collected at each subsequent visit. Verify through routine medical visits, medical records, and for the test group, data collected within the 'Well Check' application can be utilized.

1. Drinking history
   - Current Drinker*: ≥ 12 glasses (units) in a lifetime and drinking at least once in the last 12 months
   - Former Drinker: Has consumed at least 12 units of alcohol in their lifetime but has not drunk in the past 12 months
   - Non-Drinker: < 12 glasses (units) in a lifetime

^*^ For current drinkers, the weekly drinking frequency and amount per occasion will be confirmed.

1. Smoking history
   - Current Smoker: Has smoked ≥ 5 packs (100 cigarettes) in a lifetime and smoked within the past 30 days
   - Former Smoker: Has smoked ≥ 5 packs (100 cigarettes) in a lifetime, but no smoking in the last 30 days
   - Non-smoker: Has smoked < 5 packs (100 cigarettes) in a lifetime
2. Lifestyle Habits (Dietary Habits): Irregular meals, overeating, excessive intake of carbohydrate/sugar, excessive fat intake, excessive salt intake
3. Lifestyle Habits (Exercise Habits): Types of exercise (walking, aerobic exercise, strength training), weekly exercise frequency, exercise intensity (less than 30 minutes, up to 1 hour, long duration)
4. **Physical Measurement**

Collect weight and BMI at every visit, with BMI automatically calculated in the CRF using height and weight. Collect height once at Visit 1 (Baseline, Day 0).

1. **Vital Signs**

At every visit, the following vital signs will be collected:

- Blood pressure (systolic/diastolic)
- Pulse Rate

Blood pressure and pulse rate should be measured in a quiet environment after at least 5 minutes of rest in a chair with a backrest. The subject should refrain from smoking, alcohol, and caffeine intake for at least 30 minutes prior to measurement, if possible.

1. **Body Composition Analysis**

For the feasible institutions capable of conducting these measurements, collect the following items at Visit 1 (Baseline, Day 0), Visit 3 (Follow-up visit, 12 weeks), and Visit 5 (Close-out visit, 24 weeks).

- Body composition analyzer indices: Skeletal muscle mass, body fat mass, body fat percentage, muscle mass, waist/hip circumference, body water, intracellular water, extracellular water, extracellular water ratio, abdominal fat percentage, etc.

1. **Prior/Concomitant Medications**

For prior medications, collect information on diabetes treatments administered within 4 weeks before Visit 1 (Baseline, Day 0). For concomitant medications, collect information on all concomitant medications taken for 3 months or more during the study period after Visit 1 (Baseline, Day 0), including those for the treatment of Type 2 Diabetes.

- Drug Name (Brand Name)
- Dosage and Administration (Dose per administration, unit, frequency, route)
- Duration of Administration (Start date, end date, ongoing status)
- Purpose of Administration
- Reason for Dose Adjustment or Discontinuation (if applicable)

1. **Laboratory Tests**

Laboratory tests are collected based on standard clinical procedures in a real-world clinical setting. If there are HbA1c results within 4 weeks prior to Visit 1 (Baseline, Day 0) or other test results collected within 3 months, these results can be used as substitutes for the laboratory tests at Visit 1 (Baseline, Day 0).

Cardiovascular disease complication risk (Atherosclerotic Cardiovascular Disease risk, ASCVD risk) will be assessed using the ASCVD 2013 Risk Calculator from AHA/ACC. Analysis will be conducted after the study is completed based on information collected in the eCRF (gender, age, systolic blood pressure, total cholesterol, HDL-C, hypertension medication use, diabetes diagnosis, and smoking status). For the test group, the automatic calculation feature within the Well Check app allows investigators (responsible physicians) to input cholesterol and blood pressure values for use during patient consultations.

Examples of Laboratory Test Items to be Collected:

| Main Laboratory Tests | HbA1c, FPG |
| --- | --- |
| Other Laboratory Tests | Total Cholesterol, HDL-C, LDL-C, Triglyceride, ALT, AST, γ-GTP, Creatinine, e GFR, UACR, UGCR |

1. **Well Check Usage Satisfaction Survey**

The satisfaction survey on the usage experience of 'Well Check' by both medical staff and subjects will be conducted in writing during Visit 5 (Close-out Visit, Week 24).

The survey consists of 10 questions and 5 scales designed to evaluate satisfaction with the use of the 'Well Check' application. It is a general survey aimed at assessing the effectiveness of chronic disease management and improvement in work efficiency due to the application usage, rather than targeting any specific disease group.

The 10 questions of Medical Staff Satisfaction Survey aim to assess the effectiveness of managing patients with chronic diseases and the improvement in work efficiency due to the use of the application. The 5-point scale consists of 1) Strongly Disagree 2) Disagree 3) Neutral 4) Agree 5) Strongly Agree

The 10 questions of Subject Satisfaction Survey aim to assess the satisfaction with the relationship with medical staff and the helpfulness of the treatment process experience due to the use of the application. The 5-point scale consists of 1) Strongly Disagree 2) Disagree 3) Neutral 4) Agree 5) Strongly Agree

## Randomization

Due to the practical constraints of primary care settings, where it is challenging to manage test group and control groups separately within the same institution, cluster randomization will be used. Institutions will be randomized as either the test group [institutions managing subjects with the 'Well Check' app in conjunction with the investigator (responsible physician)] or the control group (institutions where subjects use the 'Well Check' app for self-management).

For cluster randomization, participating primary medical institutions will be divided into small groups based on basic characteristics [age of the treating physician (45 years and older, or younger), region (metropolitan, non-metropolitan)]. Within these small groups, the test group and control group will be randomly assigned in a 1:1 ratio using the cluster randomization method.

To ensure strict randomization, an independent person not related to this clinical study will prepare the randomization list using the Proc PLAN procedure of SAS (Ver. 9.4 or higher, SAS Institute, Cary, NC, USA). The generated randomization list will be provided to the IWRS (interactive web response system) developer. The randomization number will be verified through IWRS.

After obtaining written consent for participation in this clinical study, the subject's screening test results will be evaluated. Only those who meet the inclusion criteria and do not fall under the exclusion criteria will be assigned a Randomization Enrollment Number.

The randomization enrollment number will be given out as “AAA-RXX-YYY” and means the following.

- “AAA”: Institution identification number
- “R”: Cluster Randomization
- “XX”: Stratification identifiers [age of the treating physician (45 years and older, or younger), region (metropolitan, non-metropolitan)]

- “YYY”: Order of randomly assigned subjects

|  | Metropolitan (X1) | Non-metropolitan (X2) |
| --- | --- | --- |
| 45 years and older (1X) | 11 | 12 |
| 45 years or younger (2X) | 21 | 22 |

# Investigational Software

## Information on the Investigational Software

**
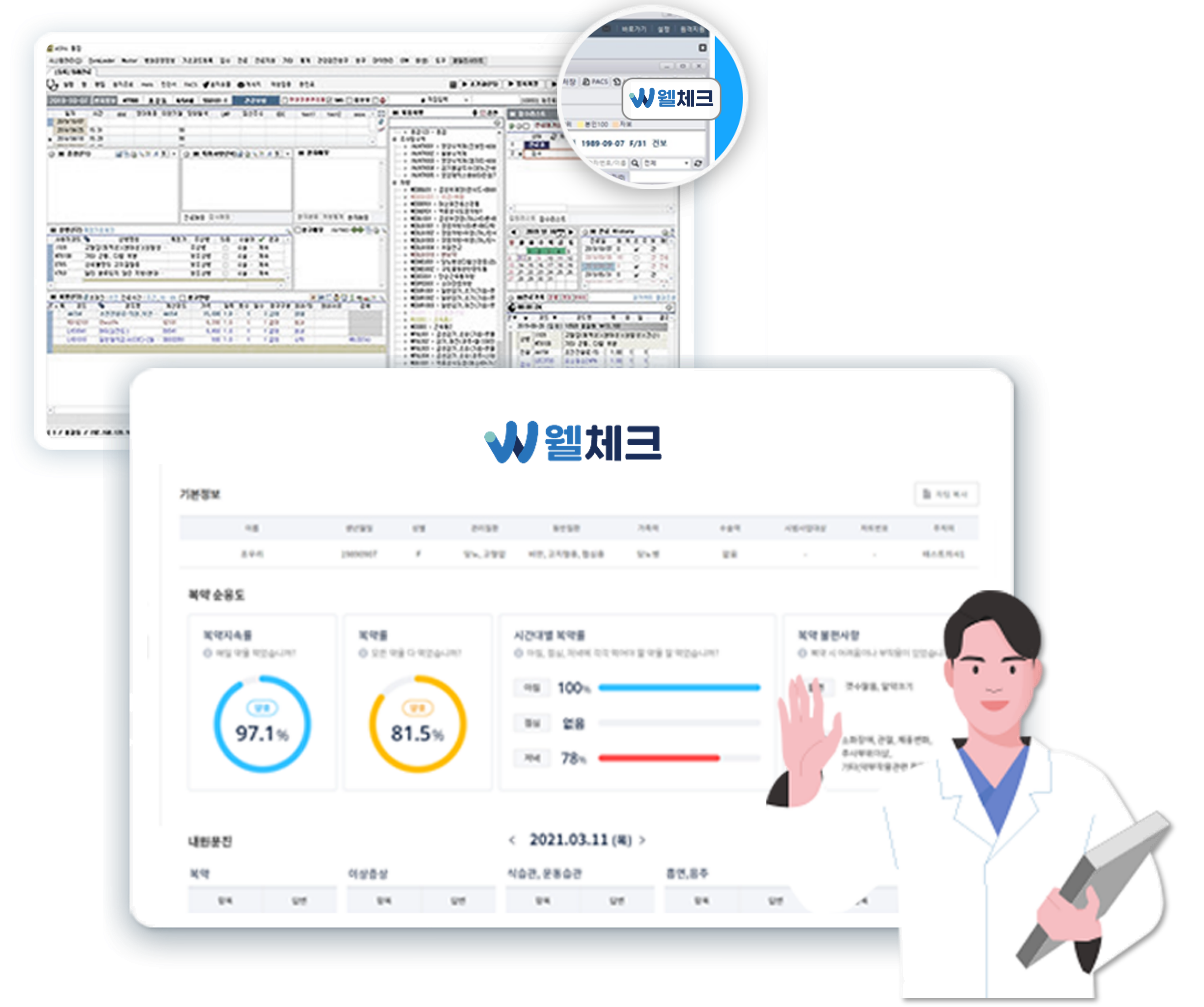
**

The hospital-linked digital health application Well Check differentiates itself from other simple digital health applications by connecting the “Patient Application (App)” with the “Physician Web (Web) and Electronic Medical Record (EMR)” to manage diabetes and hypertension. Physicians can conveniently monitor patients' health statuses and manage them via the “Physician Web.”

Well Check allows users to record blood pressure, blood sugar, and specific health issues through surveys. If blood pressure and blood sugar are not recorded at least once a week, the application sends an alert to the user. The data is then transmitted to the physician for analysis and monitoring. If the recorded blood pressure or blood sugar levels are too high or low compared to the target, the physician can send feedback messages for management based on their judgment. This enables physicians to manage patients and monitor high-risk patients separately. Additionally, users can check how effectively their numbers are managed compared to the target in their preferred format—graphs, lists, or tables. Furthermore, the National Health Insurance Service links the results of health check-ups conducted throughout a patient's lifetime and provides them to the primary care physician, who can then send feedback messages to the patient.

1. **Key Features of the Well Check Application**
2. Integration with Hospital Charts (EMR) – Aegis, C-Chart, BeatA, Sense, WehagoH
3. Analysis of blood pressure, blood sugar, medication, concomitant symptoms, and lifestyle habits, etc.
4. Monitoring high-risk patients, with visit reminders when necessary
5. Sending messages from the primary care physician
6. Integration of health check-up results
7. Recommendations for tests and education
8. Analysis of continuous glucose reports
9. Support for overseas medical care services
10. Obesity management
11. **How to Use the Well Check Application**
12. Hospital Account Registration and Settings

- Initial registration (<https://www.well-check.co.kr>) 🡪 Access Physician Web 🡪 Set up hospital profile 🡪 Configure other hospital settings (treatment goals, monitoring criteria, test settings)

1. At the First Visit to Physician
2. Connect patient with hospital (Patient App – Physician Web link)
3. Assess patient's medical history and lifestyle habits
4. Recommend tests and provide education for diabetes management
5. Integration of health check-up results
6. Conduct basic surveys and laboratory tests
   During Each Visit -Review patient self-records and provide consultation
7. Understand patient symptoms and lifestyle
8. Blood Sugar Overview: Pre-meal/post-meal trends, estimated changes in HbA1c
9. Review blood sugar/blood pressure/medication records together: Patients using the medication reminder function
10. Analyze medication adherence
11. At Testing – Transmit test results within the hospital
12. Send individual test results and provide counseling
    - Input results from in-hospital measurements (blood sugar/HbA1c, blood lipids, liver function, body composition) into the Physician Web and send to patients, with cumulative management of past records
13. Calculate cardiovascular disease complication risk:
     Input cholesterol and blood pressure values
14. Review health check-up results
15. Post-Consultation Management
16. Provide diabetes education content (twice a week)
    - Cover disease information, dietary habits, exercise, and complication management
    - Track completion rates of educational content
17. Monitoring high-risk patients (weekly)

- Set management goals and alert criteria for blood sugar, blood pressure, and weight; monitor and manage weekly if targets are exceeded
- Utilize “Send Message” and “Teleconsultation” features

1. Monitor adherence to self-recording of blood sugar, blood pressure, and weight (weekly)

- Identify and encourage those who have not entered records, by sending encouragement messages (managed patients should enter self-records at least once a week, check for complications monthly)

## Use of Investigational Software

In this clinical study, the use of 'Well Check' by study subjects will be fully determined by the investigator (physician) based on the subject's medical condition, within the functional scope of the investigational software in a real-world clinical environment.

#

# Data Management

## Source Documents

Source documents are defined as records and materials resulting from data collection activities and observations in this study. They include, but are not limited to, the following clinical trial-related materials.

1. Source document data and records: Subject information sheets and consent forms, medical records, laboratory test records, records held by the pharmacy and laboratory departments involved in the clinical study, etc.
2. All original records or certified copies of original records containing clinical findings, observations, or other activities necessary for the replication or evaluation of the clinical study.

All source documents in this study will be recorded and stored by the investigators at the study site. Access to source documents will be restricted to authorized personnel only.

## Data Entry

The sponsor or delegated personnel will manage the data according to the Standard Operating Procedures (SOP). Monitors will ensure that the entries in the eCRF match the source documents and that the information is appropriate. They will take measures to prevent further collection or additional modifications of the eCRF. Data managers will check the appropriateness of the data entered in the eCRF and verify that it is correctly entered into the database, checking for any logical inconsistencies in the database.

Data for this study will be collected using a pre-designed eCRF. All Electronic Data Capture (EDC) systems used will comply with the Code of Federal Regulations (CFR) (21 CFR Part 11) and guidelines for processing and managing electronic data in clinical studies. The EDC system is an accredited electronic data collection system accessible only by authorized personnel. The system will record all traceable activities, including eCRF data entry, modification, storage, and deletion. The sponsor may request verification or correction of the collected data during the processing. The investigator is responsible for responding to these requests by rechecking or correcting the data and ensuring the data entered into the eCRF is accurate, complete, decipherable, and timely through electronic signatures. After the study concludes, copies of the eCRFs created through the EDC system will be stored on electronic media and provided to each study site. They will be archived under the same conditions as other essential documents.

## Data Verification

In case of discrepancies between the eCRF and source documents, inappropriate entries, or logical inconsistencies, the sponsor or data manager, along with the principal investigator, will review the validity of the concerned items. If necessary, corrections will be made and documented accordingly.

Once the sponsor verifies that there are no errors in the eCRF and the database, the database will be locked to prevent any unauthorized or accidental changes. Once locked, the clinical study data cannot be modified arbitrarily.

## Data Storage

The principal investigator is responsible for storing and managing all collected data and records (including electronic documents) at each site, including those from subjects who withdrew consent or dropped out.

All documents collected during the study must be stored in a secure, locked cabinet accessible only to the principal investigator, co-investigators, and delegated study personnel.

According to Article 15 of the Enforcement Rules of the Bioethics and Safety Act, the principal investigator must retain all documents for three years from the end date of the clinical study (However, the sponsor may extend the storage period if deemed necessary).

These documents are subject to audits by the sponsor or relevant regulatory authorities, and the investigator must not destroy any documents related to the clinical study without the sponsor's written consent. The investigator must seek preventive measures to avoid any accident or early destruction of such documents.

Upon the expiration of the retention period, paper documents will be shredded immediately, and electronic documents will be destroyed in a manner that prevents their recovery or restoration, as per the sponsor's agreement.

## Access to Data

Sponsor, monitor and auditor involved in this study may have access to the subject’s records for the purpose of monitoring and auditing the study and managing the progress. By entering into the study agreement, the investigator acknowledges that monitors and auditors from the sponsor and/or Contract Research Organization (CRO) may review the study subjects' charts and case report forms.

This information must be kept confidential and facilities with appropriate confidentiality standards and management protocols must be in place. The investigator must provide the necessary support to the sponsor and/or CRO. The investigator must also grant authorized representatives from the sponsor, regulatory authorities, and IRB direct access to the original medical records of the study subjects for the verification of study procedures and data.

# Evaluation Criteria and Methods, and Statistical Analysis Methods

## Endpoints

1. **Efficacy Endpoints**
2. **Primary Efficacy Endpoint**

- Percent change and change of HbA1c at 24 weeks compared to baseline

1. **Secondary Efficacy Endpoints**
   1. Percent change and change of FPG at 6, 12, 18, and 24 weeks compared to baseline
   2. Percent change and change in HbA1c at 6, 12, and 18 weeks compared to baseline
   3. Proportion of subjects achieving HbA1c < 7% at 6, 12, 18, and 24 weeks compared to baseline
   4. Proportion of subjects achieving HbA1c < 6.5% at 6, 12, 18, and 24 weeks compared to baseline
   5. Proportion of subjects achieving therapeutic response [change in HbA1c (baseline HbA1c – HbA1c at each evaluation time point) > 0.5% or HbA1c < 7%] at 12 and 24 weeks compared to baseline
   6. Change in blood pressure (systolic and diastolic) at 6, 12, 18, and 24 weeks compared to baseline
   7. Change in BMI and weight at 6, 12, 18, and 24 weeks compared to baseline
   8. Proportion of subjects with a reduction of 5% or more in weight and BMI at 6, 12, 18, and 24 weeks compared to baseline
2. **Exploratory Endpoints**
3. Change in lipid levels (total cholesterol, LDL-C, HDL-C, triglycerides) at 6, 12, 18, and 24 weeks compared to baseline
4. Change in liver function markers (AST, ALT, γ-GTP) at 6, 12, 18, and 24 weeks compared to baseline
5. Change in renal function markers (e-GFR, UACR, UGCR) at 6, 12, 18, and 24 weeks compared to baseline
6. Change in body composition analyzer indicators (skeletal muscle mass, body fat mass, body fat percentage, muscle mass, waist/hip circumference, body water, intracellular water, extracellular water, extracellular water ratio, abdominal fat percentage) at 12 and 24 weeks compared to baseline
7. Change in cardiovascular disease complication risk (ASCVD risk) at 12 and 24 weeks compared to baseline
8. **Safety Endpoints**
9. Laboratory tests, vital signs, physical examination results

## Statistical Analysis Method

1. **Definition of Analysis Sets**

### Efficacy Analysis Set

Subjects who meet the inclusion/exclusion criteria, have been enrolled in this study, received the investigational product at least once, and have had their HbA1c level collected post-baseline will be included in the efficacy analysis set.

### Safety Analysis Set

Subjects who meet the inclusion/exclusion criteria, have been enrolled in this study, received the investigational product at least once according to the approved indications, and have undergone safety evaluations will be included in the safety analysis set.

1. **Methods of Analysis**
2. **General Principles**

Continuous variables will be presented as descriptive statistics (number of subjects, mean, standard deviation, median, minimum, maximum), and categorical variables will be presented as frequencies and percentages. Unless otherwise specified, all tests will be two-sided with a significance level of 5%. All p-values will be reported to a maximum of three decimal places. If not exactly divisible, values will be rounded to two decimal places, rounding up at the third decimal place.

If missing data is minimal and evenly distributed, a complete-case analysis will be considered; multiple imputation methods will be employed if missing data is substantial or disproportionately distributed between groups. Sensitivity analyses will also be performed to ensure the robustness and validity of conclusions under various missing-data scenarios.

1. **Demographic and Baseline Characteristics**

For the demographic data (age, gender, etc.) and baseline characteristics of the study subjects, continuous variables will be presented as mean, standard deviation, median, minimum, and maximum values, while categorical variables will be presented as frequency and percentage.

To assess baseline comparability between intervention and control groups, standardized mean differences (SMDs) will be calculated for key baseline characteristics, including age, sex, HbA1c, BMI, and diabetes duration. Variables exhibiting significant imbalance between groups (SMD > 0.1) will be included as covariates in subsequent analyses.

1. **Efficacy Endpoints**

### Primary Efficacy Endpoints

For the change and percent change in HbA1c, descriptive statistics will be presented at baseline and at 24 weeks. The change from baseline to 24 weeks will be analyzed using mixed-effects models or generalized estimating equations (GEE) to account explicitly for clustering effects. Patient-level covariates identified as significantly imbalanced at baseline (SMD > 0.1) will be included as fixed effects, and inter-center variability as random effects.

### Secondary Efficacy Endpoints

For each secondary efficacy endpoint, descriptive statistics will be presented at baseline, 12 weeks, and 24 weeks. The change from baseline to 12 weeks or 24 weeks will be analyzed using mixed-effects models or GEE to account explicitly for clustering effects. Patient-level covariates will be included as fixed effects, and inter-center variability as random effects.

### Exploratory Endpoints

For each exploratory endpoint, descriptive statistics will be presented at baseline, 12 weeks, and 24 weeks. The change from baseline to 12 weeks or 24 weeks will be analyzed using mixed-effects models or GEE to account explicitly for clustering effects. Patient-level covariates will be included as fixed effects, and inter-center variability as random effects. Multiple comparisons within exploratory endpoints will be adjusted using the Benjamini–Hochberg false discovery rate method, with statistical significance defined at q ≤ 0.05.

1. **Safety Endpoints**

### Vital Signs

For vital signs, descriptive statistics will be presented at baseline, 6 weeks, 12 weeks, 18 weeks, and 24 weeks. The change from baseline at 24 weeks or at 6, 12, and 18 weeks will be analyzed using mixed-effects models or GEE to account explicitly for clustering effects. Patient-level covariates will be included as fixed effects, and inter-center variability as random effects.

### Laboratory Tests

For laboratory tests, descriptive statistics will be presented at baseline, 6 weeks, 12 weeks, 18 weeks, and 24 weeks. The change from baseline at 24 weeks or at 6, 12, and 18 weeks will be analyzed using mixed-effects models or GEE to account explicitly for clustering effects. Patient-level covariates will be included as fixed effects, and inter-center variability as random effects.

Additionally, the frequency and percentage of normal (Normal or NCS) and clinically significant abnormal (CS) changes will be summarized. Detailed information on subjects with clinically significant abnormal (CS) items will also be listed.

1. **Subgroup Analysis**

If a subgroup analysis based on the characteristics of the subjects is required, each item can be analyzed similarly to the efficacy and safety evaluation variables (e.g., analysis of primary efficacy evaluation results by gender etc.)

# Ethical Considerations and Administrative Procedures

## Ethics Committee/Institutional Review Board (IRB)

This clinical study must receive prior approval from the IRB for the protocol and all related matters before initiation. The IRB will review the ethical and medical validity of the study and provide written documentation of their decision to the investigator and the study sponsor. Any amendments or changes to the protocol during the study must be submitted to and approved by the IRB.

## Ethical Considerations

This clinical study will be conducted in accordance with the ethical principles based on the Declaration of Helsinki (Ethical Principles for Medical Research Involving Human Subjects) and in compliance with the Bioethics and Safety Act and related regulations, prioritizing the rights, safety, and welfare of the study subjects.

## Quality Assurance and Audit

The sponsor will implement quality assurance and quality control of clinical study data in accordance with the sponsor's standard operating procedures (SOPs) to ensure that the study is conducted, data are generated, recorded, and reported in compliance with the protocol, the Bioethics and Safety Act, and related regulations.

The auditor will conduct audits following the procedures below.

1. Audits will be conducted according to the safety regulations of pharmaceuticals [Appendix 4] and the Good Clinical Practice (GCP) standards to ensure the quality of the clinical study.
2. The auditor will verify that the eCRF is documented, modified, verified, and processed according to appropriate procedures and that the study is being conducted according to the study plan and objectives.
3. The auditor will confirm that the clinical study is conducted in compliance with the Bioethics and Safety Act, related regulations, and SOPs.
4. The auditor will prepare and review the audit report and submit it to the sponsor. Following the prescribed procedures, the audit report will be finalized, and an audit certificate will be issued.
5. If any violations are identified during the audit, the sponsor will take corrective actions and implement measures to prevent recurrence. If the investigator's continuous violations or serious violations are confirmed, the sponsor may suspend the clinical study participation of the relevant study site.

## Subject Informed Consent

The subject information sheet and informed consent form may be used after approval of the IRB. The investigator must obtain consent for the use of information from the study subjects in accordance with the ethical principles based on the Declaration of Helsinki and the Bioethics and Safety Act standards. The investigator must fully explain the study and obtain written consent from the subjects (or legally acceptable representatives) prior to initiating any study-related procedures. Consent must be obtained in a private setting (e.g., consultation room, examination room). The investigator should retain the original signed consent form in the investigator files, and provide a copy of the signed consent form and information sheet to the subject (or legally acceptable representative).

If the study subject has difficulties in communication due to lack of understanding or ability to express their intentions, written consent must be obtained from the subject’s legal representative. If a subject has no legally acceptable representative, a representative will be appointed in the order of spouse, immediate family, and non-immediate family; if there are more than one immediate or non-immediate family members, it should be decided under an agreement. If an agreement is not reached, the eldest shall be the representative.

Even if the legal representative provides consent, the study subject should also sign and date the consent form, if possible.

If the subject or representative is unable to read, a witness (impartial observer) should be present for the entire consent process. The subject or representative verbally consents to participate in the study, signs the consent form if possible, and the witness signs the form to confirm that the information was accurately conveyed to the subject or representative, that it was understood, and that the consent was given voluntarily.

If there are any changes to the subject information sheet and consent form during the study, re-approval from the IRB must be obtained.

## Approval and Amendment of the Protocol

Prior to initiation of the study, the investigator should obtain written approval from the IRB for related documents and procedures, including the protocol, subject information sheet, and informed consent, etc.

Any amendments to the approved study protocol must also be approved by the IRB at each stage of the clinical study. Subjects cannot be enrolled in the clinical study before the protocol is approved.

## Monitoring of Clinical Study Sites

The sponsor will conduct monitoring to protect the rights and welfare of the study subjects, ensure the accuracy, completeness, and verifiability of data reported by the principal investigator, and confirm compliance with the approved study protocol and relevant regulations.

Monitoring will be carried out by designated monitors through regular site visits and telephone contacts. During site visits, monitors will review source documents, investigational software management records, and the status of essential document files. They will also verify study procedures and records, and if any issues or violations are found, they will discuss appropriate corrections and actions with the principal investigator and study personnel.

Monitoring visits will be scheduled at appropriate times through consultation between the monitors, principal investigator, and study personnel. The principal investigator and study personnel will actively cooperate with the monitors, providing access to all source documents for verification against the eCRF.

## Confidentiality and Privacy Protection for Subjects

Study data will be stored in a locked research office. Subject’s medical record numbers and institution-specific enrollment numbers will be kept in separate files under the responsibility of the principal investigator and encrypted to prevent personal identification from the clinical study data. According to the Enforcement Rule of the Bioethics and Safety Act, records related to the clinical study will be stored for three years after the study is completed. After the retention period, documents containing personal information will be destroyed in accordance with Article 16 of the Enforcement Decree of the Personal Information Protection Act. Files containing personal information will be password-protected, and no identifying information about the subjects will be included in any publications of the study results.

## Measures to Protect the Safety of Subjects

Study sites should have the facilities and specialized personnel necessary to conduct this study and should be fully prepared to properly conduct the study.

The investigator should thoroughly check the health of each subject prior to enrollment into the study to ensure that subjects are eligible to participate in the study. Also, the investigator must thoroughly understand the study protocol and conduct the study accordingly.

The treatment and care of the subjects' conditions should be conducted independently of this study and should continue based on clinical judgment during and after the study period.

This study does not present any risks beyond the routine treatment provided in a clinical setting. Therefore, there are no additional risks related to the study, and no extra compensation for the subjects is necessary. Both the Medical Service Act and the professional liability insurance of the principal investigator and relevant institutions provide sufficient protection for both study subjects and participating investigators.

Since this study does not provide investigational drugs to the subjects, any drug-related compensation will follow the existing legal responsibilities for the drugs.

## Utilization and Publication of Study Results

All data and results generated during the study period are owned by the sponsor, who is to report and publish them. The sponsor will prepare a final report of the study results according to the protocol and notify the investigators.

The investigator may not publish, present, or disclose any information related to the study results without prior written consent of the sponsor and should ensure that sub-investigators also follow this rule.

If the investigator intends to present or publish the study results in academic journals, they must obtain approval from the sponsor, who retains the right to review the content before deciding to make it public.

# Principal Investigator

## Information of Principal Investigator

Name: Sang Yeol Lee

Department: Kyung Hee University Medical Center, Department of Endocrinology and Metabolism, Kyung Hee Digital Health Center

Position: Professor, Director

Address: 23 Kyunghee-daero, Dongdaemun-gu, Seoul, 02447, Korea

## Roles and Responsibilities of the Principal Investigator

The principal investigator is responsible for the overall quality management of the study and the protection of the study subjects' safety. He/She will review key study documents, oversee matters arising at each participating site, and coordinate opinions among participating institutions and investigators. Additionally, the investigator is responsible for obtaining IRB approval before conducting the study, reporting any changes or other matters arising during the study period to the IRB, and ensuring the smooth progress of the study until its completion by analyzing data and preparing the final report.

# Appendices

Attachment 1. Names of Study Sites and Investigators

Attachment 2. Recruitment Notice for Study Subjects

Attachment 3. Subject Information Sheet and Consent Form

Attachment 4. Subject Satisfaction Survey

Attachment 5. Medical Staff Satisfaction Survey

Attachment 6. Well Check User Guide for Subjects

# References

1. International Diabetes Federation. IDF Diabetes Atlas, ninth edition, 2019.

2. International Diabetes Federation. IDF Diabetes Atlas, tenth edition, 2021..

3. Korean Diabetes Association. Treatment Guideline for Diabetes, 2019.

4. American Diabetes Association. 9. Cardiovascular disease and risk management: standards of medical care in diabetes—2018. Diabetes care 2018;41:S86-S104.

5. Williams B, Mancia G, Spiering W, Agabiti Rosei E, Azizi M, Burnier M, et al. 2018 ESC/ESH Guidelines for the management of arterial hypertension: The Task Force for the management of arterial hypertension of the European Society of Cardiology (ESC) and the European Society of Hypertension (ESH). European Heart Journal 2018;39:3021-104.

6. Timpel P, Oswald S, Schwarz PEH, Harst L. Mapping the Evidence on the Effectiveness of Telemedicine Interventions in Diabetes, Dyslipidemia, and Hypertension: An Umbrella Review of Systematic Reviews and Meta-Analyses. J Med Internet Res 2020;22:e16791.

7. Cameron JD, Ramaprasad A, Syn T. An ontology of and roadmap for mHealth research. International journal of medical informatics 2017;100:16-25.

8. Ma Y, Cheng HY, Cheng L, Sit JW. The effectiveness of electronic health interventions on blood pressure control, self-care behavioural outcomes and psychosocial well-being in patients with hypertension: A systematic review and meta-analysis. International journal of nursing studies 2019;92:27-46.

9. Jamshidnezhad A, Kabootarizadeh L, Hoseini SM. The effects of smartphone applications on patients self-care with hypertension: a systematic review study. Acta Informatica Medica 2019;27:263.

10. Stevens S, Gallagher S, Andrews T, Ashall-Payne L, Humphreys L, Leigh S. The effectiveness of digital health technologies for patients with diabetes mellitus: A systematic review. Front Clin Diabetes Healthc 2022;3:936752.

11. Littenberg B, MacLean CD. Intra-cluster correlation coefficients in adults with diabetes in primary care practices: the Vermont Diabetes Information System field survey. BMC Medical Research Methodology. 2006;6(1):20. doi: 10.1186/1471-2288-6-20.

12. Lee YL, Lim YMF, Law KB, Sivasampu S. Intra-cluster correlation coefficients in primary care patients with type 2 diabetes and hypertension. Trials. 2020;21(1):530. doi: 10.1186/s13063-020-04349-4.
